# Daily activity patterns influence retinal morphology, signatures of selection, and spectral tuning of opsin genes in colubrid snakes

**DOI:** 10.1186/s12862-017-1110-0

**Published:** 2017-12-11

**Authors:** E. Hauzman, D. M. O. Bonci, E. Y. Suárez-Villota, M. Neitz, D. F. Ventura

**Affiliations:** 1 Departamento de Psicologia Experimental, Instituto de Psicologia, Universidade de São Paulo, Av. Professor Mello Moraes 1721 Bloco A Sala D9 – Butantã, São Paulo, SP CEP 05508-030 Brazil; 2 Instituto Israelita de Ensino e Pesquisa Albert Einstein, São Paulo, Brazil; 3 Instituto de Ciencias Marinas y Limnólogicas, Universidad Austral de Chile, Edificio Emilio Pugin, Campus Isla Teja S/N, 5110236 Valdivia, Chile; 4 Laboratório de Ecologia e Evolução, Instituto Butantan, São Paulo, Brazil; 5 Department of Opthalmology, University of Washington, 750 Republican Street, Box 358058, Seattle, WA 98109 USA

**Keywords:** Serpents, Retina, Visual pigments, Circadian activity pattern, dN/dS, Visual ecology

## Abstract

**Background:**

Morphological divergences of snake retinal structure point to complex evolutionary processes and adaptations. The Colubridae family has a remarkable variety of retinal structure that can range from all-cone and all-rod to duplex (cone/rod) retinas. To explore whether nocturnal versus diurnal activity is responsible for constraints on molecular evolution and plays a role in visual opsin spectral tuning of colubrids, we carried out molecular evolution analyses of the visual opsin genes *LWS*, *RH1*, and *SWS1* from 17 species and performed morphological analyses.

**Results:**

Phylogenetic reconstructions of the *RH1* and *LWS* recovered major clades characterized by primarily diurnal or primarily nocturnal activity patterns, in contrast with the topology for *SWS1*, which is very similar to the species tree. We found stronger signals of purifying selection along diurnal and nocturnal lineages for *RH1* and *SWS1*, respectively. A blue-shift of the RH1 spectral peak is associated with diurnal habits. Spectral tuning of cone opsins did not differ among diurnal and nocturnal species. Retinas of nocturnal colubrids had many rows of photoreceptor nuclei, with large numbers of rods, labeled by wheat germ agglutinin (WGA), and two types of cones: large cones sensitive to long/medium wavelengths (L/M) and small cones sensitive to ultra-violet/violet wavelengths (UV/VS). In contrast, retinas of diurnal species had only one row of photoreceptor nuclei, with four types of cones: large and double L/M cones, small UV/VS cones, and a second group of small cones, labeled by WGA.

**Conclusions:**

For *LWS* gene, selection tests did not confirm different constraints related to activity pattern. For *SWS1*, stronger purifying selection in nocturnal lineages indicates divergent evolutionary pressures related to the activity pattern, and the importance of the short wavelength sensitivity at low light condition. Activity pattern has a clear influence on the signatures of selection and spectral tuning of RH1, with stronger purifying selection in diurnal lineages, which indicates selective pressure to preserve rhodopsin structure and function in pure-cone retinas. We suggest that the presence of four cone types in primarily diurnal colubrids might be related to the gain of color discrimination capacity.

**Electronic supplementary material:**

The online version of this article (10.1186/s12862-017-1110-0) contains supplementary material, which is available to authorized users.

## Background

Recent studies are bringing new light on the nature and evolution of the visual system of snakes [1–6]. After a long period of neglect, snake retinas, and more specifically, their visual pigments and opsin genes, have aroused new interest regarding their features and evolution [1–9]. The suborder Serpentes comprises a highly diversified group of vertebrates, with more than 3500 living species that inhabit a great diversity of ecological niches and exhibit distinct activity patterns, with diurnal, crepuscular and nocturnal species [10–13]. Most vertebrate visual systems have two distinct types of photoreceptor cells, rods and cones, that constitute, respectively, the rod system, which is highly sensitive to light and is responsible for nocturnal (scotopic) vision, and the cone system, which is responsible for mediating high visual acuity and color discrimination in photopic conditions [14–16]. Snakes retinal anatomies are very diverse and were classified based on their photoreceptor morphology as all-cone, all-rod or duplex retinas (cones/rods), with up to three types of cones and a rod [3, 7–9, 17–22]. Most nocturnal snakes have rod-dominated retinas and many diurnal species from the Caenophidia group (“advanced” or “higher” snakes) have pure-cone retinas [1, 7–9, 17–22]. On the other hand, some strictly nocturnal caenophidian snakes have no cone-like photoreceptor, but three rod-like cone classes [7, 8]. In a classic comparative study on the vertebrate retinas, Gordon Walls [7, 8] proposed that cones and rods could be interchangeable in a process he called “transmutation”. In this theory, Walls suggested, in reference to squamate reptiles, that some retinas with rods and cones had originated from pure-cone retinas of a diurnal ancestor, with the conversion of some of the cones into rods, as an adaptation to nocturnal activity (for more detail, see [3, 4, 6]).

Recently, molecular analyses of the opsin genes were performed in *Python regius* and *Xenopeltis unicolor,* two nocturnal henophidian snakes. Three of the five major classes of visual opsin genes of vertebrates are expressed in the retinas of both species: the rhodopsin gene *RH1* in rods, the short wavelength sensitive *SWS1* in small singles cones, and the long wavelength sensitive *LWS* in large single cones [1]. The two remaining typical vertebrate cone opsin genes, *RH2* and *SWS2*, sensitive to middle and short wavelengths, respectively, are not expressed in henophidian retinas and the authors suggested that they might have been lost in the ancestor of extant snakes. Subsequent studies on caenophidian snakes confirmed the absence of the *RH2* and *SWS2* opsin genes [2, 23] and the expression of the same three opsin genes (*LWS, RH1,* and *SWS1*) in retinas of most of the diurnal and nocturnal species studies so far [2–6]. These findings strongly suggest that the ancestral snake had functional copies of the three visual pigment genes and provides evidence of at least some degree of scotopic vision in ancestral snakes [2, 5].

The expression of the rhodopsin RH1 visual pigment in pure-cone retinas of diurnal snakes is intriguing and has stimulated new investigations on the specific function and physiology of the photoreceptor in which it is expressed. Schott and colleagues [4] found evidence for a functional rhodopsin, with an accentuated blue-shift of the absorption peak, expressed in a group of small single cones in the diurnal garter snake *Thamnophis proximus*, also confirmed by Bhattacharyya and colleagues [6], in the all-cone retinas of the diurnal *Pituophis melanoleucus*. Both studies showed evidence of expression of the rod transducin, providing strong support for the hypothesis that the cones that express the RH1 are in fact modified rods. These cone-like rods may have evolved to enable trichromatic spectral discrimination and the potential for a richer color vision, and therefore, compensate for ancestral losses of the middle-wavelength cone opsin (RH2) in early snake evolution [4, 6]. Studies on the spectral sensitivity and molecular analysis of the opsin genes of diurnal and nocturnal elapid and colubrid snakes, led Simões and colleagues [3] to conclude that multiple transmutation events from rod-to-cone and from cone-to-rod occurred in snakes.

Furthermore, molecular evolutionary analysis indicated strong selective constraint in the rhodopsin gene *RH1* of *T. proximus* [4] and on the three opsin genes found in a range of fossorial and non-fossorial snakes [2]. In an extensive survey, Simões and colleagues [5] searched for signals of selection in snake visual opsin genes, in a number of species from different families, with different ecological features and daily activity patterns. The authors found that shifts in the molecular evolution (functional constraint) of visual pigment genes are correlated with many variables, including ecological niche and retinal morphology. They found a lower functional constraint in all visual opsin genes of diurnal snakes with transmuted rod-like cones, which indicates that visual pigment adaptation occurs in association with morphological transmutation of photoreceptors [5].

Based on the diversity of the daily activity patterns and the photoreceptor morphology in snakes from the Colubridae family [3, 7, 8], we used an extensive dataset to explore whether nocturnal versus diurnal activity in this specific group is correlated with opsin spectral tuning and is associated (possibly causally) with patterns of molecular evolution of the *LWS, RH1,* and *SWS1* opsin genes. We used several models to detect selection signals along Colubridae lineages (subfamilies Colubrinae and Dipsadinae) in a phylogenetic framework. We also performed morphological analyses of colubrid snake retinal structure with anti-opsin antibodies and agglutinins to detect distinct photoreceptor types. Because the rhodopsin RH1 photopigment is unusually expressed in a cone-like photoreceptor in diurnal colubrids, we hypothesized that diurnal activity has had a major effect on the evolution of *RH1* and the spectral tuning of its corresponding photopigment. Our findings indicate that daily activity pattern has major effects on the evolution of the three opsin genes and on the retinal structure, i.e. the thickness of the outer nuclear layer and the photoreceptor morphology of diurnal and nocturnal colubrid snakes.

## Methods

### Sample information

Animal procedures were in accordance with ethical principles of animal management and experimentation established by the Brazilian Animal Experiment College (COBAE) and were approved by the Ethics Committee of Animal Research of the Butantan Institute, São Paulo, Brazil (777/10). Single colubrid specimens, of representative species of the Colubrinae (*n* = 2) and Dipsadinae (*n* = 15) subfamilies, were provided by the Butantan Institute (Table 1). The snakes were euthanized with a lethal injection of 30 mg/kg of sodium thiopental (Thionembutal). The voucher specimens were fixed and deposited in the Herpetological Collection of the Butantan Institute.

**Table 1:** Species, daily activity pattern and habitat

Family	Subfamily	Tribe	Species	Activity pattern	Habitat
Colubridae	Colubrinae		*Chironius bicarinatus*	D	Ar
			*Spillotes pullatus*	D	Ar
	Dipsadinae		*Atractus reticulatus*	N	Fs
			*Dipsas petersi*	N	Ar
			*Sibynomorphus mikanii*	N	Te
			*Sibynomorphus neuwiedi*	N	Ar
		Xenodontini	*Erythrolamprus aesculapii*	D	Te
			*Erythrolamprus miliaris*	D	Aq/Te
			*Erythrolamprus poecilogyrus*	D	Te
		Pseudoboini	*Oxyrhopus guibei*	N	Te
		Hydropsini	*Helicops modestus*	D	Aq
		Echinantherini	*Echinantera cephalostriada*	D	Te
			*Echinanthera undulata*	D	Te
			*Taeniophallus persimilis*	D	Te
		Philodryadini	*Philodryas patagoniensis*	D	Te
		Tachymenini	*Tomodon dorsatus*	D	Te
			*Thamnodynastes hypoconia*	D	Te

Activity pattern and species habitat were stablished based on [12, 13]

*D* primarily diurnal, *N* primarily nocturnal, *Aq* aquatic, *Ar* arboreal, *Fs* fossorial, *Te* terrestrial

### RNA extraction, sequencing and sequence alignment

Following enucleation, eyes were preserved in RNA*later*® (Life Technologies, Carlsbad, CA, USA) at 4 °C. Total RNA was extracted from homogenized retinas using the RNase Mini Kit (Qiagen GmbH, Hilden, Germany) according to the manufacturer’s instructions and preserved at −80 °C. Total RNA was diluted 10 fold and mRNA was converted to complementary DNA (cDNA) using 500 ng of oligo dT primer and Superscript III reverse transcriptase (Life Technologies, Carlsbad, California, USA), following the manufacturer’s protocol.

Primers were designed based on the visual opsin sequences of *P. regius* and *X. unicolor* (GenBank accession numbers FJ497233 - FJ497238), to amplify the *LWS, RH1,* and *SWS1* genes, using Primer 3 (v.0.4.0) [24]. Additionally, more specific primers were designed to amplify colubrid opsin genes, based on the initial sequencing results obtained with the previous primers. Primer information is provided in Additional file 1: Table S1. Polymerase chain reactions (PCRs) were carried out using High Fidelity Platinum Taq Polymerase, 10× High Fidelity Buffer and MgCl_2_, 10 mM GeneAmp dNTPs (Life Technologies, Carlsbad, California, USA) and 20 μM primers in 50 μL reactions. The PCR conditions were: 1) an initial denaturation at 94 °C for 1 min; 2) 37–50 cycles of 15 s at 94 °C, 30 s at the annealing temperature (Additional file 1: Table S1) and 30 s at 72 °C; 3) a final extension at 72 °C for 10 min. PCR products were visualized by electrophoresis in 1.0% agarose gel and purified with Illustra GFX™ PCR DNA and Gel Band Purification Kit (GE Healthcare, Little Chalfont, Buckinghamshire, UK). PCR products were directly sequenced in both directions with BigDye® Terminator v3.1 Cycle Sequencing Kit (Applied Biosystems) and the 3500 Applied Biosystems Sequencer. Electropherograms were visualized and aligned with Geneious v.9.1.3 (GeneMatters Corp.), using the iterative method of global pairwise alignment (MUSCLE and ClustalW) implemented in the same software [25, 26]. This alignment included sequences generated in this work and sequences download from GenBank of 36 Colubridae species, including the Colubrinae and Dipsadinae subfamilies [5], 2 henophidian species [1], and 92 opsin sequences from other vertebrates, representing the five main classes of visual pigments (LWS/MWS, RH1, RH2, SWS1, and SWS2; Additional file 1: Table S2; Table S3). A partial sequence of the melanopsin gene *OPN4x* from red-eared slider turtle (*Trachemys scripta elegans*) was also included in the alignment and was used as outgroup (GenBank accession number: JN815264.1).

### Opsin gene tree reconstruction

Phylogenetic analysis was performed on a codon-match nucleotide alignment. All codon positions were included and gaps were treated as missing data. Using PartitionFinder v.1.1.1 [27], the models TVM + I + G, GTR + G, and GTR + I + G, for codon positions 1, 2, and 3, respectively, were determined as the best-fit models of substitution and were used to perform Maximum Likelihood (ML) and Bayesian inferences (BI). ML reconstruction was carried out with Garli v2.0 [28] and statistical support for branches was estimated by non-parametric bootstrap [29], with 1000 pseudo replications. BI was performed using MrBayes 3.2 [30, 31]. The Markov Chain Monte Carlo (MCMC) was initiated from a random tree, sampling every 1000 generations from two parallel runs of 1.0 × 10^7^ generations. Each run and the independent log and tree files were combined using LogCombiner version 1.8 [32]. Through Tracer v1.5 [33], the stationary phase was checked following Nylander et al. [34]. Points sampled before the plateau phase were discarded as burn-in (25% of the trees), and the remaining trees were combined to find the maximum a posteriori probability estimate of the phylogeny, using TreeAnnotator v1.8 [32]. The resulting tree were viewed and edited using the program Figtree v1.4.2 (available from: http://tree.bio.ed.ac.uk/software/figtree/).

### Statistical analysis of molecular evolution

We investigated the presence and type of selection acting on the opsin genes *LWS*, *RH1*, and *SWS1* of primarily diurnal and primarily nocturnal colubrid snakes by employing a codon-based method, using the codeml program from PAML v.4.7 [35]. We estimated the ratio of nonsynonymous (dN) to synonymous (dS) substitutions using branch model, branch-site model, and site model analyses. The dN/dS ratio (ω) indicates the type and magnitude of selection acting on the gene, where ω < 1 indicates purifying selection, ω ~ 1 indicates neutral evolution and ω > 1 indicates positive selection [35, 36]. Branch and branch-site model analysis allow appointing specific branches of interest in the trees as “foreground” branches and compare their ω rates with the ω estimated for the “background” branches [35]. Branch-site and site-model analysis allow ω to vary among codon sites and to detect codon sites potentially under positive selection. For molecular evolution analyses, we used a phylogenetic tree congruent with those published previously [37–39]. When inconsistences were found among the phylogenies, we adopted that proposed by Pyron et al. [38], which used a broader species sampling. For species sampled in this study that were not included in these previous phylogenetic studies (i.e. *Dipsas petersi*, *Echinanthera cephalostriata* and *Taeniophallus persimilis*), we assumed the phylogenetic position reported for the corresponding genus. The species tree used in these analyses is presented in Additional file 1: Figure S1.

In Codeml, we used likelihood ratio tests (LRTs) to compare competing models of gene evolution. The LRT statistic was computed as 2log likelihood difference between the two models and was tested against the χ^2^ distribution, where the degrees of freedom equal the difference between the number of parameters in the two nested models [35]. We also applied comparisons using the Bayesian information criterion (BIC), which was computed as -2 l + K log *n*, where K is the number of estimated parameters and *n* is the sample size. The model associated with the lowest BIC score was considered the best [40–42]. We applied branch models to test for selection in diurnal and nocturnal lineages, by running several multiple ratio *models* designating different foreground branches and comparing them among each other. The *null model* assumes the same ω ratio for all branches, while the *free ratio model* assumes independent ω ratios for each branch. The *2ω model* assumes a ω_0_ for non-snakes and a ω_1_ for all snake lineages. The *3ω model* assumes a ω_0_ for non-snakes, a ω_1_ for henophidian snakes (*Python regius* and *Xenopeltis unicolor*) and a ω_2_ for colubrid snakes. The *4ω model* has one fixed ω for non-snakes, a ω_1_ for henophidian snakes, and distinct ω values for the two sampled colubrid subfamilies, Colubrinae and Dipsadinae. A second *4ω model* for testing the effects of the daily activity pattern assumes one fixed ω for non-snakes, a ω_1_ for henophidian snakes, a ω_2_ for primarily diurnal and a ω_3_ for primarily nocturnal colubrid snakes.

To analyze whether diurnal or nocturnal lineages might have experienced positive selection on any codon site, we used branch-site models [35] and implemented Model A (Model = 2, NSsites = 2), as an extension of the site-specific “neutral” model (M1) of Nielsen & Yang [43]. The *null models* are the same as for Model A but with ω_2_ fixed at 1 for foreground branches. The proportions *p*_0_ and *p*_1_ as well as the ratio ω_2_ were estimated from the data by maximum likelihood [44]. We also applied site-specific models to test for heterogeneous selection pressure among codon sites across all branches of the tree. For site-specific models, we used only the snake sequences data set, excluding the outgroup of non-snake vertebrates. Comparisons between site models were used to test for variation in ω (M3 vs. M0) and for the presence of positively selected sites (M2a vs. M1a and M8 vs. M7) [35, 45]. If LRTs for branch models and site-specific models were significant for positive selection, we used Bayes Empirical Bayes (BEB) to calculate posterior probabilities for site classes and to identify the amino acid sites likely under positive selection [35].

### Prediction of spectral tuning of Opsin proteins

Predictions of the wavelength of absorption peak (λ_max_) of visual pigments were made based on particular combinations of critical (tuning site) amino acids in the visual opsins described in the literature for vertebrates. All amino acid residues were numbered based on the bovine rhodopsin sequence (GenBank accession number NM001014890). The prediction of the LWS pigment spectral peak was estimated based on the residues at five spectral tuning sites: 164, 181, 261, 269 and 292 [46]. The amino acid combination S164, H181, Y261, T269 and A292 is known to generate a long wavelength sensitive photopigment with λ_max_ at ~560 nm [46] in different vertebrate lineages, and the substitutions S164A, H181Y, Y261F, T269A and A292S are generally responsible for downward shifts of 7, 28, 8, 15 and 27 nm, respectively [46, 47]. For the rhodopsin RH1, we examined 13 amino acid sites that have been linked to the spectral tuning in different vertebrates: 83, 90, 113, 118, 122, 164, 180, 261, 265, 269, 285, 292 and 299 [48–51]. The λ_max_ of the SWS1 was predicted based on the amino acid residue at site 86 [52–55], which has been shown to be critical in determining the peak sensitivity in all non-avian SWS1 photopigments [54]. The amino acid F86 confers UV sensitivity to the SWS1 pigment, with λ_max_ at ~360 nm [52, 53]. Besides residue 86, we also analyzed 11 other amino acid residues that had been related to the tuning of violet sensitive SWS1 photopigments, but with no major effects on the shift from UV to violet: 46, 49, 52, 90, 93, 97, 113, 114, 116, 118, 265 [52, 55, 56].

### Retinal morphology analysis

We analyzed retinal structure using immunohistochemistry. Radial sections of 11 species were labeled with anti-opsin antibodies and the agglutinins WGA and PNA: the primarily diurnal *Chironius bicarinatus*, *Erythrolamprus miliaris*, *E. poecilogyrus*, *Helicops modestus*, *Thamnodynastes hypoconia* and *Tomodon dorsatus*; and the primarily nocturnal *Atractus reticulatus*, *Dipsas petersi*, *Oxyrhopus guibei*, *Sibynomorphus mikanii* and *S. neuwiedi*.

Eyecups were fixed for 3 h in 4% paraformaldehyde (PFA) diluted in phosphate buffer saline 0.1 M (PBS) and cryoprotected with 30% sucrose solution diluted in PBS, for 48 h. Radial sections at 12-μm thicknesses were obtained in cryostat (CM1100 Leica, Nussloch, Germany), collected onto gelatinized glass slides and stored at −20 °C until use. Retinal sections were incubated overnight with polyclonal antibodies against blue opsin (Chemicon International, Hofheim, Germany, cat. n. AB5407; 1:100) and red/green opsin (Chemicon International, cat. N. AB5405; 1:300), diluted in 0.1 M PBS with 0.3% Triton X-100. The antibodies were raised initially in rabbit with recombinant human blue opsin against the last 42 amino acids at the C-terminus, and red/green opsin against the last 38 amino acids at the C-terminus [57]. The specificity of both antibodies for colubrid snake retinas was described previously [22]. The tissues were incubated in the secondary antibody goat anti-rabbit immunoglobulin G (whole molecule; 1:200; Jackson Immunoresearch Laboratories, West Grove, Pa., USA), conjugated with the fluorescent molecule rhodamine (TRICT), diluted in 0.1 M PBS with 0.3% Triton.

Retinal sections were also incubated with fluorescein isothiocyanate (FITC)- conjugated peanut agglutinin (PNA; 1:500; Vector Labs, Burlingame, CA) and with rhodamine (TRITC)-conjugated wheat germ agglutinin (WGA, 1:2000; Vector Labs, Burlingame, CA), to mark cones and rods outer segments, respectively [58]. The slides were mounted using Vectashield with 4,6-diamidino-2-phenylindole (DAPI; Vector, Burlingame, CA) and observed under a fluorescent microscope (Leica DMRXE). Detailed procedures of the immunohistochemistry techniques and antibodies characterization are described elsewhere [22, 57, 58].

## Results

### Phylogenetic reconstruction

**Fig. 1:**
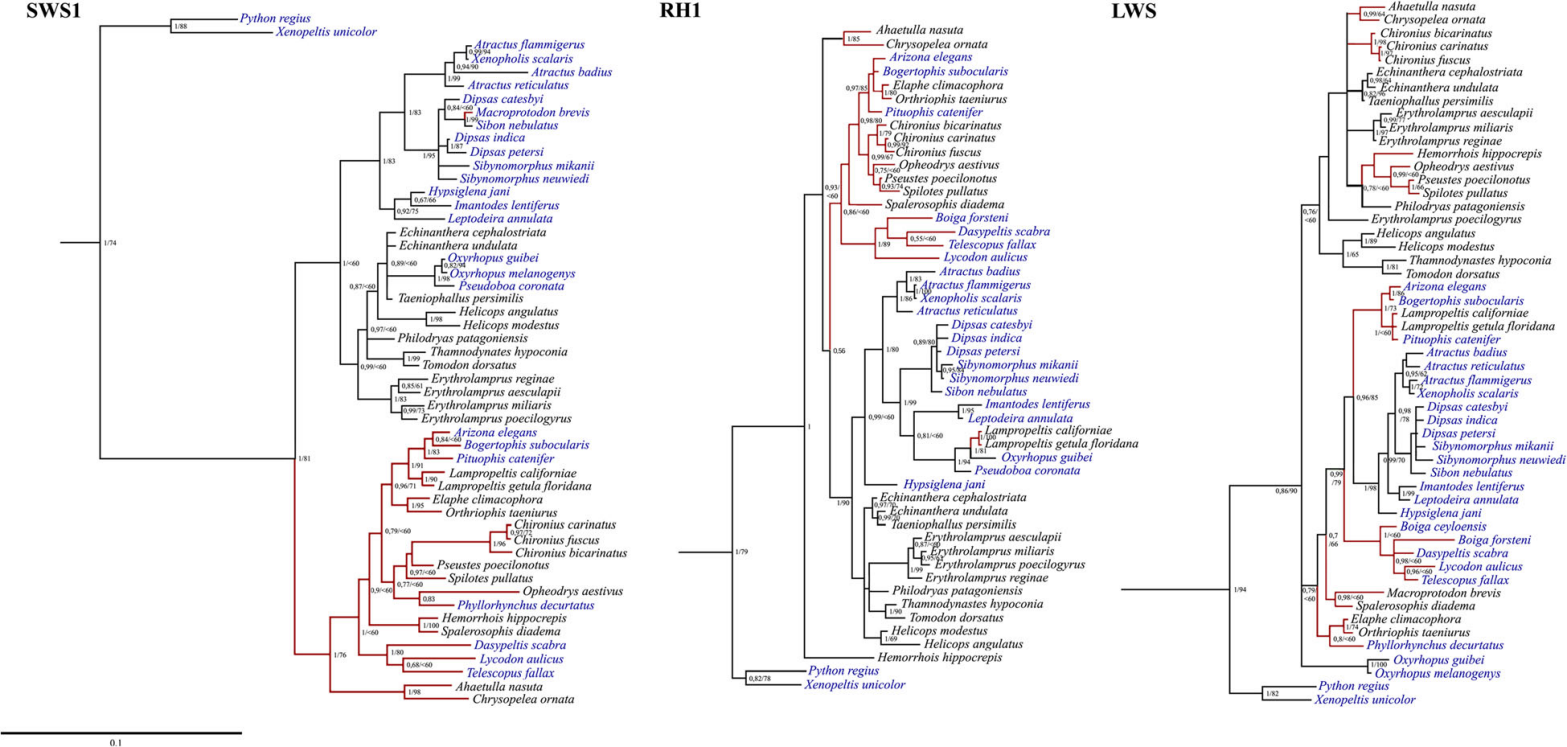
Results of phylogenetic analyses of the nucleotide sequences of the *SWS1*, *RH1*, and *LWS* snake opsin genes, constructed by Maximum Likelihood (ML). Primarily diurnal species are represented in black, and not primarily diurnal species are represented in blue. The brown lines and the black lines represent species from the Colubrinae and the Dipsadinae subfamilies, respectively. ML bootstrap support and BI posterior probability are indicated for each resolved internal branch, in the order ML/BP. Values below 0.7/60 are not depicted. The species used as outgroup are listed in Additional file 1: Table S2. Scale bars represent the number of nucleotide substitutions per site

Using cDNA derived from ocular mRNA of 17 colubrid snakes (Table 1), partial sequences of the *RH1, LWS* and *SWS1* opsin genes (~1000 pb) were successfully amplified. The nucleotide and amino acid alignments of the three opsin coding sequences of the colubrid snakes together with the henophidians *P. regius* and *X. unicolor* and the bovine rhodopsin gene, are available in Additional file 2: Figure S3. The final alignment used for phylogenetic reconstruction was 807 base pairs long. All sequence data have been deposited at NCBI GenBank under accession numbers MG544927-MG544977 (Additional file 1: Table S3). The BI and ML reconstructions recovered the monophyly of each of the five opsin groups, with high support (data not show). The inferred trees confirmed the identities of the Colubridae *LWS, RH1*, and *SWS1* opsin genes, sister to the orthologous opsins of henophidian snakes (Fig. 1). The inferred *SWS1* tree was highly consistent with published snake phylogenies and recovered the monophyly of almost all sampled colubrine colubrids (BP/bootstrap: 1.0/76). Nevertheless, *Macroprotodon brevis*, a primarily diurnal colubrine, was recovered with primarily nocturnal dipsadine (BP/bootstrap: 1.0/83). The *LWS* and *RH1* trees are generally less well supported and recovered non-monophyletic Colubrinae and Dipsadinae, with species instead grouped mainly by nocturnal and diurnal activity patterns (Fig. 1). Notably, in the *RH1* and *LWS* trees, the primarily diurnal colubrines *Lampropeltis californiae* and *L. getula floridana* are nested within a group of nocturnal dipsadines and colubrines.

### Molecular evolutionary analysis

The branch model analysis indicates that the overall estimates of ω under the *null model* (*1ω ratio*) were low for the three opsin genes (0.15, 0.11 and 0.07, for *LWS, RH1* and *SWS1*, respectively; Table 2). In all cases, the *free model* provided a significantly better fit, indicating the heterogeneity of ω values among lineages (Table 3). For the *RH1* and *SWS1* genes, the best intermediate branch-models were the *4ω model*, with independent ω values for primarily diurnal and primarily nocturnal colubrids (Table 3). For the *RH1* gene, the ω value for diurnal lineages was 2.4 times lower than the value for nocturnal lineages (0.161 vs. 0.391; Table 2). On the other hand, for the *SWS1* gene the ω value for diurnal lineages was 1.6 times higher than that of nocturnal lineages (0.128 vs. 0.081; Table 2). For the *LWS* opsin gene data set, the best model was that considering independent ω values for Colubrinae and Dipsadinae (Table 2).

**Table 2:** Parameter estimates and log likelihood values under different branch models for the snakes’ opsin genes

					ω values			
	Model	ℓ	p	BIC	Non-snakes	Henophidian Snakes	Colubridae Snakes	
							Colubrinae	Dipsadinae
LWS	1ω	−11,887.4	149	24,154.6	0.151	0.151	0.151	0.151
	2ω	−11,778.4	150	23,939.1	0.094	0.414	0.414	0.414
	3ω	−11,765.1	151	23,915.0	0.094	0.149	0.498	0.498
	4ω	−11,761.3	152	23,910.0	0.094	0.171	0.659	0.395
							Diurnal	Nocturnal
	4ω (D x N)^*^	−11,764.5	152	23,916.4	0.094	0.148	0.549	0.450
	FM	−11,567.6	295	23,887.3	–	–	–	–
RH1	1ω	−11,225.5	145	22,820.7	0.107	0. 107	0. 107	0. 107
	2ω	−11,165.1	146	22,702.3	0.071	0.231	0. 231	0. 231
	3ω	−11,162.1	147	22,698.9	0.071	0.137	0.250	0. 250
	4ω	−11,161.0	148	22,699.3	0.071	0.157	0.213	0.299
							Diurnal	Nocturnal
	4ω (D x N)^*^	−11,150.9	148	22,679.0	0.071	0.154	0.161	0.391
	FM	−11,012.3	287	22,756.1	–	–	–	–
SWS1	1ω	−14,413.9	153	29,217.9	0.065	0.065	0.065	0.065
	2ω	−14,394.6	154	29,181.8	0.051	0.093	0.093	0.093
	3ω	−14,388.9	155	29,172.8	0.051	0.047	0.102	0.102
	4ω	−14,385.6	156	29,168.8	0.051	0.046	0.122	0.082
							Diurnal	Nocturnal
	4ω (D x N)^*^	−14,384.7	156	29,167.0	0.051	0.047	0.128	0.081
	FM	−14,165.7	303	29,103.7	–	–	–	–

^*^D x N, primarily diurnal and primarily nocturnal lineages, with independent ω values

*FM* free model, *l* likelihood value

**Table 3:** Summary of Likelihood Ratio Test (LRTs) for selection tests

Opsins	Comparisons	2ΔLn L	Df	*P* value	Positive Sites
LWS	Branch Models				
	free model x 1ω	639	146	<10^−5^	–
	2ω x 1ω	218	1	<10^−5^	–
	3ω x 2ω	27	1	<10^−5^	–
	4ω x 3ω	8	1	0.006	–
	4ω (D x N) x 3ω	1	1	0.28	–
	Branch-Site Models				
	Model A (diurnal) x null	45.9	2	<10^−5^	39 L(0.97) 49 V(0.89) 112 L(0.81) **119A**(0.99) 165C(0.95) 195S(0.97) **213A**(0.99) **276 V**(1.0)
	Model A (nocturnal) x null	100.9	2	<10^−5^	39 L(0.94) 119A(0.73) **124A**(0.99) 137 V(0.82) 146 V(0.98) **166A**(0.99) **199G**(1.0) 209I(0.79) **213A**(1.0) **218 V**(1.0) 221I(0.85) **269 T** ^*^(1.0) 270F(0.77) **282A**(0.99) 290 L(0.97)
	Site Models				
	Model 3 x Model 0	726	4	<10^−5^	–
	Model 2a x Model 1a	156	2	<10^−5^	**39 L**(1.0) 49 V(0.95) **104 V**(0.99) **112 V**(1.0) **119 V**(1.0) 124A(0.98) **154 V**(1.0) 158 L(0.87) 164S^*^(0.6) **165C**(1.0) **166A**(1.0) 195S(0.97) **205 V**(0.99) 209I(0.98) **213A**(0.99) 218 V(0.94) 228 L(0.87) 269 T^*^(0.79) 270F(0.7) **290 L**(1.0)
	Model 8 x Model 7	172	2	<10^−5^	**39 L**(1.0) **45 V**(0.99) **49 V**(0.99) 81I(0.83) **104 V**(1.0) 109 L(0.69) **112 L**(1.0) **119A**(1.0) **124A**(1.0) 137 V(0.88) 146 V(0.96) **154 L**(1.0) 158 L(0.98) 164S^*^(0.98) **165C**(1.0) **166A**(1.0) **195S**(0.99) 198P(0.95) 199G(0.9) **205 V**(1.0) 206 V(0.85) **209I**(0.99) **213A**(1.0) 214I(0.8) **218 V**(1.0) 221I(0.97) **228 L**(0.99) **269 T** ^*^(0.99) **270F**(0.99) 276 V(0.87) 287 T(0.9) **317 L**(1.0)
RH1	Branch Models				
	free model x 1ω	427	142	<10^−5^	–
	2ω x 1ω	121	1	<10^−5^	–
	3ω x 2ω	6	1	0.015	–
	4ω x 3ω	2	1	0.14	–
	4ω (D x N) x 3ω	22	1	<10^−5^	–
	Branch-Site Models				
	Model A (diurnal) x null	8.5	2	0.01	**155 M**(0.99)
	Model A (nocturnal) x null	49.5	2	<10^−5^	159 L(0.9) 213 V(0.7) **214 T**(1.0) **292S** ^*^(1.0)
	Site Models				
	Model 3 x Model 0	427	4	<10^−5^	–
	Model 2a x Model 1a	29	2	<10^−5^	81 V(0.8) 112 V(0.7) 133 V(0.8) 158S(0.9) 159 L(0.8) 209 L(0.7) **213 V**(0.99) **217 T**(0.99) 290 V(0.96) 292S^*^(0.7) 299A^*^(0.9)
	Model 8 x Model 7	35	2	<10^−5^	46 L(0.8) 60Y(0.66) 81 V(0.9) 83 N^*^(0.7) 107 T(0.7) 112 V(0.9) 133 V(0.9) 158S(0.98) 159 L(0.9) 169A(0.6) 173I(0.8) 185S(0.6) 209 L(0.9) **213 V**(1.0) **217 T**(1.0) **290I**(0.99) 292S^*^(0.9) 299A^*^(0.97)
SWS1	Branch Models				
	free model x 1ω	496	150	<10^−5^	–
	2ω x 1ω	39	1	<10^−5^	–
	3ω x 2ω	12	1	<10^−4^	–
	4ω x 3ω	7	1	0.01	–
	4ω (D x N) x 3ω	8	1	0.004	–
	Branch-Site Models				
	Model A (diurnal) x null	46.6	2	<10^−5^	57I(0.97) 86 V^*^(0.75) 91I(0.97) 118 T^*^(0.9) 122 L(0.9) 143 L(0.96) 178Y(0.76) 213 L(0.8) 217 T(0.8) 255 V(0.6) 258I(0.6) **282G**(1.0) 315A(0.8)
	Model A (nocturnal) x null	21.3	2	<10^−5^	105 L(0.68) 109 V(0.87) 124I(0.9) 137I(0.8) 149 N(0.9) **213 L**(0.99) 259G(0.98)
	Site Models				
	Model 3 x Model 0	437	4	<10^−5^	–
	Model 2a x Model 1a	0	2	1.0	–
	Model 8 x Model 7	0	2	1.0	–

Note – Underlined model fits the data significantly better. Sites inferred to be under positive selection at the 99% level are listed in bold; numbers after each site indicate the posterior probability value

^*^Known spectral tuning sites. Sites are numbered based on the bovine RH1

The LRT comparisons of branch-site models indicate that both *models* designating diurnal or nocturnal lineages as foreground branches fit the data significantly better than the *null model* (Table 3) for the three opsin genes. For the *LWS* gene BEB analysis indicated several sites under selection in diurnal (ω_2_ = 34.3, p_2_ + p_3_ = 0.03; Additional file 1: Table S4) and nocturnal lineages (ω_2_ = 14.8, p_2_ + p_3_ = 0.06; Additional file 1: Table S4), including the spectral tuning site 269. For the *RH1* gene BEB analysis indicated only one site under positive selection in the diurnal lineage (ω_2_ = 4.4, p_2_ + p_3_ = 0.007; Table 3; Additional file 1: Table S4), and four sites in the nocturnal lineage, including residue 292, a known RH1 spectral tuning site (ω_2_ = 11.4, p_2_ + p_3_ = 0.02; Table 3; Additional file 1: Table S4). For the *SWS1* data set, BEB analysis identified a number of sites under positive selection in both diurnal and nocturnal lineages (ω_2_ = 1.2, p_2_ + p_3_ = 0.078 and ω_2_ = 1.7, p_2_ + p_3_ = 0.046, respectively; Table 3; Additional file 1: Table S4), including the SWS1 spectral tuning sites 86 and 118.

The LRT comparison of site-specific models showed a significant difference between M0 and M3 models for the three opsin genes, indicating that relative rates of substitution are variable among sites (Table 3). For the *LWS* and *RH1* genes, models M2a and M8 fit the data significantly better than the alternative models (*P* < 0.00001; Table 3). Under M2a and M8 several sites were indicated by BEB as under positive selection (Table 3; Additional file 1: Table S4), including residues 164 and 269, known to be involved in the spectral tuning of the LWS photopigment, and residues 83, 292 and 299, involved in the spectral tuning of the RH1 photopigment (Table 3). For the cone opsin gene *SWS1*, the models incorporating positive selection (M2a and M8) do not fit the data significantly better than the alternative models (M1a and M7).

### Predicted spectral tuning of Opsin proteins

Most primarily diurnal colubrines and dipsadines species have the amino acids serine, histidine, tyrosine, threonine and alanine (SHYTA) at the key tuning sites of the LWS photopigment, and thus a predicted λ_max_ at ~560 nm (Additional file 1: Table S5). In the nocturnal dipsadines *Atractus reticulatus*, *Dipsas indica*, *Sibynomorphus mikanii* and *S. neuwiedi*, we observed the double substitution S164A/T269A, which is presumed to generate a medium wavelength sensitive visual pigment with λ_max_ at ~537 nm [46] (Additional file 1: Table S5). The two sampled tachimenine dipsadines, *Thamnodynastes hypoconia* and *Tomodon dorsatus*, have the double substitution S164A/Y261F, and a predicted λ_max_ at ~545 nm [46]. The nocturnal *Oxyrhopus guibei* and the diurnal *Erythrolamprus poecilogyrus* have the amino acid substitution S164A and presumed λ_max_ at ~553 nm [46].

Based on previous studies on the spectral tuning of the rhodopsin RH1 in mammals and fish, we assumed that the amino acid replacements D83N, A292S and S299A would lead to downward shifts of the λ_max_ by 6, 10 and 2 nm, respectively [47, 48, 56, 59, 60]. The primarily nocturnal dipsadine colubrids *D. indica*, *S. mikanii*, *S. neuwidii,* and *O. guibei*, have the amino acids D83, A292 and S299, and for this combination, we predicted a λ_max_ at ~500 nm. The double substitution D83N/S299A was observed in the primarily nocturnal and fossorial *A. reticulatus*, with predicted λ_max_ at ~493 nm. All primarily diurnal species sampled in this study have the residues N83, S292, A299 and thus a predicted λ_max_ at ~483 nm (Additional file 1: Table S6).

The spectral sensitivity of the SWS1 in the UV was determined based on residue 86 [52–54]. The amino acid F86 was observed in all snakes, with exception of the diurnal *Chironius bicarinatus*, which has the substitution F86 V (Additional file 1: Table S7). In *Helicops modestus* residue 86 is heterozygous, with the amino acids valine and phenylalanine (Additional file 1: Table S7; Figure S2). For all species we presumed a spectral sensitivity in the UV, with λ_max_ at ~360 nm, with exception of *C. bicarinatus*, which we presumed to have a spectral shift toward the violet, and *H. modestus*, for which we predicted the possibility of two distinct photopigments, with different spectral absorption peaks, in the UV and in the violet.

### Retinal morphology

**Fig. 2:**
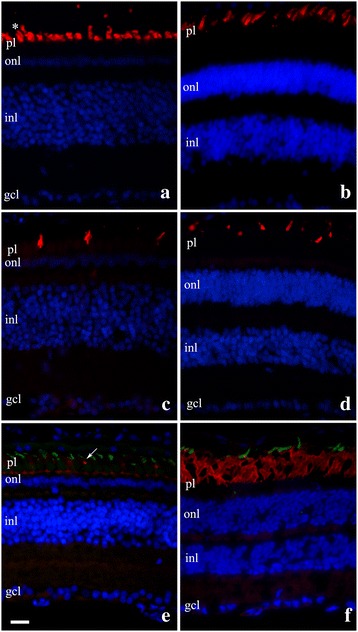
Retinal radial sections of the primarily diurnal *Tomodon dorsatus* (**a**, **c**, **e**) and the primarily nocturnal *Sibynomorphus neuwiedi* (**b**, **d**, **f**), labeled with anti-opsin antibodies and agglutinins. **a** outer segments of double cones (*) and large single cones and **b**, outer segments of large singles cones, labeled by the antibody AB5405, against L/M cones, revealed in red by CY3. **c** and **d**, outer segments of small single cones, labeled by the antibody AB5407, against UV/VS cones, revealed in red by CY3. **e** and **f**, double labeling by the agglutinins PNA and WGA: outer segments of cones labeled by PNA, reveled in green by FITC, and the outer segments of small single cones (arrow) (**e**) and rods (**f**), labeled by WGA, revealed in red by CY3. Cells *nuclei* labeled by DAPI (blue). pl, photoreceptor layer; onl, outer nuclear layer; inl, inner nuclear layer; gcl, ganglion cell layer. Scale bar = 20 μm

A retinal structure pattern was observed among species with distinct circadian activities. All primarily diurnal snakes had only one layer of nuclei in the outer nuclear layer (ONL) (Fig. 2). Four distinct cone types were identified in all primarily diurnal species analyzed: a large amount of large single cones and double cones, labeled by the antibody against L/M cones (LWS opsins); a small group of small single cones, labeled by the antibody against UV/VS cones (SWS1 opsin); and a small number of very small single cones, labeled by the agglutinin WGA, which typically labels rod outer segments in other vertebrates. An example of the diurnal retinal structure (*T. dorsatus*) is presented in Fig. 2. The retinas of primarily nocturnal snakes had a thick outer nuclear layer with many rows of photoreceptor nuclei (Fig. 2). A large number of rod outer segments was stained by WGA. Two distinct cone classes were identified, large single cones, labeled by the antibody against L/M cones (LWS opsins); and a small number of small single cones, labeled by the antibody against UV/VS cones (SWS1 opsin). No double cone was observed in the retinas of nocturnal colubrid species. An example of the nocturnal retinal structure (*S. neuwiedi*) is presented in Fig. 2.

## Discussion

The diversity of circadian activity patterns of snakes led to extreme adaptations of their retinal morphology to photopic or scotopic vision [3, 4, 6–9]. In our study, we confirmed with the agglutinin WGA, the presence of a small number of transmuted rods, characterized previously as small single cones [20–22], in retinas of diurnal colubrids. Our results are in agreement with Schott and colleagues’ [4] conclusion that a class of cones in at least some diurnal colubrids (*T. sirtalis*) possess the rhodopsin RH1 photopigment in the outer segment, and are in fact transmuted rods, which might contribute to the diurnal color vision [3, 4]. In nocturnal colubrids, we verified the presence of two groups of cones: large single cones with the LWS photopigment and small singles cones with the SWS1 photopigment. This is the first study, as far as we know, that uses imunohistochemistry to label distinct cone types in retinas of nocturnal caenophidian snakes. We were not able to identify double cones in the retinas of the primarily nocturnal colubrids analyzed although these photoreceptors were previously observed in retinas of some nocturnal colubrids [7, 9, 61]. The absence of double cones was also reported in retinas of the nocturnal brown tree snake *Boiga irregularis* [18].

The morphological differences between the retinas of primarily diurnal and primarily nocturnal colubrids sampled here are extreme, especially concerning the thickness of the ONL, with many rows of photoreceptor nuclei in nocturnal species, compared to only one row in diurnal colubrids. These anatomical observations are consistent with cell density estimates of photoreceptors and ganglion cells from wholemount retinas of diurnal and nocturnal colubrids [62, 63]. Diurnal snakes had much lower density of photoreceptors compared to nocturnal, but slightly higher density of ganglion cells [62, 63]. The estimated ratios of photoreceptors to ganglion cells in diurnal snakes were 1.2:1, while nocturnal snakes had a ratio of 10.7:1. Thus, the lower number of photoreceptors in diurnal species generates a lower convergence from cones to ganglion cells, which may improve the spatial resolution power, compared to nocturnal, with a high convergence from photoreceptors to ganglion cells. These differences in density [62, 63] are also suggested by examination of retinal sections, and indicate the potential for high sensitivity to light in nocturnal colubrids and low sensitivity in diurnal species, with the possibility of increasing the capacity of color discrimination [64], which still has to be proven by eletrophysiological and behavioral experiments.

Despite these notable morphological differences, Simões and colleagues [5] found that visual pigment genes in snakes are under high evolutionary constraint. In our study, we further demonstrate evidence for differential evolutionary constraints of the three opsin genes in primarily diurnal and primarily nocturnal colubrid lineages. Comparing our results obtained for the colubrids with those estimated for other snake families (see Table S1 from [5]), there are slightly lower functional constrains for the opsin genes *LWS* and *RH1* in diurnal and nocturnal colubrids (compare Table 2 with Table S12 from [5]). We estimated lower selective pressure for the *LWS* gene of diurnal colubrids (ω = 0.55). The ω values of the *SWS1* opsin gene for diurnal and nocturnal colubrids were very close to those estimated for a broader family sampling [5], indicating higher selective pressure on this gene.

The close similarity between the *SWS1* tree and species tree topology indicates the high conservation and selective pressure on this gene. The *SWS1* has been considered a good marker for inferring phylogenetic relationships among vertebrates at higher and lower taxonomic levels [65], and our results are in agreement with this. However, branch and branch-site models indicated that activity pattern might have influenced the magnitude of selection acting on the *SWS1*. The slightly lower ω value of nocturnal lineages (0.08) compared to diurnal lineages (0.13), indicates a stronger purifying selection to maintain the short wavelength sensitive photopigment in nocturnal snakes. In nocturnal mammals, distinct light environments have influenced the strength of selection acting on *SWS1* [66]. For instance, nocturnal lemurs from open canopy forests experience stronger purifying selection to maintain SWS cones and thus a dichromatic color vision, compared to species from closed canopy [66], where the intensity of short wavelength light is considerably lower.

Most snake species analyzed in this study have the amino acid F86 and were considered as UV sensitive. Determination of the residues responsible for the spectral shift of the SWS1 photopigment is somewhat controversial and might involve a more complex mechanism than those of the other opsin classes [47]. Nevertheless, there is consensus that residue 86 has an influence on the shift of ultraviolet sensitive (UVS) to violet sensitive (VS) visual pigments, and the presence of the amino acid F86 determines the UV sensitivity in various vertebrate lineages [1, 51, 52]. The sensitivity to UV light was described in diurnal and nocturnal caenophidian [2–4, 20] and henophidian snakes [1, 17, 67]. The ecological importance of UV sensitivity in terrestrial snakes has been associated with the ability to locate prey by viewing traces of rodents’ feces and urine that reflect UV light [68, 69] or the ability to locate mating partners by visualizing pheromone trails [70] that absorb UV light [71]. The F86 V amino acid substitution was found in the primarily diurnal colubrines *Ahaetulla nasuta*, *Chrysopelea ornata*, *Chironius carinatus*, *C. fuscus* [5] and *C. bicarinatus*. In the rodent *Cavia porcellus*, residue V86 seems to be responsible for the shift from UVS to VS [72]. Accordingly, studies on the ocular media transmission in different snake species [5] revealed that lens transmission in some primarily diurnal species, mainly in visual hunters such as *C. ornata* and *A. nasuta*, cuts off the UV wavelengths [5]. The finding of UV cutoffs might indicate that these snakes should have a long-wave shifted spectral peak of the SWS1 visual pigment [5], which have to be verified by behavioral, physiological or MSP analyses. In the semi-aquatic dipsadines *H. angulatus* [5] and *H. modestus*, site 86 is heterozygous, with both phenylalanine and valine (Additional file 1: Table S7; [5]). Simões and colleagues [5] speculated that this polymorphism might be responsible for the presence of two distinct visual pigments in the retina, with absorption peaks at the UV and violet, which may provide the potential for a trichromatic condition.

Conflict between *LWS* and *RH1* gene trees and the species tree might be explained by differences in selective pressure in particular groups, such as nocturnal and diurnal. Conflicts between gene trees and species phylogenies have been associated with divergent selection pressures and accelerated evolution in functional genes [73, 74], and was observed in the *RH1* gene trees of bats and rodents [74]. Our phylogenetic estimate for colubrid *RH1* differed from that of Simões and colleagues [5], with the colubrine species *A. nasuta* and *C. ornata* forming a highly supported branch outside a clade comprising most other Colubrinae, and *Hemorrhois hippocrepis* outside Dipsadinae (Fig. 1). The clade formed by primarily nocturnal species may indicate an evolutionary constraint in this group related to activity pattern. The activity pattern classification of *Lampropeltis* seems to be controversial, and some authors described primarily nocturnal activity [75–77]. Thus, the relationships of the two sampled *Lampropeltis* species’ *LWS* and *RH1* sequences might be associated with nocturnal activity and evolutionary adaptations of the *LWS* and *RH1* opsins in this group of snakes.

A discrepancy between predicted and measured λ_max_ of the LWS photopigment was observed for some species of snakes in which both genetic analyses and microspectrophotometry (MSP) have been performed [1, 4]. In our results, BEB analysis indicated several residues under positive selection (Table 3) and these are interesting targets for investigation by site-directed mutagenesis of the presence of other residues involved in spectral tuning.

The molecular evolutionary analysis indicates that the best intermediate branch model for the *RH1* gene was that with independent ω values for diurnal and nocturnal lineages (Table 3). Despite the overall low ω value (<1), which is evidence of purifying selection [36], it is worth noting that differential signatures of selection were detected along the diurnal and nocturnal branches for the *RH1* gene (Table 2), with stronger purifying selection in primarily diurnal snakes. Thus, results from both phylogenetic reconstruction and molecular evolutionary analysis, suggest that activity pattern is acting on the evolution of the rhodopsin *RH1* gene in diurnal and nocturnal colubrids.

Among the sites indicated as under positive selection in *RH1*, residues 83, 292, and 299 in the nocturnal lineage and residue 292 in the diurnal lineage are involved in the spectral shift of the RH1 photopigment [49, 50, 60] (Table 3; Additional file 1: Table S6). The substitution D83N is responsible for a blue-shift of 6 nm in the λ_max_ of the RH1 [60] and was observed in diurnal and fossorial caenophidian snakes (this study, [2, 4–6]) and in henophidian species [1] (Additional file 1: Table S6). All sampled primarily diurnal snakes had the amino acids N83/S292, which may generate a rhodopsin with λ_max_ at ~483 nm [60]. It was concluded in previous studies [4] that the group of small single cones with λ_max_ at 482 nm in the diurnal *T. sirtalis* expresses the rhodopsin gene *RH1*, and may contribute to the diurnal color vision. The expression of *RH1* in all diurnal snakes studied so far (with exception of *Macroprotodon brevis* [5]), with predicted λ_max_ at ~483 nm, and the presence of small cone-like photoreceptors labeled by specific agglutinin for rods, gives further support to this conclusion. The loss of the middle wavelength sensitive opsin RH2 in ancestral snakes, which is present in non-snake squamates [2], may be compensated for the gain of a cone expressing a blue-shifted rhodopsin [4], which along with the L/M and UV/VS cones may provide the potential for a trichromatic color vision [4]. The double substitution D83N/A292S occurred independently in different classes of aquatic and terrestrial vertebrates [51, 78–80] and seems to be an adaptation to different photic environments, such as a blue-green photic condition in deep water, but was never reported to be related to the circadian activity pattern, as observed in snakes.

Assuming that the rhodopsin is actually contributing to photopic color vision in diurnal snakes, one would expect that the RH1 photopigment underwent a reduction in sensitivity in this group. Rods and cones differ from each other in the temporal course of their response to light. Cones display faster photoresponses [14, 16], which is a consequence of the faster regeneration from 11-cis-retinal and the opsin and a rapid formation and decay of meta-II intermediate [81]. A rhodopsin containing N83 generates substantially larger amount of meta-II during the initial phase of the phototransduction, compared to those containing D83 [78]. Therefore, the substitution D83N found in diurnal and fossorial snakes may increase the sensitivity to light and may account for adaptation to a low-light environment. The presence of other residues that regulate the rates of regeneration and meta-II decay in diurnal species may be considered [81] and the sites indicated to be under positive selection in the diurnal lineage (Table 3) are a possible starting point for investigating these specific properties of the RH1 visual pigment.

## Conclusions

Our data indicate that the three visual pigment genes of colubrid snakes are under purifying selection, and that daily activity patterns have influenced the evolution of the rhodopsin *RH1* and the cone opsin *SWS1* gene. *RH1* and *LWS* gene trees recover clades characterized more by daily activity patterns than by known species relationships, possibly as a result of variation in selection pressures and accelerated evolution. For *SWS1*, the branch-model test results indicate a stronger signal of purifying selection in nocturnal lineages, which suggests a higher evolutionary pressure to maintain short wavelength sensitivity in low light conditions and the potential for a dichromatic color vision in primarily nocturnal snakes. For *RH1*, a stronger signal of purifying selection in primarily diurnal lineages is consistent with evolutionary pressure to maintain structure and function of the rhodopsin in this group. As suggested in previous studies [4, 6], our morphological analysis also indicate that the rhodopsin photopigment is expressed in cone-like photoreceptors and may contribute to diurnal color vision in primarily diurnal colubrids. We found differences in the predicted spectral tuning between primarily diurnal and primarily nocturnal snakes only for rhodopsin. Amino acid sites under positive selection are involved in RH1 photopigment spectral tuning, including residue 83, also involved in functional adaptation to dim-light vision. It is likely that other unidentified residues may be involved in promoting a faster photoresponse of the RH1 photopigment in diurnal colubrids, enabling its functional activity in daylight. Several sites indicated to be under positive selection are uncharacterized residues with regard to protein structure and function and represent good candidates for future studies.

## Additional files

Additional file 1: Tables S1-S7 and **Figures S1-S2.** Full legends are contained within the file. (PDF 1035 kb)

Additional file 2: Figure S3. Full legends are contained within the file. (PDF 226 kb)

## Abbreviations

BEB: Bayes Empirical Bayes
BI: Bayesian Inference
BIC: Bayesian information criterion
BP: Bayesian posterior probability
dN: Nonsynonymous substitutions
dS: Synonymous substitutions
L/M: Long/medium wavelengths
LRT: Likelihood ratio test
MCMC: Markov Chain Monte Carlo
ML: Maximum Likelihood
MSP: Microspectrophotometry
ONL: Outer nuclear layer
PNA: Peanut agglutinin
UV/VS: Ultra-violet/violet wavelengths
WGA: Wheat germ agglutinin
