# Supplementary material for: Daily activity patterns influence retinal morphology, signatures of selection, and spectral tuning of opsin genes in colubrid snakes
Source: BMC Evol Biol. 2017 Dec 11;17:249. doi: 10.1186/s12862-017-1110-0 (PMC5725783; doi:10.1186/s12862-017-1110-0)
Supplement: Supplementary file 1 — and Figures S1-S2. Full legends are contained within the file. (PDF 1035 kb) [file 12862_2017_1110_MOESM1_ESM.pdf]

Daily activity patterns influence retinal morphology, signatures of selection, and spectral tuning of opsin genes in colubrid snakes.

Hauzman E, Bonci DMO, Suárez-Villota EY; Neitz M, Ventura DF

Additional file 1 with Tables S1-S7 and Figures S1-S2.

Table S1. Primers designed and used in this study, to amplify the opsin genes *LWS*, *RH1* and *SWS1*, expressed in retinas of Colubridae snakes.

Primer	Opsin	Annealing Temperature	Nucleotide Sequence
SquaLWSFw	LWS	59.5	5' ATGACAGAGGCCTGGAATGT 3'
SquaLWSRv	LWS	60.0	5' CCTTGCTGGCATTCTCTTCT 3'
LWS DipsFw	LWS	62.0	5' CTTCGTCACCATCAAGTACAAGAAGT 3'
LWS DipsRv	LWS	62.0	5' GTCTTCTGAGAGCTCAGGTCAGACT 3'
LWS DegFw	LWS	62.4	5' GRYCCTTTTGAAGGYCCAAAYTAYC 3'
LWS DegRv	LWS	62.8	5' GTTGTAAATGGTKGCGCTTTTTG 3'
LWS TdorFw	LWS	61.6	5' TTTACTTACACCAACAGCAACAATACC 3'
LWS TdorRv	LWS	61.5	5' TTCATGAAGACATAGATAATTGGGTTG 3'
LWS LpoeFw	LWS	62.5	5' GTGTATTTGCTTACACCAATAGCAACA 3'
LWS LpoeRv	LWS	63.4	5' AAAATGTATATGGTCCCCAGCAGAC 3'
SquaRH1Fw	RH1	59.9	5' GCATTGTTCGGAGTCCATTT 3'
SquaRH1Rv	RH1	62.5	5' TTCTGCTGGCACCAAGACAG 3'
RH1SmikFw	RH1	65.8	5' TATTTGGCAGATCCATGGAAATATTCTG 3'
RH1SmikRv	RH1	66.1	5' GGGGAAACCTTACTTGTGGAGACTGTAG 3'
RH1OxyFw	RH1	64.5	5' GTCACCATCCAACACAAGAAACTCA 3'
RH1OxyRv	RH1	64.0	5' AAATCACTGGGTTGTAGATTGAAGAGC 3'
RH1Tdor1Fw	RH1	60.0	5' CACCACTACCATGTACACTTCCAT 3'
RH1Tdor1Rv	RH1	60.0	5' GTCATAAAGACAGGGCCAAAGT 3'
RH1Tdor2Fw	RH1	60.0	5' CAAGAAACTCAGAACACCTCTGAA 3'
RH1Tdor2Rv	RH1	60.0	5' CGGAACTGTTTGTTCATTACGATA 3'
SquaSWS1Fw	SWS1	55.6	5' CCTCTTTGAAAACATCTCCTC 3'
SquaSWS1Rv	SWS1	59.8	5' GTCCTCCTGTCAAGTCAGCC 3'
SWS1ArtfFw	SWS1	64.0	5' GACGGTCCCCAGTATCACATTG 3'
SWS1ArtfRv	SWS1	64.7	5' CTTGACAGGAGGACACTGAGGAAAC 3'
SWS1 TdorFw	SWS1	63.7	5' CTTTCCACTTCCAAACCATCTTCAT 3'
SWS1 TdorRv	SWS1	63.4	5' TTCTGAGAAGTCACATCAGAGTCGT 3'
SWS1 Tdor2Fw	SWS1	64.8	5' ATCCTCTTCGTCACCATCAAGTACAAG 3'
SWS1 Tdor2Rw	SWS1	64.1	5' ATGAAGCAGTAGATGATGGGGTTGT 3'
SWS1 DipsFw	SWS1	62.0	5' TACCACCAGGGAGAGTGTATTTGTGTA 3'
SWS1 DipsRv	SWS1	62.0	5' ACTTTCTTGCCAAAGAGCTGCATTAT 3'

Table S2. Species used as outgroups for phylogenetic analysis and respective GenBank accession numbers.

**Comon name**	**Species**	**GenBank acesison number**				
		**RH1**	**RH2**	**SWS1**	**SWS2**	**LWS/MWS**
Salamander	*Ambystoma tigrinum*	Q90245	-	-	AF038946	-
Worm lizard	*Amphisbaena alba*	KR336729	-	KR336720	-	KR336705
Worm lizard	*Amphisbaena infraorbitale*	KR336730	-	KR336719	-	KR336704
Lizard	*Anolis carolinensis*	NM001291387	AF134189	AH007736	-	P41592
Lizard	*Bachia flavescens*	KR336731	-	KR336715	-	KR336703
Cow	*Bos taurus*	NM001014890	-	NP776992	-	AAG49893
Goldfish	*Carassius auratus*	L11863	-	Q90309	-	L11867
Pigeon	*Columba livia*	-	AH007731	AH007798	-	-
Zebrafish	*Danio rerio*	NP571287	Q9W6A6	Q9W6A9	BAC24133	NM001002443
Chicken	*Gallus gallus*	P22328	P28683	P28684	P28682	P22329
Lizard	*Gekko gekko*	-	-	P35357	-	P35358
Lamprey	*Geotria australis*	AAR14682	AAR14683	AY366495	AAR14681	AY366491
Gorilla	*Gorilla gorilla*	XM004036292	-	XM004046176	-	-
Human	*Homo sapien*	NP00530	-	P03999	-	NM020061.5
Coelacanth	*Latimeria chalumnae*	-	AF131258	-	-	-
Lizard	*Melanoseps occidentalis*	KR336743	-	KR336718		KR336713
Mouse	*Mus musculus*	NP663358	-	P51491	-	O35599
Lungfish	*Neoceratodus forsteri*	EF526295	EF526296	EF526298	-	EF526297
Platypus	*Ornithorhynchus anatinus*	-	-	-	ABN43075	-
Day Gecko	*Phelsuma madagascariensis*	-	AF074044	AAD45183	-	AAD25917
Rat	*Rattus norvegicus*	NM033441	-	NM031015	-	NM053548
Fish	*Scleropages formosus*	XM018762271	-	XM018755948	-	XM018725556
Canary	*Serinus canaria*	XM009099359	-	AY227178	-	XM009100678
Boar	*Sus scrofa*	NM214221	-	NM214090	-	NM001011506
Zebra finch	*Taeniopygia guttata*	AF222329	NM00107669	AF222331	NM001076697	AF222333
Lizard	*Takydromus sexlineatus*	KR336727	-	KR336722	-	KR336707
Lizard	*Uta stansburiana*	DQ100323	DQ100324	DQ100325	DQ100326	DQ129869
Frog	*Xenopus laevis*	AAC42232	-	P51473	AAO38746	U90895
Frog	*Xenopus tropicalis*	NM00109733	-	NM001126076	-	NM001102861

Table S3. Colubridae species used for phylogenetic and evolutionary analysis and respective GenBank accession numbers. In bold, species sequenced in this study.

**Family**	**Subfamily**	**Species**	**GeneBank Acession Number**		
			**LWS**	**RH1**	**SWS1**
		Ahaetulla nasuta	KX237798	KX237852	KX237906
		Arizona elegans	KU323986	KU324006	KU323997
		Bogertophis subocularis	KX237822	KX237844	KX237920
		Boiga ceylonensis	KX237819	-	-
		Boiga forsteni	KX237818	KX237867	-
		Chironius carinatus	KX237795	KX237846	KX237914
		Chironius bicarinatus	**MG544928**	**MG544945**	**MG544962**
		Chironius fuscus	KX237794	KX237845	KX237913
		Chrysopelea ornata	KX237799	KX237851	KX237907
		Dasypeltis scabra	KX237821	KX237856	KX237908
		Elaphe climacophora	KX237817	KX237845	KX237917
		Hemorrhois hippocrepis	KX237911	KX237835	KX237911
	Colubrinae	*Lampropeltis californiae*	KX237825	KX237858	KX237921
		Lampropeltis floridana	KX237826	KX237857	KX237922
		Lycodon aulicus	KX237820	KX237875	KX237915
		Macroprotodon brevis	KX237815	-	KX237909
		Opheodrys aestivus	KX237797	KX237839	KX237912
		Orthriophis taeniurus	KX237816	KX237862	KX237916
		Pantherophis guttatus	KX237824	KX237863	KX237918
		Phyllorhynchus decurtatus	KU323985	-	KU323996
		Pituophis catenifer	KX237823	KX237854	KX237919
		Pseustes poecilonotus	KR336711	KR336741	KR336725
		Spalerosophis diadema	KX237814	KX237853	KX237910
		Spilotes pullatus	**MG544940**	**MG544957**	**MG544974**
		Telescopus fallax	KU323984	KU324005	KU323995
		Atractus flammigerus	KR336712	KR336740	KR336726
Colubridae		*Atractus badius*	KX237809	KX237842	KX237902
		Atractus reticulatus	**MG544927**	**MG544944**	**MG544961**
		Dipsas indica	KX237813	KX237849	KX237904
		Dipsas catesbyi	KX237812	KX237848	KX237905
		Dipsas petersi	**MG544929**	**MG544946**	**MG544963**
		Echinanthera cephalostriata	**MG544930**	**MG544947**	**MG544964**
		Echinanthera undulata	**MG544931**	**MG544948**	**MG544965**
		Erythrolamprus aesculapii	**MG544932**	**MG544949**	**MG544966**
		Erythrolamprus miliaris	**MG544933**	**MG544950**	**MG544967**
		Erythrolamprus poecilogyrus	**MG544934**	**MG544951**	**MG544968**
		Erythrolamprus reginae	KX237800	KX237855	KX237894
		Helicops angulatus	KX237806	KX237836	KX237895
		Helicops modestus	**MG544935**	**MG544952**	**MG544969**
	Dipsadinae	*Heterodon nasicus*	KX237793	KX237850	KX237893
		Hypsiglena jani	KU323988	KU324007	KU323998
		Imantodes lentiferus	KX237807	KX237841	KX237900
		Leptodeira annulata	KX237808	KX237840	KX237901
		Oxyrhopus guibei	**MG544936**	**MG544953**	**MG544970**
		Oxyrhopus melanogenys	KX237804	KX237838	KX237899
		Philodryas patagoniensis	**MG544937**	**MG544954**	**MG544971**
		Pseudoboa coronata	KX237803	KX237837	KX237898
		Sibon nebulatus	KX237811	KX237843	KX237902
		Sibynomorphus mikanii	**MG544938**	**MG544955**	**MG544972**
		Sibynomorphus neuwiedi	**MG544939**	**MG544956**	**MG544973**
		Taeniophallus persimilis	**MG544941**	**MG544958**	**MG544975**
		Thamnodynaestes hypoconia	**MG544942**	**MG544959**	**MG544976**
		Tomodon dorsatus	**MG544943**	**MG544960**	**MG544977**
		Xenopholis scalaris	KX237810	KX237834	KX237897
Xenopeltidae		*Xenopeltis unicolor*	FJ497235	FJ49723	FJ497234
Pythonidae		*Python regius*	FJ4977238	FJ497236	FJ4977237

Table S4. Summary of phylogenetic evolutionary branch-site and site models parameters for *LWS*, *RH1* and *SWS1* opsin genes.

Opsins	Model	Ln L	P	Parameter Estimates
	Branch-Site Models			
	Null Model	-11,370.8	150	$\omega = 1.00$
	Model A (diurnal)	-11,347.9	152	$\omega_0 = 0.05$ ($p_0 = 0.798$), $\omega_1 = 1.0$ ($p_1 = 0.18$); $\omega_2 = 34.3$ ($p_2 + p_3 = 0.03$)
	Model A (nocturnal)	-11,320.3	152	$\omega_0 = 0.05$ ($p_0 = 0.77$), $\omega_1 = 1.0$ ($p_1 = 0.17$); $\omega_2 = 14.8$ ($p_2 + p_3 = 0.06$)
	Site Models			
**LWS**	Model 0	-4,598.5	101	$\omega_0 = 0.49$
	Model 3	-4,235.5	105	$\omega_0 = 0.03$ ($p_0 = 0.82$), $\omega_1 = 1.8$ ($p_1 = 0.14$); $\omega_2 = 6.08$ ($p_2 = 0.04$)
	Model 1a	-4,316.4	102	$\omega_0 = 0.014$ ($p_0 = 0.798$), $\omega_1 = 1.0$ ($p_1 = 0.202$)
	Model 2a	-4,238.3	104	$\omega_0 = 0.017$ ($p_0 = 0.78$), $\omega_1 = 1.0$ ($p_1 = 0.13$); $\omega_2 = 4.19$ ($p_2 + p_3 = 0.08$)
	Model 7	-4,324.8	102	$p = 0.0158$, $q = 0.0987$
	Model 8	-4,238.7	104	$p_0 = 0.9095$, $p = 0.038$, $q = 0.24$ ($p_1 = 0.090$, $\omega = 3.989$)
	Branch-Site Models			
	Null Model	-10,943.6	146	$\omega = 1.00$
	Model A (diurnal)	-10,939.4	148	$\omega_0 = 0.055$ ($p_0 = 0.86$), $\omega_1 = 1.0$ ($p_1 = 0.14$); $\omega_2 = 4.36$ ($p_2 + p_3 = 0.007$)
	Model A (nocturnal)	-10,918.9	148	$\omega_0 = 0.057$ ($p_0 = 0.86$), $\omega_1 = 1.0$ ($p_1 = 0.12$); $\omega_2 = 11.4$ ($p_2 + p_3 = 0.02$)
	Site Models			
**RH1**	Model 0	-4,692.8	97	$\omega_0 = 0.288$
	Model 3	-4,479.1	101	$\omega_0 = 0.002$ ($p_0 = 0.67$), $\omega_1 = 0.43$ ($p_1 = 0.23$); $\omega_2 = 2.02$ ($p_2 = 0.10$)
	Model 1a	-4,497.7	98	$\omega_0 = 0.022$ ($p_0 = 0.77$), $\omega_1 = 1.0$ ($p_1 = 0.23$)
	Model 2a	-4,483.4	100	$\omega_0 = 0.024$ ($p_0 = 0.77$), $\omega_1 = 1.0$ ($p_1 = 0.18$); $\omega_2 = 2.7$ ($p_2 + p_3 = 0.048$)
	Model 7	-4,496.8	98	$p = 0.018$, $q = 0.059$
	Model 8	-4,479.5	100	$p_0 = 0.926$, $p = 0.098$, $q = 0.555$ ($p_1 = 0.074$, $\omega = 2.26$)
	Branch-Site Models			
	Null Model	-14,248.6	154	$\omega = 1.00$
	Model A (diurnal)	-14,225.3	156	$\omega_0 = 0.05$ ($p_0 = 0.84$), $\omega_1 = 1.0$ ($p_1 = 0.077$); $\omega_2 = 1.23$ ($p_2 + p_3 = 0.078$)
	Model A (nocturnal)	-14,237.9	156	$\omega_0 = 0.05$ ($p_0 = 0.87$), $\omega_1 = 1.0$ ($p_1 = 0.08$); $\omega_2 = 1.7$ ($p_2 + p_3 = 0.046$)
	Site Models			
**SWS1**	Model 0	-5,784.0	99	$\omega_0 = 0.1$
	Model 3	-5,565.2	103	$\omega_0 = 0.00$ ($p_0 = 0.64$), $\omega_1 = 0.17$ ($p_1 = 0.25$); $\omega_2 = 0.73$ ($p_2 = 0.11$)
	Model 1a	-5,618.6	100	$\omega_0 = 0.039$ ($p_0 = 0.86$), $\omega_1 = 1.0$ ($p_1 = 0.14$)
	Model 2a	-5,618.6	102	$\omega_0 = 0.039$ ($p_0 = 0.86$), $\omega_1 = 1.0$ ($p_1 = 0.1$); $\omega_2 = 1.0$ ($p_2 + p_3 = 0.039$)
	Model 7	-5,568.3	100	$p = 0.13$, $q = 0.899$
	Model 8	-5,568.3	102	$p_0 = 0.998$, $p = 0.13$, $q = 0.92$ ($p_1 = 0.002$, $\omega = 1.04$)

p, number of parameters; p_0, p_1, p_2, proportion of sites in different omega classes; l, likelihood.

Table S5. Known amino acid spectral tuning sites of the *LWS* opsin gene and predicted peak absorbance (λ_{max}) [1]. Sites numbered based on the bovine rhodopsin sequence. All λ_{max} values are estimates predicted based on amino acid sequences. Predominant daily activity patterns stablished based on [2, 3].

Species	164	181	261	269	292	λ_{max}
**Primarily Nocturnal**						
Atractus reticulatus	A	H	Y	A	A	~537
Dipsas petersi	A	H	Y	A	A	~537
Sibynomorphus mikanii	A	H	Y	A	A	~537
Sibynomorphus neuwiedi	A	H	Y	A	A	~537
Oxyrhopus guibei	A	H	Y	T	A	~553
**Primarily Diurnal**						
Chironius bicarinatus	S	H	Y	T	A	~560
Echinanthera cephalostriata	S	H	Y	T	A	~560
Echinantera undulata	S	H	Y	T	A	~560
Erythrolamprus aesculapii	S	H	Y	T	A	~560
Erythrolamprus miliaris	S	H	Y	T	A	~560
Erythrolamprus poecilogyrus	A	H	Y	T	A	~553
Helicops modestus	S	H	Y	T	A	~560
Philodryas patagoniensis	S	H	Y	T	A	~560
Spilotes pullatus	S	H	Y	T	A	~560
Taeniophallus persimilis	S	H	Y	T	A	~560
Thamnodynastes hypoconia	A	H	F	T	A	~543
Tomodon dorsatus	A	H	F	T	A	~545

Table S6. Known amino acid spectral tuning sites of the RH1 rhodopsin gene and predicted peak absorbance (λ_{max}) [4, 5, 6]. Sites numbered based on the bovine rhodopsin sequence. All λ_{max} values are estimates predicted based on amino acid sequences. Predominant daily activity patterns stablished based on [2, 3].

Species	83	96	102	122	183	194	195	253	261	289	292	299	317	λ_{max}
**Primarily Nocturnal**														
Atractus reticulatus	**N**	Y	Y	E	M	P	T	M	F	T	**A**	**A**	M	~493
Dipsas petersi	**D**	Y	Y	E	M	P	S	M	F	T	**A**	**S**	M	~500
Sibynomorphus mikanii	**D**	Y	Y	E	M	P	S	M	F	T	**A**	**S**	M	~500
Sibynomorphus neuwiedi	**D**	Y	Y	E	M	P	S	M	F	T	**A**	**S**	M	~500
Oxyrhopus guibei	**D**	Y	Y	E	M	P	S	M	F	T	**A**	**S**	M	~500
**Primarily Diurnal**														
Chironius bicarinatus	**N**	Y	Y	E	M	P	T	M	F	T	**S**	**A**	M	~483
Echinantera cephalostriata	**N**	Y	Y	E	M	P	T	M	F	T	**S**	**A**	M	~483
Echinantera undulata	**N**	Y	Y	E	M	P	T	M	F	T	**S**	**A**	M	~483
Erythrolamprus aesculapii	**N**	Y	Y	E	M	P	T	M	F	T	**S**	**A**	M	~483
Erythrolamprus miliaris	**N**	Y	Y	E	M	P	T	M	F	T	**S**	**A**	M	~483
Erythrolamprus poecilogyrus	**N**	Y	Y	E	M	P	T	M	F	T	**S**	**A**	M	~483
Helicops modestus	**N**	Y	Y	E	M	P	T	M	F	T	**S**	**A**	M	~483
Philodryas patagoniensis	**N**	Y	Y	E	M	P	T	M	F	T	**S**	**A**	M	~483
Spilotes pullatus	**N**	Y	Y	E	M	P	T	M	F	T	**S**	**A**	M	~483
Taeniophallus persimilis	**N**	Y	Y	E	M	P	T	M	F	T	**S**	**A**	M	~483
Thamnodynates hypoconia	**N**	Y	Y	E	M	P	T	M	F	T	**S**	**A**	M	~483
Tomodon dorsatus	**N**	Y	Y	E	M	P	T	M	F	T	**S**	**A**	M	~483

Table S7. Known amino acid spectral tuning sites of the SWS1 [7] and predicted peak absorbance (λ_{max}). Site numbered based on bovine rhodopsin. Asterisk indicates amino acids with stronger effects on spectral tuning [8, 9, 10]. All λ_{max} values are estimates predicted based on amino acid sequences (for a review see [11]). Predominant daily activity patterns stablished based on [2, 3].

Species	46	49	52	86*	90	93*	97	113*	114	116	118	265	λ_{max}
**Primarily Nocturnal**													
Atractus reticulatus	L	F	T	F	A	T	S	E	A	L	T	Y	UV
Dipsas petersi	L	F	T	F	A	T	S	E	A	L	T	Y	UV
Sibynomorphus mikanii	L	F	T	F	A	T	S	E	A	L	T	Y	UV
Sibynomorphus neuwiedi	L	F	T	F	A	T	S	E	A	L	T	Y	UV
Oxyrhopus guibei	L	F	T	F	A	V	S	E	A	L	T	Y	UV
**Primarily Diurnal**													
Chironius bicarinatus	L	F	T	V	A	V	S	E	A	L	T	Y	?
Echinantera cephalostriata	L	F	T	F	A	V	S	E	A	L	T	Y	UV
Echinantera undulata	L	F	T	F	A	V	S	E	A	L	T	Y	UV
Erythrolamprus aesculapii	L	F	T	F	A	T	S	E	A	L	T	Y	UV
Erythrolamprus miliaris	L	F	T	F	A	T	S	E	A	L	T	Y	UV
Erythrolamprus poecilogyrus	L	F	T	F	A	T	S	E	A	L	T	Y	UV
Helicops modestus	L	F	T	F/V	A	T/V	S	E	A	L	T	Y	UV/?
Philodryas patagoniensis	L	F	T	F	A	T	S	E	A	L	T	Y	UV
Spilotes pullatus	L	F	T	F	A	V	S	E	A	L	T	Y	UV
Taeniophallus persimilis	L	F	T	F	A	V	S	E	A	L	T	Y	UV
Thamnodynates hypoconia	L	F	T	F	A	T	S	E	A	L	T	Y	UV
Tomodon dorsatus	L	F	T	F	A	T	S	E	A	L	T	Y	UV

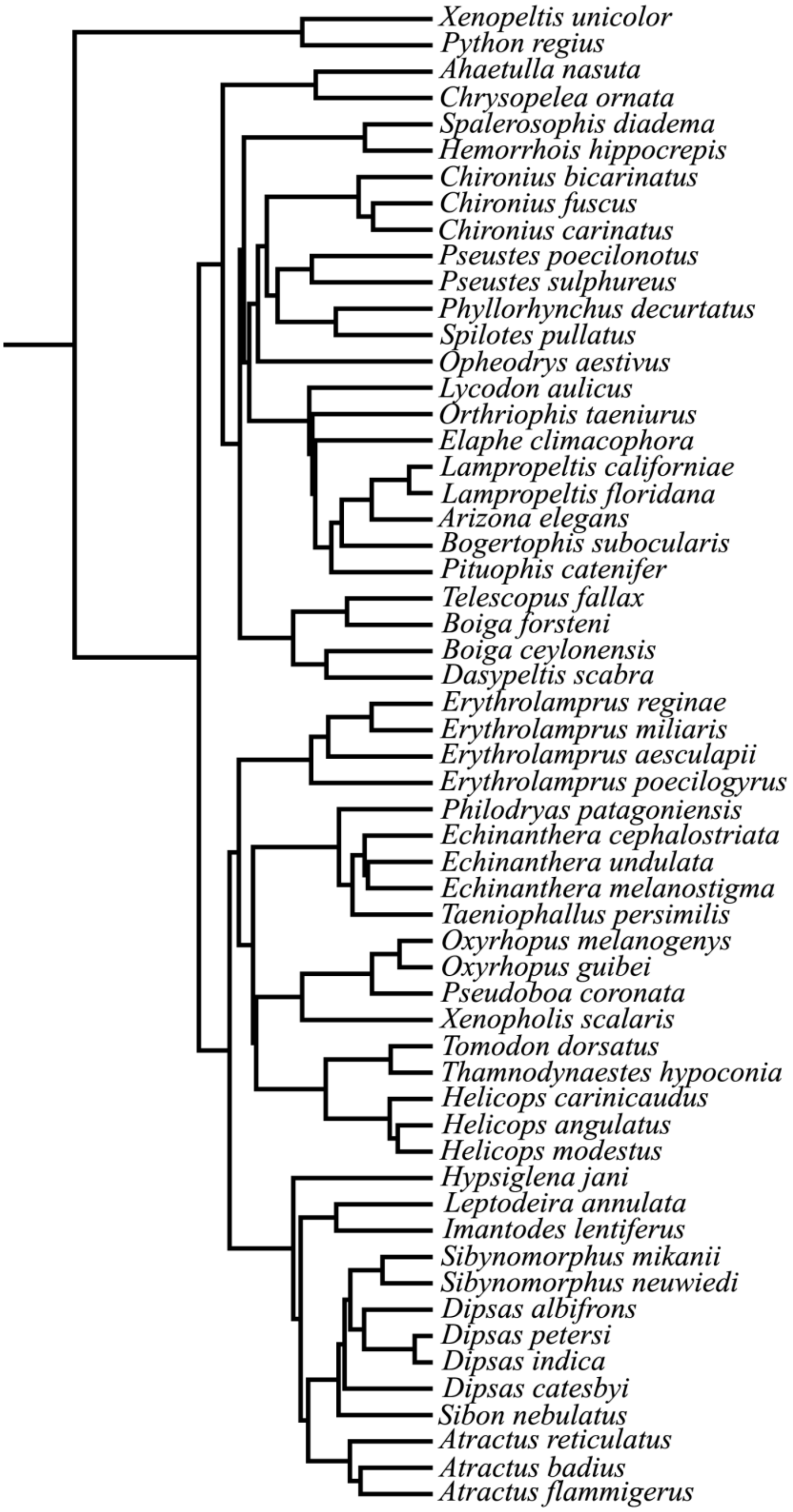

Figure S1. Species phylogenetic tree used for Codeml analysis.

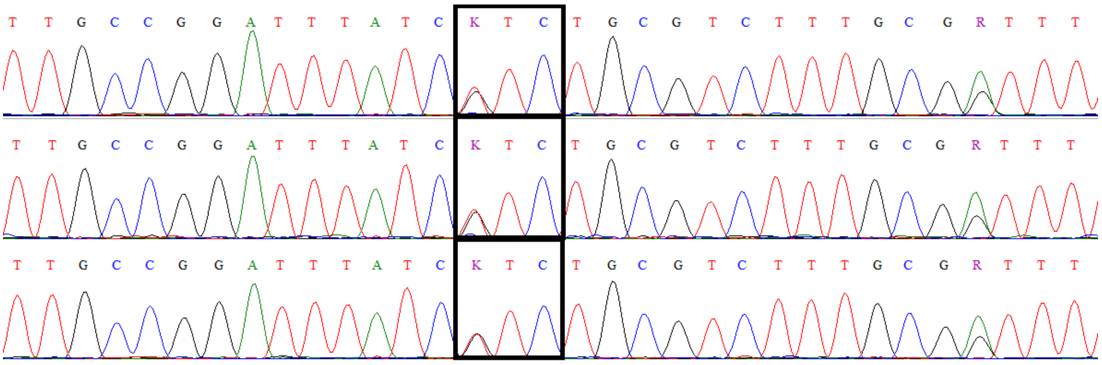

Figure S2. Chromatograms of the same region of the *SWS1* opsin gene of *Helicops modestus*, amplified with different primer pairs. The black boxes indicate the heterozygous site, with codons TTC and GTC, responsible for the transduction of the amino acids phenylalanine and valine at residue 86.

REFERENCES

1. Yokoyama S, Radlwimmer FB. The molecular genetics of red and green color vision in vertebrates. Genetics. 2001;158:1697–1710.
2. Torello-Viera NF, Marques OAV. Daily Activity of Neotropical Dipsadids Snakes. S Am J Herpetol. 2017;12(2):128-135.
3. Marques OAV, Eterovic A, Sazima I. Serpentes da Mata Atlântica: guia ilustrado para a Serra do Mar. Holos. 2001.
4. Hunt DM, Dulai KS, Partridge JC, Cottrill P, Bowmaker JK. The molecular basis for spectral tuning of rod visual pigments in deep-sea fish. J Exp Biol. 2001;204:3333–44.
5. Levenson DH, Ponganis PJ, Crognale MA, Deegan JF, Dizon A, Jacobs GH. Visual pigments of marine carnivores: pinnipeds, polar bear, and sea otter. J Comp Physiol A. 2006;192:833–43.
6. Yokoyama S, Tada T, Zhang H, Britt L. Elucidation of phenotypic adaptations: Molecular analyses of dim-light vision proteins in vertebrates. P Natl Acad Sci USA. 2008;105:13480–85.
7. Yokoyama S, Starmer WT, Takahashi Y, Tada T. Tertiary structure and spectral tuning of UV and violet pigments in vertebrates. Gene. 2006;365:95–103.
8. Babu KR, Dukkipati A, Birge RR, Knox BE. Regulation of phototransduction in short-wavelength cone visual pigments via the retinylidene schiff base counterion. Biochemistry. 2001;40:13760–66.
9. Cowing JA, Poopalasundaram S, Wilkie SE, Robinson PR, Bowmaker JK, Hunt DM. The molecular mechanism for the spectral shifts between vertebrate ultraviolet- and violet-sensitive cone visual pigments. Biochem J. 2002;367:129–35.
10. Fasick JI, Applebury ML, Oprian DD. Spectral tuning in the mammalian short-wavelength sensitive cone pigments. Biochemistry. 2002;41:6860–965.
11. Yokoyama S. Evolution of dim-light and color vision pigments. Annu Rev Genom Hum Genetics. 2008;9:259–82.
