# Supplementary material for: Daily activity patterns influence retinal morphology, signatures of selection, and spectral tuning of opsin genes in colubrid snakes
Source: BMC Evol Biol. 2017 Dec 11;17:249. doi: 10.1186/s12862-017-1110-0 (PMC5725783; doi:10.1186/s12862-017-1110-0)
Supplement: Supplementary file 2 — Full legends are contained within the file. (PDF 226 kb) [file 12862_2017_1110_MOESM2_ESM.pdf]

Additional File 2: Figure S3. 1. Nucleotide alignment of the visual pigments RH1, LWS and SWS1 expressed in snakes retinas and the bovine rhodopsin RH1.

```
                                    1        10         20         30         40         50         60         70         80         90         100        110        120
                                    |         |          |          |          |          |          |          |          |          |          |          |          |
Bos_taurus_Rh1                      ---------------------------------------------------------------------------------------ATGAACGGGACCGAGGGCCCAAACTTCTACGTG
Atractus_reticulatus_Rh1            -----------------------------------------------------------------------------------------------------------------------C
Chironius_bicarinatus_Rh1           ------------------------------------------------------------------------------------------------------------------------
Dipsas_petersi_Rh1                  ------------------------------------------------------------------------------------------------------------------------
Echinantera_cephalostriata_Rh1      ------------------------------------------------------------------------------------------------------------------------
Echinantera_undulata_Rh1            ------------------------------------------------------------------------------------------------------------------------
Erythrolamprus_aesculapii_Rh1       --------------------------------------------------------------------------------------------------------------------TGTC
Erythrolamprus_miliaris_Rh1         ---------------------------------------------------------------------------------------------------------------------GTC
Erythrolamprus_poecilogyrus_Rh1     ------------------------------------------------------------------------------------------------------------------------
Helicops_modestus_Rh1               ------------------------------------------------------------------------------------------------------------------------
Oxyrhopus_guibei_Rh1                ------------------------------------------------------------------------------------------------------------------------
Philodryas_patagoniensis_Rh1        ------------------------------------------------------------------------------------------------------------------------
Python_regius_Rh1                   ------------------------------------------------------------------------------------------------------------AATTTCTACGTC
Sibynomorphus_mikanii_Rh1           ------------------------------------------------------------------------------------------------------------------------
Sibynomorphus_neuwiedi_Rh1          ------------------------------------------------------------------------------------------------------------------------
Spilotes_pullatus_Rh1               ------------------------------------------------------------------------------------------------------------------------
Taeniophallus_persimilis_Rh1        ------------------------------------------------------------------------------------------------------------------------
Thamnodynastes_strigatus_Rh1        ------------------------------------------------------------------------------------------------------------------------
Tomodon_dorsatus_Rh1                ------------------------------------------------------------------------------------------------------------------------
Xenopeltis_unicolor_Rh1             ---------------------------------------------------------------------------------------ATGAATGGAACAGAAGGGCTTAATTTCTATGTC
Atractus_reticulatus_LWS            ----------------------------------TGACAGAGGCCTGGAATGTGGCAGTATTTGCTGCTCGGAGGCGCAACAAT---------GACGAAGATACCACCAGGGAGAGTATA
Chironius_bicarinatus_LWS           ---------------------------------ATGACAGAGGGCTGGAATGTGGCAGTATTTGCTGCTCGGCGGCATCACAAT---------GATGAAGATACCACTCAGGACAGTTTA
Dipsas_petersi_LWS                  -------------------------------------------GCTGGAATGTGGCAGTATTTGCTGCTCGCCGGCGTAACAAT---------GACGAAGATACCACCAGGGAGAGTGTA
Echinantera_cephalostriata_LWS      --------------------------------------AGAGGCCTGGAATGTGGCAGTATTTGCTGCTCGGCGGCGCAACGATGAC------GACGAAGATACCACCAAGGACAGTATA
Echinantera_undulata_LWS            -------------------------------------------------ATGTGGCAGTATTTGCTGCTCGGCGGCGCAACGATGAC------GACGAAGATACCACCAAGGACAGTATA
Erythrolamprus_aesculapii_LWS       ------------------------------------------------------------------------------------------------------------------------
Erythrolamprus_miliaris_LWS         --------------------------------------------CTGGAATGTGGCAGTATTTGCTGCTCGTCGACGCAGTGATGATGAT---GATGAAGATACCACCAGGGACAGTATA
Erythrolamprus_poecilogyrus_LWS     -------------------------------------------------------------------------------------------ATGATGAAGATACCACCAGGGACAGTGTA
Helicops_modestus_LWS               --------------------------------------------CTGGAATGTGGCAGTATTTGCTGCTCGGCGGCGCAACAATGAC------GATGAAGATACCACCAGGGACAGTGTA
Oxyrhopus_guibei_LWS                --------------------------------------------CTGGAATGTGGCAGTATTTGCTGCTCGGCGGCGCAACGATGAC------GACGAAGATACCACCAGGGACAGTGTA
Philodryas_patagoniensis_LWS        -------------------------------------------GCTGGAATGTGGCAGTATTTGCTGCTCGGCGGCGCAACGATGACGACGATGACGAAGATACCACCAGGGACAGTATA
Python_regius_LWS                   ---------------------GTAGCAAAGGCCATGACAGAGGCCTGGAATGTGGCAGTATTTGCTGCCAGGCGGCGCAAC------------GATGATGATACCACTAGAGAAAGTGTA
Sibynomorphus_mikanii_LWS           --------------------------------------------CTGGAATGTGGCAGTATTTGCTGCTCGGCGACGTAACAAT---------GACGAAGATACCACCAGGGAGAGTATA
Sibynomorphus_neuwiedii_LWS         -------------------------------------------GCTGGAATGTGGCAGTATTTGCTGCTCGGCGGCGTAACAAT---------GATGAAGATACCACCAGGGAGAGTGTA
Spilotes_pullatus_LWS               ------------------------------------------------------------TTTGCTGCTCGGCGGCACCACGAT---------GATGAAGATACCACTAAGGACAGTTTA
Taeniophallus_persimilis_LWS        --------------------------------------AGAGGCCTGGAATGTGGCAGTATTTGCTGCTCGGCGGCGCAACGAT---------GATGAAGATACCACCAAGGACAGTCTA
Thamnodynastes_strigatus_LWS        -----------------------------------GACAGAGACCTGGAATGTGCCAGTATTTGCTGCTCGGCGGCGCAATGATGAC------GATGAAGATACCACCAGGGACAGTTTA
Tomodon_dorsatus_LWS                -------------------------------------------GCTGGAATGTGCCAGTATTTGCTGCTCGGCGGCGCAATGAYGAC------GATGAAGATACCACCAGGGACAGTATA
Xenopeltis_unicolor_LWS             ATGCAAACAAGGGGGAAAGAAGTAGCAAAGGCCATGACAGAGGCCTGGAATGTGGCAATATTTGCTGCTAGACGACGCAACAAT---------GATGATGATACCACTAGAGAAAGTGTA
Atractus_reticulatus_SWS1           ------------------------------------------------------------------------------------------------------------------------
Chironius_bicarinatus_SWS1          ------------------------------------------------------------------------------------------------------------------------
Dipsas_petersi_SWS1                 ------------------------------------------------------------------------------------------------------------------------
Echinantera_cephalostriata_SWS1     ------------------------------------------------------------------------------------------------------------------------
Echinantera_undulata_SWS1           ------------------------------------------------------------------------------------------------------------------------
Erythrolamprus_aesculapii_SWS1      ------------------------------------------------------------------------------------------------------------------------
Erythrolamprus_miliaris_SWS1        ------------------------------------------------------------------------------------------------------------------------
Erythrolamprus_poecilogyrus_SWS1    ------------------------------------------------------------------------------------------------------------------------
Helicops_modestus_SWS1              ------------------------------------------------------------------------------------------------------------------------
Oxyrhopus_guibei_SWS1               ------------------------------------------------------------------------------------------------------------------------
Philodryas_patagoniensis_SWS1       ------------------------------------------------------------------------------------------------------------------------
Python_regius_SWS1                  ------------------------------------------------------------------------------------------------------------------------
Sibynomorphus_mikanii_SWS1          ------------------------------------------------------------------------------------------------------------------------
Sibynomorphus_neuwiedii_SWS1        ------------------------------------------------------------------------------------------------------------------------
Spilotes_pullatus_SWS1              ------------------------------------------------------------------------------------------------------------------------
Taeniophallus_persimilis_SWS1       ------------------------------------------------------------------------------------------------------------------------
Thamnodynastes_strigatus_SWS1       ------------------------------------------------------------------------------------------------------------------------
Tomodon_dorsatus_SWS1               ------------------------------------------------------------------------------------------------------------------------
Xenopeltis_unicolor_SWS1            ------------------------------------------------------------------------------------------------------ATGTCCGGAGAAGAGGAC
```

```
                                   121       130       140       150       160       170       180       190       200       210       230       240       250
                                     |         |         |         |         |         |         |         |         |         |         |         |         |
                 Bos_taurus_Rh1    CCTTTCTCCAACAAGACGGGCGTGGTGCGCAGCCCCTTCGAGGCCCCGCAGTACTACCTGGCGGAGCCATGGCAGTTCTCCATGCTGGCCGCCTACATGTTCCTGCTGATCATGCTTGGC
      Atractus_reticulatus_Rh1    CCTATGTCAAACAAGACTGGCATTGTTCGGAGTCCATATGAATATCCCCAATATTATTTGGCAGATCCATGGAAATATTCTGCCTTGGCTGCATACATGTTTCTTCTGATTCTGCTTGGA
      Chironius_bicarinatus_Rh1    -----------------------TGTTCGGAGTCCATTTGAGTATCCTCAATATTATTTGGCAGATCCATGGAAATATTCTGCCTTGGCTGCGTACATGTTTCTTCTGATTCTGCTTGGT
            Dipsas_petersi_Rh1    ----------ACAAGACTGGCGTTGTTCGGAGTCCATATGAGTATCCCCAATATTATTTGGCAGATCCATGGAAATATTCTGCCTTGGCTGCATACATGTTTCTTCTGATTCTGCTTGGA
Echinantera_cephalostriata_Rh1    ---------AACAAGACTGGCATTGTTCGGAGTCCATATGAGTATCCCCAATATTATTTGGCAGATCCTTGGAAATATTCTGCCTTGGCTGCATACATGTTTCTTCTGATTCTGCTTGGA
      Echinantera_undulata_Rh1    --------------------------------TCCATTTGAGTATCCCCAATATTATTTGGCAGATCCTTGGAAATATTCTGCCTTGGCTGCATACATGTTTCTTCTGATTCTGCTTGGA
   Erythrolamprus_aesculapii_Rh1    CCTATGTCAAACAAGACTGGCATTGTTCGGAGTCCATATGAGTATCCCCAATATTATTTGGCAGATCCATGGAAATATTCTGCCTTGGCTGCATACATGTTTCTTCTGATTCTGCTTGGA
    Erythrolamprus_miliaris_Rh1    CCTATGTCAAACAAGACTGGCATTGTTCGGAGTCCATATGAGTATCCCCAATATTATTTGGCAGATCCATGGAAATATTCTGCCTTGGCTGCATACATGTTTCTTCTGATTCTGCTTGGA
 Erythrolamprus_poecilogyrus_Rh1    ------------------------------------------------------------------------------------------------------------------------
         Helicops_modestus_Rh1    ---------AACAAGACTGGCGTTGTTCGGAGTCCATATGAGTATCCCCAATATTATTTAGCAGATCCATGGAAATATTCTGCCTTGGCTGCATACATGTTTCTTCTGATTCTGCTTGGA
          Oxyrhopus_guibei_Rh1    -----------------------TGTTCGGAGTCCATTTGAGTATCCCCAATATTATTTGGCAGATCCATGGAAATATTCTGCCTTGGCTGCATACATGTTTTTGCTGATTCTGCTTGGA
   Philodryas_patagoniensis_Rh1    -------------------------------------------------------------------------------------------------TGTTTCTTCTGATTCTGCTTGGA
              Python_regius_Rh1    CCTATGTCAAACAAGACTGGTATTGTTCGGAGTCCATTTGAGTATCCCCAATATTATTTGGCAGAACCATGGAAATATTCTGCCCTGGGTGCATACATGTTTCTTCTGATTCTGCTTGGA
     Sibynomorphus_mikanii_Rh1    -----------------------------------------------------------------ACCATGGAAATATTCTGCCTTGGCTGCATACATGTTTCTTCTGATTCTGCTTGGA
    Sibynomorphus_neuwiedi_Rh1    ------------------------------------------------------------------CCATGGAAATATTCTGCCTTGGCTGCATACATGTTTCTTCTGATTCTGCTTGGA
         Spilotes_pullatus_Rh1    -------------------------------------------ATTTGAGTTATTATTTGGCAGATCCATGGAAATATTCTGCCTTGGCTGCGTACATGTTTCTTCTGATTCTGCTTGGT
   Taeniophallus_persimilis_Rh1    ---------AACAAGACTGGCATTGTTCGGAGTCCATATGAGTATCCCCAATATTATTTGGCAGATCCTTGGAAATATTCTGCCTTGGCTGCATACATGTTTCTTCTGATTCTGCTTGGA
   Thamnodynastes_strigatus_Rh1    -------------------------------------------ATCCCCAATATTATTTGGCAGATCCATGGAAATATTCTGCCTTGGCTGCATACATGTTTCTTCTGATTCTGCTTGGA
          Tomodon_dorsatus_Rh1    ----------------------TTGTTCGGAGTCCATTTGAGTATCCCCAATATTATTTGGCAGATCCATGGAAATATTCTGCCTTGGCTGCATACATGTTTCTTCTGATTCTGCTTGGA
       Xenopeltis_unicolor_Rh1    CCTATGTCAAACAAGACTGGCATTGTTCGGAGTCCATTTGAGTATCCCCAATATTATTTGGCAGATCCATGGAAATATTCTGCTCTGGCTGCATACATGTTTCTTCTGATTCTGCTTGGA
      Atractus_reticulatus_LWS    TTTGTGTACACCAACAGCAACAATACCCGTGGCCCTTTTGAAGGTCCAAACTATCATATTGCACCACGATGGGTCTACAACTTAACTTCACTCTGGATGGTCTTTGTTGTTGTTGCCTCA
      Chironius_bicarinatus_LWS    TTTGCTTATACCAACAGCAACAATACCCGTGACCCTTTTGAAGGTCCAAACTATCAGATTGCACCACGATGGGTCTACAACATAACTTCACTCTGGATGGTCTTTGTTGTTGTTGCCTCA
            Dipsas_petersi_LWS    TTTGTGTACACCAACAGCAACAATACCCGTGGCCCTTTTGAAGGTCCAAACTATCATATTGCACCACGATGGGTCTACAACTTAACTTCACTCTGGATGGTCTTTGTTGTCATTGCCTCA
Echinantera_cephalostriata_LWS    TTTTCTTACACCAACAGCAACAATACCCGTGACCCTTTTGAAGGTCCAAACTATCATATTGCACCACGATGGGTCTACAACATAACTTCACTCTGGATGGTCTTTGTTGTCGTTGCCTCA
      Echinantera_undulata_LWS    TTTTCTTACACCAACAGCAACAATACCCGTGACCCTTTTGAAGGTCCAAACTATCATATTGCACCACGATGGGTCTACAACATAACTTCACTCTGGATGGTCTTTGTTGTCGTTGCCTCA
   Erythrolamprus_aesculapii_LWS    ------TATACCAATAGCAACAATACCCGTGACCCTTTTGAAGGTCCAAACTACCATATTGCACCACGATGGGTCTACAACATAACTTCACTCTGGATGGTCTTTGTTGTCGTTGCCTCA
    Erythrolamprus_miliaris_LWS    TTTGCTTACACCAATAGCAACAATACCCGTGACCCTTTTGAAGGTCCAAACTACCATATTGCACCACGATGGGTCTACAACATAACTTCACTCTGGATGGTCTTTGTTGTCGTTGCCTCA
 Erythrolamprus_poecilogyrus_LWS    TTTGCTTACACCAATAGCAACAATACCCGTGACCCTTTTGAAGGTCCAAACTACCATATTGCACCACGATGGGTCTACAACATAACTTCACTCTGGATGGTCTTTGTTGTCGTTGCCTCA
         Helicops_modestus_LWS    TTTGCTTACACCAACAGCAACAATACCCGTGACCCTTTTGAAGGTCCAAACTATCATATTGCGCCACGATGGGTCTACAACTTAACTTCACTCTGGATGGTCTTTGTTGTCATTGCCTCA
          Oxyrhopus_guibei_LWS    TTTGTTTACACCAACAGCAACAACACCCGTGATCCTTTTGAAGGTCCAAACTATCATATTGCACCACGATGGGTCTACAACCTGACTTCACTCTGGATGGTCTTCGTTGTCATTGCCTCA
   Philodryas_patagoniensis_LWS    TTTGCTTACACCAACAGCAACAATACCCGTGACCCTTTTGAAGGTCCAAACTATCATATTGCCCCACGATGGGTCTACAACATAACTTCACTCTGGATGGTCTTTGTTGTCGTTGCCTCA
              Python_regius_LWS    TTTATTTACACCAACAGCAACAATACCCGTGACCCTTTTGAAGGCCCAAATTATCATATTGCACCACGATGGGTCTACAACTTAACTTCACTCTGGATGGTCTTTGTGGTCATTGCCTCA
     Sibynomorphus_mikanii_LWS    TTTGTGTACACCAACAGCAACAATACCCGTGGCCCTTTTGAAGGTCCAAACTATCATATTGCACCACGATGGGTCTACAACTTAACTTCACTCTGGATGGTCTTTGTTGTCATTGCCTCA
   Sibynomorphus_neuwiedii_LWS    TTTGTGTACACTAACAGCAACAATACCCGTGGCCCTTTTGAAGGTCCAAACTATCATATTGCACCACGATGGGTCTACAACTTAACTTCACTCTGGATGGTCTTTGTTGTCATTGCCTCA
         Spilotes_pullatus_LWS    TTTGCTTATACCAACAGCAACAATACCCGTGACCCTTTTGAAGGTCCAAACTATCATATTGCACCACGATGGGTCTACAACATAACTTCACTCTGGATGGTCTTTGTTGTTATTGCCTCA
   Taeniophallus_persimilis_LWS    TTTTCTTACACCAACAGCAACAATACCCGTGACCCTTTTGAAGGTCCAAACTATCATATTGCACCACGATGGGTCTACAACATAACTTCACTCTGGATGGTCTTTGTTGTCGTTGCCTCA
   Thamnodynastes_strigatus_LWS    TTTACTTACACCAACAGCAACAATACCCGTGACCCTTTTGAAGGTCCAAACTATCATATTGCCCCACGATGGGTCTACAACGTCACTTCACTCTGGATGATCTTTGTTGTCATTGCCTCA
          Tomodon_dorsatus_LWS    TTTACTTACACCAACAGCAACAATACCCGTGACCCTTTTGAAGGTCCAAACTATCATATTGCACCACGATGGGTCTACAAYGTAACTTCACTCTGGATGATCTTTGTTGTCGTTGCCTCA
       Xenopeltis_unicolor_LWS    TTTGTTTACACCAACAGCAACAATACCCGTGACCCTTTTGAAGGCCCAAATTATCACATTGCACCACGATGGGTCTACAACTTAACTTCACTCTGGATGGTCTTTGTTGTCATTGCCTCA
     Atractus_reticulatus_SWS1    ---------------------------------------------------------------------------TTCCACTTCCAAACCATCTTCATGGGCCTGGTCTTTTTTGCCGGG
     Chironius_bicarinatus_SWS1    ------------------------------------------------------------------------------------------------------------TTCTTTGCCGGG
           Dipsas_petersi_SWS1    ---------------------------------------GACGGTCCCCAGTATCACATTGCGCCCATGTGGGCCTTCCACTTCCAAGCCATCTTCATGGGCCTGGTTTTCTTTGCCGGG
Echinantera_cephalostriata_SWS1    -----------------------------------------------------------------------------CCAATTTCAAACCATCTTCATGGGCCTGGTCTTCTTTGCCGGG
     Echinantera_undulata_SWS1    ----------------------------------------------------------------------GGGTCTTCCACTTCCAAACCATCTTCATGGGCCTGGTCTTCTTTGCCGGG
  Erythrolamprus_aesculapii_SWS1    ---------------------------------------GACGGTCCCCAGTATCACATTGCGCCCAAGTGGGCTTTCCACTTCCAAACCATCTTCATGGGCCTGGTTTTCTTCGCCGGG
   Erythrolamprus_miliaris_SWS1    ---------------------------------------GACGGTCCCCAGTATCACATTGCGCCCATGTGGGCTTTCCACTTCCAAACCATCTTCATGGGCCTGGTTTTCTTCGCCGGG
Erythrolamprus_poecilogyrus_SWS1    -------------------------------------------------------------------------CCTTCCACTTCCAAACCATCTTCATGGGCCTGGTTTTCTTCGCCGGG
        Helicops_modestus_SWS1    ------------------------------------------GGTCCCCAATATCACATCGCACCCATGTGGGCTTTCCACTTCCAAACCATCTTCATGGGCCTGGTCTTATTTGCCGGG
         Oxyrhopus_guibei_SWS1    -------------------------------------------------------------------------CTTTCCACTTCCAAACCATCTTCATGGGCCTGGTCTTCTTTGCCGGG
  Philodryas_patagoniensis_SWS1    ------------------------------------------------------------------------------------------------------------------------
             Python_regius_SWS1    ---TACCTCTTTGAAAACATCTCCTCCGTGGGGCCCTGGGACGGTCCCCAGTATCACATTGCCCCCATGTGGGCCTTCCATTTCCAGACCCTCTTCATGGGCTTGGTGTTCTTTGCTGGG
    Sibynomorphus_mikanii_SWS1    -------------------------------------GGGACGGTCCCCAGTATCACATTGCGCCCATGTGGGCCTTCCACTTCCAGGCCATCTTCATGGGCCTGGTTTTCTTTGCCGGG
   Sibynomorphus_neuwiedii_SWS1    ---------------------------------------GACGGTCCCCAGTATCACATTGCGCCCATGTGGGCCTTCCACTTCCAAGCCATCTTCATGGGCCTGGTTTTCTTTGCCGGG
        Spilotes_pullatus_SWS1    -------------------------------------------------------------------------CTTTCCACTTCCAAACCATCTTCATGGGCCTGGTTTTCTTTGCCGGG
  Taeniophallus_persimilis_SWS1    ---------------------------------------------------------------------------------TTCCAAACCATCTTCATGGGCCTGGTCTTCTTTGCCGGG
  Thamnodynastes_strigatus_SWS1    --------------------------------------------------------------------------CTTCCACTTCCAAACCATCTTCATGGGCCTGGTCTTCTTTGCCGGG
         Tomodon_dorsatus_SWS1    --------------------------CGTGAGGCCCTGGGATGGCCCTCAATATCACATCGCGCCCATGTGGGCTTTCCACTTCCAAACCATCTTCATGGGCCTGGTCTTCTTTGCCGGG
      Xenopeltis_unicolor_SWS1    TTCTACCTCTTTGAAAACATCTCCTCCGTGGGGCCCTGGGACGGTCCCCAGTATCACATTGCCCCCATGTGGGCCTTCCGCTTCCAAACCGCCTTCATGGGCTTGGTGTTCTTTGCCGGG
```

```
                                     251       260        270        280        290        300        310        320        330        340        350       360        370
                                     |         |          |          |          |          |          |          |          |          |          |         |          |
                 Bos_taurus_Rh1      TTCCCCATCAACTTCCTCACGCTGTACGTCACAGTCCAGCACAAGAAGCTGCGCACACCCCTCAACTACATCCTGCTCAACCTGGCCGTGGCCGACCTC---TTCATGGTCTTCGGGGGC
       Atractus_reticulatus_Rh1      TTTCCCATCAATTTTCTAACCCTCTATGTCACCATCCAACACAAGAAACTCAGAACACCCCTTAACTACATCTTGTTAAATTTGGCAATAGCCAATCTA---TTCATGGTCTTGGCTGGC
        Chironius_bicarinatus_Rh1    TTTCCCATCAATTTTCTAACCCTCTATGTCACCATCCAACACAAGAAACTCAGAACACCCTTGAACTACATCCTGTTAAATTTGGCAGTGGCCAATCTA---TTCATGGTCTTGGTTGGC
             Dipsas_petersi_Rh1      TTTCCCATCAATTTTCTAACCCTCTTTGTCACCATTCAACACAAGAAACTCAGAACGGCCCTGAACTACATCTTGTTAAATTTGGCAGTAGCCGATCTA---TTCATGGTCTTGGCTGGC
Echinantera_cephalostriata_Rh1      TTTCCCATCAATTTTCTAACCCTCTATGTCACCATCCAACACAAGAAACTCAGAACACCCCTGAACTACATCCTGTTAAATTTGGCAGTAGCCAATCTA---TTCATGGTCTTGGTTGGC
      Echinantera_undulata_Rh1      TTTCCCATCAATTTTCTAACCCTCTATGTCACCATCCAACACAAGAAACTCAGAACACCTCTGAACTACATCCTGTTAAATTTGGCAGTAGCCAATCTA---TTCATGGTCTTGGTTGGC
  Erythrolamprus_aesculapii_Rh1      TTTCCCATCAATTTTCTAACCCTATATGTCACCATCCAACACAAGAAACTCAGAACACCCTTGAACTACATCCTGTTAAATTTGGCAGTAGCCAATCTA---TTCATGGTCTTGGTTGGC
    Erythrolamprus_miliaris_Rh1      TTTCCCATCAATTTTCTAACCCTATATGTCACCATCCAACACAAGAAACTCAGAACACCTTTGAACTACATCCTGTTAAATTTGGCAGTAGCCAATCTA---TTCATGGTCTTGGTTGGC
Erythrolamprus_poecilogyrus_Rh1      --------TCATTTTCTAACCCTATATGTCACCATCCAACACAAGAAACTCAGAACACCTTTGAACTACATCCTGTTAAATTTGGCAGTAGCCAATCTA---TTCATGGTCTTGGTTGGC
          Helicops_modestus_Rh1      TTTCCCATCAATTTTCTAACCCTCTATGTCACCATCCAACACAAGAAACTCAGAACACCCCTGAACTACATCCTGTTAAATTTGGCAGTAGCCAATCTA---TTCATGGTCTTGGTTGGC
           Oxyrhopus_guibei_Rh1      TTTCCCATCAATTTTCTAACCCTCTATGTCACCATCCAACACAAGAAACTCAGAACAGCCCTGAACTACATCCTGTTAAATTTGGCAGTAGCCGATCTT---TTCATGGTCTTGGCTGGC
   Philodryas_patagoniensis_Rh1      TTTCCCATCAATTTTCTAACCCTCTTTGTCACCATCCAACACAAGAAACTCAGAACACCCCTGAACTACATCCTGTTAAATTTGGCAGTAGCCAATCTA---TTTATGGTCTTGGTTGGC
              Python_regius_Rh1      TTTCCTATCAACTTTCTAACTCTCTATGTCACCATCCAACACAAGAAACTCAGAACACCTCTGAACTACATCCTGTTAAATTTGGCAATAGCCAACCTA---TTCATGGTCTTAGTTGGC
       Sibynomorphus_mikanii_Rh1     TTTCCCATCAATTTTCTAACCCTCTTTGTCACCATTCAACACAAGAAACTCAGAACGGCCCTGAACTACATCCTGTTAAATTTGGCAGTAGCCGATCTA---TTCATGGTCTTGGCTGGC
     Sibynomorphus_neuwiedi_Rh1      TTTCCCATCAATTTTCTAACCCTCTTTGTCACCATTCAACACAAGAAACTCAGAACGGCCCTGAACTACATCCTGTTAAATTTGGCAGTAGCCGATCTA---TTCATGGTCTTGGCTGGM
          Spilotes_pullatus_Rh1      TTTCCCATCAATTTTCTAACCCTCTATGTCACCATCCAACACAAGAAACTCAGAACACCCTTGAACTACATCCTGTTAAATTTGGCAGTGGCCAATCTA---TTCATGGTCTTGGTTGGC
    Taeniophallus_persimilis_Rh1     TTTCCCATCAATTTTCTAACCCTCTATGTCACCATCCAACACAAGAAACTCAGAACACCCCTGAACTACATCCTGTTAAATTTGGCAGTAGCCAATCTA---TTCATGGTCTTGGTTGGC
    Thamnodynastes_strigatus_Rh1     TTTCCCATCAATTTTCTAACCCTCTATGTCACCATCCAACACAAGAAACTCAGAACACCCCTGAACTACATCCTGTTAAATTTGGCAGTAGCCAATCTA---TTCATGGTCTTGGTTGGC
           Tomodon_dorsatus_Rh1      TTTCCCATCAATTTTCTAACCCTCTATGTCACCATCCAACACAAGAAACTCAGAACACCTCTGAACTACATCCTGTTAAATTTGGCAGTAGCCAATCTA---TTCATGGTCTTGGTTGGC
        Xenopeltis_unicolor_Rh1      TTTCCTATCAACTTTCTAACTCTCTATGTCACCATCCAACACAAGAAGCTCAGAACACCCCTGAACTACATCCTGTTAAATTTGGCAACCGCTAATCTA---TTCATGGTCTTAGTTGGC
       Atractus_reticulatus_LWS      GTCTTTACCAATGGTTTGGTATTGGTGGCCACAGCCAAATTCAAGAAATTACGGCATCCGCTCAACTGGATTTTGGTGAACTTGGCTATAGCTGATTTGGGTGAAACTGTTATTGCTAGC
        Chironius_bicarinatus_LWS    GTCTTTACCAATGGTTTGGTATTGGTGGCCACAGCCAAATTCAAGAAATTACGGCATCCACTCAACTGGATTTTGGTGAACTTGGCTATAGCTGATTTGGGTGAAACGGTTATTGCTAGC
             Dipsas_petersi_LWS      GTCTTTACCAATGGTTTGGTATTGGTGGCCACAGCCAAATTCAAGAAATTACGACATCCGCTCAACTGGATTTTGGTGAACTTGGCTATAGCTGATTTGGGTGAAACGGTTATTGCTAGC
Echinantera_cephalostriata_LWS      GTCTTTACCAATGGTTTGGTATTGGTGGCCACAGCCAAATTCAAGAAATTACGGCATCCGCTCAACTGGATTTTGGTGAACTTGGCTTTAGCTGATTTGGGTGAAACGGTTATTGCTAGC
      Echinantera_undulata_LWS      GTCTTTACCAATGGTTTGGTATTGGTGGCCACAGCCAAATTCAAGAAATTACGGCATCCGCTCAACTGGATTTTGGTGAACTTGGCTCTAGCTGATTTGGGTGAAACGGTTATTGCTAGC
  Erythrolamprus_aesculapii_LWS      GTCTTTACCAATGGTTTGGTATTGGTGGCCACAGCCAAATTCAAGAAATTACGGCATCCCCTCAACTGGATTTTGGTGAACTTGGCTATAGCTGATTTGGGTGAAACGGTTATTGCTAGC
    Erythrolamprus_miliaris_LWS      GTCTTTACCAATGGTTTGGTATTGGTGGCCACAGCCAAATTCAAGAAATTACGGCATCCCCTCAACTGGATTTTGGTGAACTTGGCTATAGCTGATTTGGGTGAAACGGTTATTGCTAGC
Erythrolamprus_poecilogyrus_LWS      GTCTTTACCAATGGTTTGGTATTGGTGGCCACAGCCAAATTCAAGAAATTACGGCATCCGCTCAACTGGATTTTGGTGAACTTGGCTATAGCTGATTTGGGTGAAACGGTTATTGCTAGC
          Helicops_modestus_LWS      GTCTTTACCAATGGTTTGGTATTGGTGGCAACAGCCAAATTCAAGAAATTACGGCATCCGCTTAACTGGATTTTGGTGAACTTGGCTATAGCTGATTTGGGGGAAACGGTTATTGCTAGC
           Oxyrhopus_guibei_LWS      GTCTTTACCAATGGTTTGGTATTGGTGGCCACAGCCAAATTCAAGAAATTACGACATCCGCTCAACTGGATTTTGGTGAACTTAGCTATAGCTGATTTGGGTGAAACGGTTATTGCTAGC
   Philodryas_patagoniensis_LWS      GTCTTTACCAATGGTTTGGTATTGGTGGCCACAGCCAAATTCAAGAAATTACGGCATCCGCTCAACTGGATTTTGGTGAACTTGGCTATCGCTGATTTGGGTGAAACGGTTATTGCTAGT
              Python_regius_LWS      GTCTTTACCAATGGTTTGGTCTTGGTGGCCACAGCCAAATTCAAGAAATTACGGCACCCGCTCAACTGGATTCTGGTGAACTTGGCTATAGCTGATTTGGGTGAAACAGTTATTGCTAGC
       Sibynomorphus_mikanii_LWS     GTCTTTACCAATGGTTTGGTATTGGTGGCCACAGCCAAATTCAAGAAATTACGACATCCGCTCAACTGGATTTTGGTGAACTTGGCTATAGCTGATTTGGGTGAAACAGTTATTGCTAGC
    Sibynomorphus_neuwiedii_LWS      GTCTTCACCAATGGTTTGGTATTGGTGGCCACAGCCAAATTCAAGAAATTACGACATCCGCTCAACTGGATTTTGGTGAACTTGGCTATAGCTGATTTGGGTGAAACGGTTATTGCTAGC
          Spilotes_pullatus_LWS      GTCTTTACCAATGGTTTGGTATTGGTGGCCACAGCCAAATTCAAGAAATTACGGCATCCGCTCAACTGGATTTTGGTGAACTTGGCTATAGCTGATTTGGGTGAAACGGTTATTGCTAGC
    Taeniophallus_persimilis_LWS     GTCTTTACCAATGGTTTGGTATTGGTGGCCACAGCCAAATTCAAGAAATTACGGCATCCGCTCAACTGGATTTTGGTGAACTTGGCTCTAGCTGATTTGGGTGAAACGGTTATTGCTAGC
    Thamnodynastes_strigatus_LWS     GTCTTTACCAATGGTTTGGTATTGGTGGCCACAGCCAAATTCAAGAAATTACGGCATCCGCTTAACTGGATTTTGGTGAACTTGGCTATAGCTGATTTGGGTGAAACCGTTATTGCTAGT
           Tomodon_dorsatus_LWS      GTCTTCACCAATGGTTTGGTATTGGTGGCCACAGCCAAATTCAAGAAATTACGGCATCCGCTTAACTGGATTTTGGTGAACTTGGCTATAGCTGATTTGGGTGAAACGGTTATTGCTAGT
        Xenopeltis_unicolor_LWS      GTCTTTACCAATGGCTTGGTATTGGTGGCCACAGCCAAATTCAAGAAGTTACGGCATCCGCTCAACTGGATTCTAGTGAACTTGGCTATTGCTGATTTGGGTGAAACGGTTATTGCTAGC
      Atractus_reticulatus_SWS1      ACCCCCCTCAACGCCATCATCCTCTTCGTCACCATCAAATACAAGAAGTTGAGGCAGCCGCTCAACTACATCTTGGTCAACATCTCCTTGGCCGGATTGATCTTCTGCATCTTTGCGGTT
       Chironius_bicarinatus_SWS1    ACCCCCCTCAACGGCATCATCCTCTTTGTCACCATCAAGTACAAGAAGTTGAGGCAGCCGCTCAACTACATCTTGGTCAACATCTCTTTTGCTGGATTTATGGTCTGCATATTTGCAATT
            Dipsas_petersi_SWS1      ACGCCCCTCAACGCCATCATCCTCTTCGTCACCATCAAGTACAAGAAGTTGAGGCAGCCGCTCAACTACATCTTGGTCAACATCTCCTTGGCCGGATTGATCTTCTGCATCTTTGCGGTT
Echinantera_cephalostriata_SWS1     ACGCCCCTCAAYGCCATCATCCTCTTCGTCACCATCAAGTACAAGAAGTTGAGGCAGCCGCTCAACTACATCTTGGTCAACATCTCCTTTGCCGGATTTATCTTCTGCACCTTTGCGGTT
     Echinantera_undulata_SWS1      ACGCCCCTCAACGCCATCATCCTCTTCGTCACCATCAAGTACAAGAAGTTGAGGCAGCCGCTCAACTACATCTTGGTCAACATCTCCTTTGCCGGATTTATCTTCTGCACCTTTGCGGTT
  Erythrolamprus_aesculapii_SWS1     ACGCCCCTCAACGCCATCATCCTCTTCGTCACCATCAAGTACAAGAAGTTGAGGCAGCCGCTCAACTACATCTTGGTCAACATCTCCTTTGCCGGATTTATCTTCTGCATCTTTGCCGTT
    Erythrolamprus_miliaris_SWS1     ACGCCCCTCAACGCCATCATCCTCTTCGTCACCGTCAAGTACAAGAAGTTGAGGCAGCCGCTCAACTACATCTTGGTCAACATCTCCTTTGCCGGATTTATCTTCTGCATCTTCGCCGTT
Erythrolamprus_poecilogyrus_SWS1     ACGCCCCTCAACGCCATCATCCTCTTCGTCACTGTCAAGTACAAGAAGTTGAGGCAGCCGCTCAACTACATCTTGGTCAACATCTCCTTTGCCGGATTTATCTTCTGCATCTTCGCCGTT
          Helicops_modestus_SWS1     ACGCCCCTCAACGCCRTCATCCTCTTCGTCACCATCAAGTACAAGAAGTTGAGGCAGCCGCTCAACTACATCTTGGTCAACATYTCCTTTGCCGGATTTATCKTCTGCGTCTTTGCGRTT
           Oxyrhopus_guibei_SWS1     ACGCCCCTCAACTGCATCATCCTCTTCGTCACCGTCAAATACAAGAAGTTGAGGCAGCCGCTCAACTACATCTTGGTCAACATCTCCTTTGCCGGACTTATCTTCTGCACTTTTGCGGTT
   Philodryas_patagoniensis_SWS1     ----------------------TCTTTGTCACCATCAAGTACAAGAAATTGAGGCAGCCGCTCAACTACATCTTGGTCAACATCTCCTTTGCCGGATTTATCTTCTGCATCTTTGCGGTT
              Python_regius_SWS1     ACCCCCCTCAACGCCACCATCCTCATTGTCACCATTAAGTACAAGAAGCTGCGGCAGCCGCTGAACTACATCTTGGTCAACATCTCTTTTGCCGGACTCCTCTTCTGTGTCTTCGCCGTC
       Sibynomorphus_mikanii_SWS1    ACGCCCCTCAACGCCATCATCCTCTTCGTCACCATCAAGTACAAGAAGTTGAGGCAGCCGCTCAACTACATCTTGGTCAACATCTCCTTGGCCGGATTGATCTTTTGCATCTTTGCGGTT
     Sibynomorphus_neuwiedii_SWS1    ACGCCGCTCAACGCCATCATCCTCTTCGTCACCATCAAGTACAAGAAGTTGAGGCAGCCGCTCAACTACATCTTGGTCAACATCTCCTTGGCCGGATTGATCTTCTGCATCTTTGCGGTT
          Spilotes_pullatus_SWS1     ACGCCCCTCAACGCCATCATCCTCTTCGTCACCATCAAGTACAAGAAGTTGAGGCAGCCGCTCAACTACATCTTGGTCAACATCTCCTTTGCTGGACTTATCTTCTGCGTCTTTGCGGTT
    Taeniophallus_persimilis_SWS1    ACGCCCCTCAACGCCATCATCCTCTTCGTCACCATCAAGTACAAGAAGTTGAGGCAGCCGCTCAACTACATCTTGGTCAACATCTCCTTTGCCGGATTTATCTTCTGCACCTTTGCGGTT
    Thamnodynastes_strigatus_SWS1    ACGCCCCTCAACGCCATCATCCTCTTCGTCACCATCAAGTACAAGAAGTTGAGGCAGCCGCTCAACTACATCTTGGTCAACATCTCCTTTGCCGGATTGATCTTCTGCGTCTTTGCGGTT
           Tomodon_dorsatus_SWS1     ACGCCCCTCAACGCCATCATCCTCTTCGTCACCATCAAGTACAAGAAGTTGAGGCAGCCGCTCAACTACATCTTGGTCAACATCTCCTTTGCCGGATTGATMTTCTGCGTCTTTGCGGTW
        Xenopeltis_unicolor_SWS1     ACCCCCCTCAACGCCACCATCCTCATTGTCACCATTAAGTATAAGAAGCTGCGGCAGCCGCTGAACTACATCTTAGTCAACATCTCTTTCGCTGGGCTCCTCTTCTGCGTCTTCGCCATC
```

```
                                 371       380        390        400        410        420        430        440        450        460        470        480        490
                                 |         |          |          |          |          |          |          |          |          |          |          |          |
Bos_taurus_Rh1                   TTCACCACCACCCT-CTACACCTCTCTGCACGGGTACTTCGTCTTTGGGCCCACGGGCTGCAACCTGGAGGGCTTCTTTGCCACCTTGGGCGGTGAAATTGCACTGTGGTCCTTGGTGGT
Atractus_reticulatus_Rh1         TTCACCACTACTAT-GTACACTTCCATGAATGGATATTTCGTTTTTGGGATAGTAGGATGCAACATTGAAGGCTTTTTTGCTACATTAGGTGGTGAGATTGCTCTTTGGTCTCTCGTAGT
Chironius_bicarinatus_Rh1        TTCACCACTACCAT-GTACACTTCCATGAATGGATATTTCATTTTTGGGACAGTAGGATGCAACGTTGAAGGCTTCTTTGCTACATTGGGTGGTGAGATAGCTCTCTGGTCTCTCGTAGT
Dipsas_petersi_Rh1               TTCACCACTACTAT-GTACACTTCCATGAATGGATATTTCGTTTTTGGGGTAGTTGGATGCAACATTGAAGGCTTCTTTGCTACATTAGGTGGTGAGATAGCTCTTTGGTCTCTCGTAGT
Echinantera_cephalostriata_Rh1   TTCACCACTACCAT-GTACACTTCCATGAATGGATATTTCATTTTTGGGACAATAGGATGCAACGTTGAAGGCTTCTTTGCTACATTAGGTGGTGAGATAGCTCTTTGGTCTCTCGTAGT
Echinantera_undulata_Rh1         TTCACCACTACCAT-GTACACTTCCATGAATGGATATTTCATTTTTGGGACAATAGGATGCAACGTTGAAGGCTTCTTTGCTACATTAGGTGGTGAGATAGCTCTTTGGTCTCTCGTAGT
Erythrolamprus_aesculapii_Rh1    TTCACCACTACCAT-GTACACTTCAATGAATGGATATTTCATTTTTGGGACAATAGGATGCAACGTTGAAGGCTTTTTTGCTACATTAGGTGGTGAGATAGCTCTTTGGTCTCTCGTAGT
Erythrolamprus_miliaris_Rh1      TTCACCACTACCAT-GTACACTTCCATGAATGGATATTTCATTTTTGGGACAATAGGATGCAACGTTGAAGGCTTTTTTGCTACATTAGGTGGTGAGATAGCTCTTTGGTCTCTTGTAGT
Erythrolamprus_poecilogyrus_Rh1  TTCACCACTACCAT-GTACACTTCCATGAATGGATATTTCATTTTTGGGACAATAGGATGCAACGTTGAAGGCTTTTTTGCTACATTAGGTGGTGAGATAGCTCTTTGGTCTCTTGTAGT
Helicops_modestus_Rh1            TTCACCACTACCAT-GTACACTTCCATGAATGGATATTTCATTTTTGGGACAATAGGATGCAACGTTGAAGGCTTCTTTGCTACATTAGGTGGTGAAATAGCTCTTTGGTCTCTCGTAGT
Oxyrhopus_guibei_Rh1             TTCACCACTACCAT-GTACACTTCCATGAATGGATATTTCGTTTTTGGGACAGTAGGATGCAACATTGAAGGCTTCTTTGCTACATTAGGTGGTGAGATAGCTCTTTGGTCTCTCGTAGT
Philodryas_patagoniensis_Rh1     TTCACCACTACCAT-GTACACTTCCATGAATGGGTATTTCGTTTTTGGGACAATAGGATGCAACGTTGAAGGCTTCTTTGCTACATTAGGTGGTGAGATAGCTCTTTGGTCTCTCGTAGT
Python_regius_Rh1                TTCACCACCACCAT-GTACACTTCCATGAATGGATATTTCGTTTTTGGGACGGTAGGATGCAACGTTGAAGGCTTCTTTGCAACATTGGGCGGTGAGATAGCCCTTTGGTCTCTTGTGGT
Sibynomorphus_mikanii_Rh1        TTCACCACTACTAT-GTACACTTCCATGAATGGATATTTCGTTTTTGGGGTAGTTGGATGCAACATTGAAGGCTTCTTTGCTACATTAGGTGGTGAGATAGCTCTCTGGTCTCTCGTAGT
Sibynomorphus_neuwiedi_Rh1       TTCACCACTACTAT-GTACACTTCCATGAATGGATATTTCGTTTTTGGGGTAGTTGGATGCAACATTGAAGGCTTCTTTGCTACATTAGGTGGTGAGATAGCTCTTTGGTCTCTCGTAGT
Spilotes_pullatus_Rh1            TTCACCACTACCAT-GTATACTTCCATGAATGGATATTTCATTTTTGGGACAGTAGGATGCAACGTTGAAGGCTTCTTTGCTACATTGGGTGGTGAGATAGCTCTTTGGTCTCTCGTAGT
Taeniophallus_persimilis_Rh1     TTCACCACTACCAT-GTACACTTCCATGAATGGATATTTCATTTTTGGGACAATAGGATGCAACGTTGAAGGCTTCTTTGCTACATTAGGTGGTGAGATAGCTCTTTGGTCTCTCGTAGT
Thamnodynastes_strigatus_Rh1     TTCACCACTACCAT-GTACACTTCCATGAATGGATATTTTATTTTTGGGACAATAGGATGCAACGTTGAAGGCTTCTTTGCTACATTAGGTGGTGAGATAGGTCTTTGGTCTCTCGTAGT
Tomodon_dorsatus_Rh1             TTCACCACTACCAT-GTACACTTCCATGAATGGATATTTTATTTTTGGGACAATAGGATGCAACGTTGAAGGCTTCTTTGCTACATTAGGTGGTGAGATAGGTCTTTGGTCTCTCGTAGT
Xenopeltis_unicolor_Rh1          TTCACCACCACCAT-GTACACTTCCATGAATGGATATTTCGTTTTTGGGACAGTAGGATGCAACGTTGAAGGCTTCTTTGCTACATTGGGCGGTGAGATGGCTCTTTGGTCTCTCGTAGT
Atractus_reticulatus_LWS         ACCATCAGTGTCATCAACCAGTTCT----TTGGCTATTTCGTCCTTGGCCATCCTTTGTGTGTTGTGGAGGGTTATACTGTCTCTGTTTGTGGCATTACAGCTCTCTGGTCTTTGGCCAT
Chironius_bicarinatus_LWS        ACTATCAGTGTCATCAACCAGTTCT----TTGGCTATTTCATCCTTGGCCATCCTATGTGTGTTTTGGAGGGTTATACTGTCTCTGCTTGTGGCATTACAGCTCTCTGGTCTTTGGCCAT
Dipsas_petersi_LWS               ACCATCAGTGTCATCAACCAGTTCT----TTGGCTATTTCGTCCTTGGCCATCCTTTGTGTGTTGTGGAGGGTTATACTGTCTCTGTTTGTGGCATTACAGGTCTCTGGTCTTTGGCCAT
Echinantera_cephalostriata_LWS   ACCATCAGTGTCATCAACCAGTTCT----TTGGCTATTTCATCCTTGGCCATCCTATGTGTGTTTTGGAGGGTTATACTGTCTCTGCTTGTGGCATTACAGCTCTCTGGTCTTTGGCCAT
Echinantera_undulata_LWS         ACCATCAGTGTYATCAACCAGTTCT----TTGGCTATTTCATCCTTGGCCATCCTATGTGTGTTTTGGAGGGTTATACTGTCTCTGCTTGTGGCATTACAGCTCTCTGGTCTTTGGCCAT
Erythrolamprus_aesculapii_LWS    ACCATCAGTGTCATCAACCAGTTCT----TTGGTTATTTCGTCCTTGGACATCCTTTGTGTGTTTTGGAGGGTTATACTGTCTCTGCTTGCGGCATTACAGCTCTCTGGTCTTTGGCCAT
Erythrolamprus_miliaris_LWS      ACCATCAGTGTCATCAACCAGTTCT----TTGGCTATTTCGTCCTTGGCCATCCTTTGTGTGTTTTGGAGGGTTATACTGTCTCTGCTTGCGGCATTACAGCTCTCTGGTCTTTGGCCAT
Erythrolamprus_poecilogyrus_LWS  ACCATCAGTGTCATCAACCAGTTCT----TTGGCTATTTCGTCCTTGGCCATCCTTATGTGTTTTGGAGGGTTATACTGTCTCTGCTTGTGGCATTACAGCTCTCTGGTCTTTGGCCAT
Helicops_modestus_LWS            ACTATCAGTGTCATCAACCAGTTCT----TTGGCTATTTCATCCTTGGCCACCCGTTGTGTGTTTTGGAGGGTTATACTGTCTCTGCTTGTGGCATTACAGCTCTCTGGTCTTTGGCCAT
Oxyrhopus_guibei_LWS             ACCATCAGCGTCATCAACCAGTTCT----TTGGCTATTTCATCCTTGGCCATCCTTTGTGTGTTGTGGAGGGTTATACTGTCTCTGTTTGTGGCATTACAGGTCTCTGGTCTTTGGCCAT
Philodryas_patagoniensis_LWS     ACCATCAGTGTCATCAACCAGTTCT----TTGGCTATTTCGTCCTTGGCCATCCTTTGTGTGTTTTGGAGGGTTATACTGTCTCTGTTTGTGGAATTACAGCTCTCTGGTCTTTGGCCAT
Python_regius_LWS                ACCATCAGTGTCATCAACCAGTTCT----TTGGCTATTTCATCCTTGGCCATCCCTTGTGTGTTGTGGAGGGTTATACTGTCTCTGTCTGTGGCATCACAGGCCTCTGGTCTTTGGCCAT
Sibynomorphus_mikanii_LWS        ACCATCAGTGTCATCAACCAGTTCT----TTGGCTATTTCGTCCTTGGCCATCCTTTGTGTGTTGTGGAGGGTTATACTGTCTCTGTTTGTGGCATTACAGCTCTCTGGTCTTTGGCCAT
Sibynomorphus_neuwiedii_LWS      ACCATCAGTGTCATCAACCAGTTCT----TTGGCTATTTCGTCCTTGGCCATCCTTTGTGTGTTGTGGAGGGTTATACYGTCTCTGTTTGTGGCATTACAGGTCTCTGGTCTTTGGCCAT
Spilotes_pullatus_LWS            ACTATCAGTGTCGTCAACCAGTTCT----TTGGCTATTTCATCCTTGGCCATCCTATGTGTGTTTTGGAGGGTTATACTGTCTCTGCTTGTGGCATTACAGCTCTCTGGTCTTTGGCCAT
Taeniophallus_persimilis_LWS     ACCATCAGTGTCATCAACCAGTTCT----TTGGCTATTTCATCCTTGGCCATCCTATGTGTGTTTTGGAGGGTTATACTGTCTCTGCTTGTGGCATTACAGCTCTCTGGTCTTTGGCCAT
Thamnodynastes_strigatus_LWS     ACCATCAGTGTCATCAATCAATTCT----TTGGCTATTTCTACCTTGGCCATCCTTTGTGTGTTCTGGAGGGTTATACTGTCTCTGTTTGTGGCATTACAGCTCTCTGGTCTTTGGCCAT
Tomodon_dorsatus_LWS             ACCATCAGTGTCATCAACCAGTTCT----TTGGCTATTTCTACCTTGGCCATCCTTTGTGTGTTCTGGAGGGTTATACTGTCTCTGCTTGTGGCATTACAGCTCTCTGGTCTTTGGCCAT
Xenopeltis_unicolor_LWS          ACCATCAGTGTCATCAACCAGTTCT----TTGGCTATTTCATCCTTGGCCATCCATGTGTGTTGTGGAGGGTTATACAGTCTCTGTTTGTGGCATCACAGCCCTCTGGTCTTTGGCCAT
Atractus_reticulatus_SWS1        TTCACGGTCTTCTT-GTCCAGCACC---CAAGGTTACTTCTTCTTCGGCCGACAGGTCTGCAGGTTGGAGGCCTTCTAGGCACGGTGGCAGGTATGGTCACTGGATGGTCCTTGGCCTT
Chironius_bicarinatus_SWS1       CTTGTGGTCTTCCT-GTCCAGCATG---CATGGTTACTTCTTCTTGGGTCGTCAGTTCTGCAAGATGGAGGCCTTCCTAGGCACGGTAGCAGGTATGGTCACCGGCTGGTCCTTGGCCTT
Dipsas_petersi_SWS1              TTCACGGTCTTCTT-GTCCAGCACT---CAAGGTTACTTCTTCTTTGGCCGACAGGTCTGCAGGTTGGAGGCCTTCCTGGGCACGGTGGCAGGTATGGTCACCGGCTGGTCCTTGGCCTT
Echinantera_cephalostriata_SWS1  TTCGTGGTCTTCTT-GTCCAGCTCC---CAAGGTTACTTCTTCTTTGGTCGACAGGTCTGCAGGTTGGAGGCCTTCCTAGGCACGGTGGCAGGTATGGTCACCGGCTGGTCCTTGGCCTT
Echinantera_undulata_SWS1        TTCGTGGTCTTCTT-GTCCAGCTCC---CAAGGTTACTTCTTCTTCGGTCGACAGGTCTGCAGGTTGGAGGCCTTCCTAGGCACGGTGGCAGGTATGGTCACCGGCTGGTCCTTGGCCTT
Erythrolamprus_aesculapii_SWS1   TTCACCGTCTTCTT-GTCCAGCTCC---CAAGGTTATTTCTTCTTCGGACGACAAGTCTGTAGGTTGGAGGCCTTCCTAGGCACGGTGGCAGGTATGGTCACCGGCTGGTCTTTGGCCTT
Erythrolamprus_miliaris_SWS1     TTCACCGTCTTCCT-GTCCAGCTCC---CAAGGTTACTTCTTCTTCGGACGACAAGTCTGTAGGTTGGAGGCCTTCCTAGGCACTGTGGCAGGTATGGTCACCGGSTGGTCCTTGGCCTT
Erythrolamprus_poecilogyrus_SWS1 TTCACCGTCTTCTT-GTCCAGCTCC---CAAGGTTACTTCTTCTTCGGACGACAAGTCTGTAGGTTGGAGGCCTTCCTAGGCACGGTGGCAGGTATGGTCACCGGCTGGTCCTTGGCCTT
Helicops_modestus_SWS1           TTYRTGGTCTTCTT-ATCCAGCACC---CAAGGTTACTTCTTCCTTGGTCGACAGTTCTGCARGATRGAAGCCTTCCTAGGCACGGTGGCAGGAATGGTCACCGGCTGGTCCTTGGCCTT
Oxyrhopus_guibei_SWS1            TCCGTGGTCTTCTT-GTCCAGCTCC---CAAGGTTACTTCTTCTTTGGTCGACAGGTCTGCAAGTTGGAGGCCTTCCTAGGCACGGTGGCAGGTATGGTCACCGGCTGGTCCTTGGCCTT
Philodryas_patagoniensis_SWS1    TTCACGGTCTTCTT-GTCCAGCTCT---CAAGGTTACTTCTTCTTTGGTCGACAGGTCTGCAGGTTGGAGGCCTTCCTAGGCACGGTGGCAGGTATGGTCACCGGCTGGTCCTTGGCCTT
Python_regius_SWS1               TTCACCGTCTTCTT-GGCCAGCTCC---CAGGGCTACTTCTTCTTCGGGCGCCACGTCTGTGCCTTGGAGGCCTTCCTGGGCTCGGTGGCAGGTCTGGTCACCGGTTGGTCCTTGGCCTT
Sibynomorphus_mikanii_SWS1       TTCACGGTCTTCTT-GTCCAGCACT---CAAGGTTACTTCTTCTTTGGCCGGCAGGTCTGCAGGTTGGAGGCCTTCCTAGGCACAGTGGCAGGTATGGTCACCGGCTGGTCCTTGGCCTT
Sibynomorphus_neuwiedii_SWS1     TTCACGGTCTTCTT-GTCCAGCACT---CAAGGGTACTTCTTCTTTGGCCGACAGGTCTGCAGGTTGGAGGCCTTCCTAGGCACGGTGGCAGGCATGGTCACYGGCTGGTCCTTGGCCTT
Spilotes_pullatus_SWS1           TTTGTGGTCTTCCT-GTCCAGCATG---CAAGGTTACTTCTTCTTGGGTCGTCAGTTCTGCAAGATGGACGCCTTCCTAGGCACAGTAGCAGGTCTGGTCACCGGCTGGTCCTTGGCCTT
Taeniophallus_persimilis_SWS1    TTCGTGGTCTTCTT-GTCCAGCTCC---CAAGGTTACTTCTTCTTTGGTCGACAGGTCTGCAGGTTGGAGGCCTTCCTAGGCACGGTGGCAGGTATGGTCACCGGCTGGTCCTTGGCCTT
Thamnodynastes_strigatus_SWS1    TTCACGGTCTTTTT-GTCCAGCACC---CAAGGTTATTTCTTCTTTGGTCGACAGGTCTGCAGGTTGGAGGCCTTCCTAGGCACGGTGGCAGGTATGGTCACCGGCTGGTCCTTGGCCTT
Tomodon_dorsatus_SWS1            TTCACGGTCTTCTT-ATCCAGCACC---CAAGGTTATTTCTTCTTTGGTCGACAGGTCTGCAGGTTGGAGGCCTTCCTAGGCACGGTGGCAGGTATGGTCACCGGCTGGTCCTTGGCCTT
Xenopeltis_unicolor_SWS1         TTCACCGTCTTCTT-GGCCAGCTCC---CAAGGCTACTTCTTCTTCGGGCGTCACGTCTGTGCCTTGGAGGCCTTCCTGGGCTCGGTGGCAGGTCTGGTCACCGGCTGGTCCTTGGCCTT
```

```
                                     491       500       510       520       530       540       550       560       570       580       590       600       610
                                       |         |         |         |         |         |         |         |         |         |         |         |         |
                      Bos_taurus_Rh1   CCTGGCCATCGAGCGGTACGTGGTGGTGTGCAAGCCCATGAGCAACTTCCGCTTCGGGGAGAACCACGCCATCATGGGCGTCGCCTTCACCTGGGTCATGGCTCTGGCCTGTGCCGCGCC
           Atractus_reticulatus_Rh1   CCTGGCTGTAGAAAGATATGTAGTAGTTTGTAAGCCCATGAGCAACTTCCGTTTCACCGAAACTCACGCCATCATGGGAGTGGCCTTCACTTGGATCATGGCATTGGCTTGTGCTGCTCC
          Chironius_bicarinatus_Rh1   CCTGGCTGTAGAAAGATATGTAGTAGTTTGTAAGCCCATGAGTAACTTCCGTTTCACCGAAACTCATGCCATCATGGGACTGTCTCTCACTTGGATCATGGCCTTGGCTTGTGCTGCTCC
                 Dipsas_petersi_Rh1   CCTGGCTATAGAAAGATATGTAGTAGTTTGTAAGCCCATGAGCAACTTCCGTTTCACCGAAACTCACGCCATCATGGGAGTGGCCTTCACTTGGATCATGGCATTGGCTTGTGCTGCTCC
     Echinantera_cephalostriata_Rh1   CCTGGCTGTAGAAAGATATGTAGTAGTTTGTAAGCCCATGAGCAACTTCCGTTTCACCGAAACTCACGCCATCATGGGAGTGTCCCTCACTTGGATCATGGCGTTGGCTTGTGCTGCTCC
           Echinantera_undulata_Rh1   CCTGGCTGTAGAAAGATATGTAGTAGTTTGTAAGCCCATGAGCAACTTCCGTTTCACCGAAACTCACGCCATCATGGGAGTGTCCCTCACTTGGATCATGGCGTTGGCTTGTGCTGCGCC
      Erythrolamprus_aesculapii_Rh1   CCTGGCTGTAGAAAGATATGTAGTAGTTTGTAAGCCCATGAGCAACTTCCGTTTCACCGAAACTCACGCCATCGCGGGAGTGTCGTTAACTTGGATCATGGCATTGGCTTGTGCTGTTCC
        Erythrolamprus_miliaris_Rh1   CCTGGCTGTAGAAAGATATGTAGTAGTTTGTAAGCCCATGAGCAACTTCCGTTTCACCGAAACTCACGCCATCGCGGGAGTGTCGTTAACTTGGATCATGGCATTGGCTTGTGCTGTTCC
     Erythrolamprus_poecilogyrus_Rh1   CCTGGCTGTAGAAAGATATGTAGTAGTTTGTAAGCCCATGAGCAACTTCCGTTTCACCGAAACTCACGCCATCGCGGGAGTGTCGTTAACTTGGATCATGGCATTGGCTTGTGCTGCTCC
               Helicops_modestus_Rh1   CCTGGCTGTAGAAAGATATGTAGTAGTTTGTAAGCCCATGAGCAACTTCCGTTTCACCGAAACTCACGCCATCATGGGAGTGTCCCTCACTTGGATCATGGCATTGGCTTGTGCTGCTCC
                Oxyrhopus_guibei_Rh1   CCTGGCTATAGAAAGATATGTAGTAGTTTGTAAGCCCATGAGCAACTTCCGTTTCACTGAAACTCACGCCATCGTGGGGTATCCTTCACTTGGATCATGGCTATGGCTTGTGCTGCTCC
         Philodryas_patagoniensis_Rh1   CCTGGCTGTAGAAAGATATGTAGTAGTTTGTAAGCCCATGAGCAACTTCCGTTTCACCGAAACTCACGCCATCATGGGAGTGTCCCTCACTTGGATCATGGCATTGGCTTGTGCTGCTCC
                   Python_regius_Rh1   CCTGGCTATAGAAAGATATGTAGTGGTTTGTAAGCCCATGAGCAATTTCCGTTTCACTGAGACTCATGCCATCATGGGACTGTGTTTCACATGGATCATGGCCTTGGCTTGTGCCGGTCC
           Sibynomorphus_mikanii_Rh1   CCTGGCTATAGAAAGATATGTAGTAGTTTGTAAGCCCATGAGCAACTTCCGTTTCACCGAAACTCACGCCATCATGGGAGTGGCCTTCACTTGGATCATGGCATTGGCTTGTGCTGCTCC
          Sibynomorphus_neuwiedi_Rh1   CCTGGCTATAGAAAGATATGTAGTAGTTTGTAAGCCCATGAGCAACTTCCGTTTCACCGAAACTCACGCCATCATGGGAGTGGCCTTCACTTGGATSATGGCATTGGCTTGTGCTGCTCC
               Spilotes_pullatus_Rh1   CCTGGCTGTAGAAAGATATGTAGTAGTTTGTAAGCCCATGAGTAACTTCCGTTTCACCGAAACTCATGCCATCATGGGAGTGTCTCTCACTTGGATCATGGCCTTGGCTTGTGCTGCGCC
         Taeniophallus_persimilis_Rh1   CCTGGCTGTAGAAAGATATGTAGTAGTTTGTAAGCCCATGAGCAACTTCCGTTTCACCGAAACTCACGCCATCATGGGAGTGTCCCTCACTTGGATCATGGCGTTGGCTTGTGCTGCTCC
         Thamnodynastes_strigatus_Rh1   CCTGGCTGTAGAAAGATATGTAGTAGTTTGTAAGCCCATGAGCAACTTCCGTTTCACCGAAATTCACGCCATTATGGGAGTGTCCCTCACTTGGATCATGGCATTGGCTTGTGCTGCTCC
                Tomodon_dorsatus_Rh1   CCTGGCTGTAGAAAGATATGTAGTAGTTTGTAAGCCCATGAGCAACTTCCGTTTCACGGAAACTCACGCCATCATGGGAGTGTCCCTCACTTGGATCATGGCGTTGGCTTGTGCTGCTCC
             Xenopeltis_unicolor_Rh1   CCTGGCTATAGAAAGATATGTAGTGGTTTGTAAGCCCATGAGTAATTTCCGTTTCACTGAAACTCATGCCATCATGGGACTGGCTTTCACTTGGATCATGGCCTTGTCTTGTGCTGGTCC
           Atractus_reticulatus_LWS   TATTTCCTGGGAGCGCTGGTTTGTTGTTTGCAAACCTTTTGGAAATATCAAGTTTGATGCTAAAATGGCTGTTGGTGGTATTTTGTTCTCCTGGATATGGGCTTCCTCCTGGACAGCACC
          Chironius_bicarinatus_LWS   TATTTCCTGGGAGCGCTGGGTTGTTGTTTGCAAACCTTTTGGAAATGTCAAGTTTGATGCTAAAATGGCTCTTGGTGGTATTTTCTTCTCCTGGATATGGTCTTGTGCCTGGACAGCACC
                 Dipsas_petersi_LWS   TATTTCCTGGGAGCGCTGGTTTGTTGTTTGCAAACCTTTTGGAAATATCAAGTTTGACGCTAAAATGGCTGTTGGTGGTATTTTGTTCTCCTGGATATGGGCTTCCTCTTGGACAGCACC
     Echinantera_cephalostriata_LWS   TATTTCCTGGGAGCGCTGGGTGGTTGTTTGCAAACCTTTTGGAAATGTCAAGTTTGATGCTAAAATGGCTATGGGTGGTATTTTGTTCTCCTGGATATGGTCTTGCGCCTGGACAGCACC
           Echinantera_undulata_LWS   TATTTCCTGGGAGCGCTGGGTGGTTGTTTGCAAACCTTTTGGAAATGTCAAGTTTGATGCTAAAATGGCTATGGGTGGTATTTTGTTCTCCTGGATATGGTCTTGCGCCTGGACAGCACC
      Erythrolamprus_aesculapii_LWS   TATTTCCTGGGAGCGCTGGGTTGTTGTTTGCAAACCTTTTGGAAATGTCAAGTTTGATGCTAAAATGGCTCTTGGTGGTATTTTTTTCTCCTGGATATGGTCTTGCGCCTGGACAGCACC
        Erythrolamprus_miliaris_LWS   TATTTCCTGGGAGCGCTGGGTTGTTGTTTGCAAACCTTTTGGAAATGTCAAGTTTGATGCTAAAATGGCTCTTGGTGGTATTTTTTTCTCCTGGATATGGTCTTGCGCCTGGACAGCACC
     Erythrolamprus_poecilogyrus_LWS   TATTTCCTGGGAGCGCTGGGTTGTTGTTTGCAAACCTTTTGGAAATGTCAAGTTTGATGCTAAAATGGCTGTTGGAGGTATTTTGTTTTCCTGGATATGGGCTTGTTCCTGGACAGCACC
               Helicops_modestus_LWS   TATTTCCTGGGAGCGCTGGGTTGTTGTTTGCAAACCTTTTGGCAATGTCAAGTTTGATGCTAAAATGGCTATTGGTGGTATTTTGTTCGCCTGGATATGGTCTTGTGCCTGGACAGCACC
                Oxyrhopus_guibei_LWS   TATTTCCTGGGAGCGCTGGGTTGTTGTTTGCAAACCTTTTGGAAATATCAAGTTTGATGCTAAAATGGCTCTTGGTGGTATTTTGTTCTCCTGGATATGGGCTTCCACCTGGACAGCACC
         Philodryas_patagoniensis_LWS   TATTTCCTGGGAGCGCTGGGTTGTTGTTTGCAAACCTTTTGGAAATGTCAAGTTTGATGCTAAAATGGCTCTTGGTGGTATTTTCTTCTCCTGGATATGGTCTGCCATCTGGACAGCACC
                   Python_regius_LWS   TATTTCCTGGGAGCGCTGGGTTGTGGTTTGCAAACCTTTTGGAAATCTCAAGTTTGATGCCAAACTGGCCCTTGCTGGCATTCTCTTCTCCTGGGTATGGTCTGCCACCTGGACAGCACC
           Sibynomorphus_mikanii_LWS   TATTTCCTGGGAGCGCTGGTTTGTTGTTTGCAAACCTTTTGGAAATATCAAGTTTGACGCTAAAATGGCTGTTGGTGGTATTTTGTTCTCCTGGATATGGGCTTCCTCTTGGACAGCACC
         Sibynomorphus_neuwiedii_LWS   TATTTCCTGGGAGCGCTGGTTTGTTGTTTGCAAACCTTTTGGAAATATCAAGTTTGACGCTAAAATGGCTGTTGGTGGTATTTTGTTTTCCTGGATATGGGCTTCCTCTTGGACAGCGCC
               Spilotes_pullatus_LWS   TATTTCCTGGGAGCGCTGGGTTGTTGTTTGCAAACCTTTTGGAAATGTCAAGTTTGATGCTAAAATGGCTCTTGGTGGTATTTTCTTCTCCTGGATATGGTCTTGCGTCTGGACAGCACC
         Taeniophallus_persimilis_LWS   TATTTCCTGGGAGCGCTGGGTTGTTGTTTGCAAACCTTTTGGAAATGTCAAGTTTGATGCTAAAATGGCTATGGGTGGTATTTTGTTCTCCTGGATATGGTCTTGCGCCTGGACAGCACC
         Thamnodynastes_strigatus_LWS   TATTTCCTGGGAGCGCTGGGTTGTTGTTTGCAAACCTTTTGGAAATGTCAAGTTTGATGCTAAAATGGCTATTGGTGGTATTTTGTTCGCCTGGATATGGGCTGGCGTCTGGACAGCACC
                Tomodon_dorsatus_LWS   TATTTCCTGGGAGCGCTGGGTTGTTGTTTGCAAACCTTTTGGAAATGTCAAGTTTGATGCTAAAATGGCTGTTGGTGGTATTTTGTTCGCCTGGATATGGGCTTGCACCTGGACAGCACC
             Xenopeltis_unicolor_LWS   AATTTCCTGGGAGCGCTGGGTTGTTGTTTGCAAACCTTTTGGAAATCTCAAGTTTGATGCCAAAATGGCCCTTGCTGGCATTCTCTTCTCCTGGGTATGGTCTGCCACCTGGACAGCACC
          Atractus_reticulatus_SWS1   CCTCGCCTTTGAGCGCTACATCGTGATCTGCAAGCCGCTGGGGAACTTCCGTTTCCATGCCAAACACGCCGTGGTAGTGGTGGCGACCACGTGGGTCATTGGAGTGGGGGTGGCGATCCC
         Chironius_bicarinatus_SWS1   CCTCGCCTTTGAACGCTACATCGTGATCTGCAAGCCGCTAGGGAACTTCCGTTTCAACTCCAAACACGCTCTGGTAGTGGTGGCGACCACGTGGATCATTGGAATCGCGGTCTCCGTTCC
                Dipsas_petersi_SWS1   CCTCGCCTTCGAGCGCTACATCGTGATCTGCAAGCCGCTGGGGAACTTCCGTTTCAACGCCAAACACGCCGTGGTAGTGGTGGCGACCACATGGGTCATCGGAGTGGGGGTAGCTATCCC
    Echinantera_cephalostriata_SWS1   CCTYGCCTTTGAACGCTACATCGTGATCTGCAAGCCGATGGGGAACTTCCGTTTCAACGCCAAACATGCTCTGGTAGTGGTGGCGACCACGTGGGTCATTGGAGTGGGGGTGGCTGTCCC
          Echinantera_undulata_SWS1   CCTCGCCTTTGAACGCTACATCGTGATCTGCAAGCCGATGGGGAACTTCCGTTTCAACGCCAAACATGCTCTGGTAGTGGTGGCGACCACGTGGGTCATTGGAGTGGGGGTGGCTGTCCC
     Erythrolamprus_aesculapii_SWS1   CCTCGCCTTCGAACGCTACATCGTGATCTGCAAGCCGCTGGGGAACTTCCGTTTCAATGCCAAACATGCTCTGGTAGTGGTGGCGACCACGTGGGTCATTGGAGTGGGGGTGGCTATCCC
       Erythrolamprus_miliaris_SWS1   CCTCGCCTTCGAACGCTACATCGTGATCTGCAAGCCGCTGGGGAACTTCCGTTTCAATGCCAAACATGCTCTGGTAGTGGTGGCGACCACGTGGGTCATTGGAGTGGGGGTGGCTATCCC
    Erythrolamprus_poecilogyrus_SWS1   CCTCGCCTTCGAACGCTACATCGTGATCTGCAAGCCGCTGGGGAACTTCCGTTTCAACGCCAAACATGCTCTGGTAGTGGTGGCGACCACGTGGGTCATTGGAGTGGGGGTGGCTATCCC
              Helicops_modestus_SWS1   CCTYGCCTTTGAACGCTACATCGTGATCTGCAAGCCAATKGGGAGCTTCCGTTTCAACGCTAAACATGCYATGGTAGTGGTGGCAAYCACGTGGCTCATTGGAGTGGGGGTGGCTCTCCC
               Oxyrhopus_guibei_SWS1   CCTCGCCTTTGAACGCTACATCGTGATCTGCAAGCCGCTGGGGAACTTCCGTTTCAACGCCAAACATGCTGTGGTAGTGGTGGCAACCACGTGGGTCATTGGAGTGGGGGTGGCTGTCCC
       Philodryas_patagoniensis_SWS1   CCTTGCCTTTGAACGCTACATCGTGATCTGCAAGCCRATGGGGAACTTCCGTTTCAAYGGCAAACATGCTCTGGTAGTGGTGGCGACCACGTGGGTCATTGGAGTGGGGGTGGCTCTCCC
                  Python_regius_SWS1   CCTTGCCTTCGAACGATACATCGTCATCTGCAAGCCCCTGGGGAATTTCCGCTTCAACTCCAAGCATGCTCTGCTGGTGGTGGTGGCCACGTGGGTCATCGGGGTCGGGGTGTCCGTCCC
          Sibynomorphus_mikanii_SWS1   CCTCGCCTTCGAGCGCTACATCGTGATCTGCAAGCCGCTCGGGAACTTCCGTTTCAACGCCAAACACGCCGTGGTAGTGGTGGCGACCACATGGGTCATCGGAGTGGGGGTAGCTATCCC
        Sibynomorphus_neuwiedii_SWS1   CCTCGCCTTCGAGCGCTACATCGTGATCTGCAAGCCGCTGGGGAACTTCCGTTTCAACGCCAAACACGCCGTGGTAGTGGTGGCAACCACATGGGTCATCGGAGTGGGGGTAGCTATCCC
              Spilotes_pullatus_SWS1   CCTCGCCTTCGAACGCTACATCGTGATCTGCAAGCCGCTGGGGAACTTCCGTTTCAACTCCAAACATGCTCTGGTAGTGGTGGCGACCACGTGGATCATTGGAATTGGGGTCTCCGTTCC
       Taeniophallus_persimilis_SWS1   CCTCGCCTTTGAACGCTACATCGTGATCTGCAAGCCGATGGGGAACTTCCGTTTCAACGCCAAACATGCTCTGGTAGTGGTGGCGACCACGTGGGTCATTGGAGTGGGGGTGGCTGTCCC
       Thamnodynastes_strigatus_SWS1   CCTCGCCTTTGAACGCTACATCGTGATCTGCAAGCCGATGGGGAACTTCCGCTTCAACGCCAAACATGCTGTGGTGGTGGTGGCGACCACGTGGGTCATTGGAGTGGGGGTGGCTATCCC
               Tomodon_dorsatus_SWS1   CCTCGCCTTTGAACGCTACATCGTGATCTGCAAGCCGATGGGGAACTTCCGCTTCAACGCCAAACATGCTGTGGTAGTGGTGGCGACCACGTGGATCATTGGAGTGGGGGTGGCTATCCC
            Xenopeltis_unicolor_SWS1   CCTCGCCTTCGAACGATATGTGGTCATCTGCAAACCCCTGGGAAATTTCCGCTTCAACGCCAAACACGCCCTGCTGGTGGTGGCGGCCACGTGGGTCATCGGGGTTGGGGTGTCCATCCC
```

```
                                   61 1     620       630       640       650       660       670       680       690       700       710       720       730
                    Bos_taurus_Rh1   CCCCCTCGTCGGCTGGTCCAGGTACATCCCGGAGGGCATGCAGTGCTCGTGCGGGATTGACTACTACACG------------CCCCACGAGGAGACCAACAATGAGTCGTTCGTCATCTA
           Atractus_reticulatus_Rh1   TCCTCTGGTTGGATGGTCAAGGTATATCCCAGAAGGTATGCAGAGCTCATGTGGAATTGATTATTATACG-------CCAA-CCCCAGAAG----TCTATAATGAGTCCTATGTCATCTA
          Chironius_bicarinatus_Rh1   TCCTCTGATTGGATGGTCAAGGTATATCCCAGAAGGTATGCAGAGCTCATGTGGAGTTGATTATTATACG-------CCAA-CCCCAGAAG----TCCATAATGAGTCCTTTGTCATCTA
                Dipsas_petersi_Rh1   TCCTCTGGTTGGATGGTCAAGGTATATCCCAGAAGGTATGCAGAGCTCATGTGGAATTGATTATTATACG-------CCAA-GCCCACAAG----TCTATAATGAGTCCTTTGTCATCTA
   Echinantera_cephalostriata_Rh1   TCCTCTRGTTGGATGGTCAAGGTATATCCCAGAAGGTATGCAGAGCTCATGTGGAGTTGATTATTATACA-------CCAA-CCCCAGAAG----TCTATAATGAGTCCTTTGTCATCTA
           Echinantera_undulata_Rh1   TCCTCTGGTTGGATGGTCAAGGTATATCCCAGAAGGTATGCAGAGCTCATGTGGAGTTGATTATTATACA-------CCAA-CCCCAGAAG----TCTATAATAAGTCCTTTGTCATCTA
      Erythrolamprus_aesculapii_Rh1   TCCTCTGATTGGATGGTCAAGGTATATCCCAGAAGGTATGCAGAGCTCGTGTGGAGTTGATTATTATACA-------CCAA-CCCCAGAAG----TCTATAATGAGTCTTTTGTCATCTA
        Erythrolamprus_miliaris_Rh1   TCCTCTGATTGGATGGTCAAGGTATATCCCAGAAGGTATGCAGAGCTCGTGTGGAGTTGATTATTATACA-------CCAA-CCCCAGAAG----TCTATAATGAGTCTTTTGTCATCTA
    Erythrolamprus_poecilogyrus_Rh1   TCCTCTGATTGGATGGTCAAGGTATATCCCAGAAGGTATGCAGAGCTCGTGTGGAGTTGATTATTATACA-------CCAA-CCCCAGAAG----TCTATAATGAGTCTTTTGTCATCTA
              Helicops_modestus_Rh1   TCCTTTGGTTGGATGGTCAAGGTATATCCCAGAAGGTATGCAGAGTTCATGTGGAGTTGATTATTATACA-------CCAA-CCCCAGAAG----TCTATAATGAGTCCTTTGTCATCTA
               Oxyrhopus_guibei_Rh1   TCCTCTGGTTGGATGGTCAAGGTATATCCCAGAAGGTATGCAGACCTCATGTGGAATTGATTATTATACA-------CCAT-CCCCAGAAG----TCTATAATGAGTCCTTTGTCATCTA
       Philodryas_patagoniensis_Rh1   TCCTCTGATTGGATGGTCAAGGTATATCCCAGAAGGTATGCAGAGCTCATGTGGAGTTGATTATTATACA-------CCAA-CCCCAGAAG----TCTATAATGAGTCCTTTGTCATCTA
                  Python_regius_Rh1   TCCTCTGGTTGGATGGTCAAGGTATATCCCAGAAGGTATGCAGTGCTCATGTGGAGTTGATTATTATACA-------CCAA-CCCCAGAAG----TCCATAATGAGTCCTTTGTCATCTA
          Sibynomorphus_mikanii_Rh1   TCCTCTAGTTGGATGGTCAAGGTATATCCCAGAAGGTATGCAGAGCTCATGTGGAATTGATTATTATACG-------CCAA-GCCCACAAG----TCTATAATGAGTCGTTTGTCATCTA
         Sibynomorphus_neuwiedi_Rh1   TCCTCTGGTTGGATGGTCAAGGTATATCCCAGAAGGTATGCAGAGCTCATGTGGAATTGATTATTATACG-------CCAA-GCCCACAAG----TCTATAATGAGTCCTTTGTCATCTA
             Spilotes_pullatus_Rh1   TCCTCTGATTGGATGGTCAAGGTATATTCCAGAAGGTATGCAGAGCTCATGTGGAGTTGATTATTATACG-------CCAA-CCCCAGAAG----TCCATAATGAGTCCTTTGTCATCTA
       Taeniophallus_persimilis_Rh1   TCCTCTGGTTGGATGGTCAAGGTATATCCCAGAAGGTATGCAGAGCTCGTGTGGAGTTGATTATTATACA-------CCAA-CCCCAGAAG----TCTATAATAAGTCCTTTGTCATCTA
      Thamnodynastes_strigatus_Rh1   TCCTCTGATTGGATGGTCAAGGTATATCCCAGAAGGTATGCAGAGTTCATGTGGAGTTGATTATTATACA-------CCAA-CCCCAGAAG----TCTATAATGAGTCCTTTGTCATCTA
              Tomodon_dorsatus_Rh1   TCCTCTGATTGGATGGTCAAGGTATATCCCAGAAGGTATGCAGAGTTCATGTGGAGTTGATTATTATACA-------CCAA-CCCCAGAAG----TCTATAATGAGTCCTTTGTCATCTA
            Xenopeltis_unicolor_Rh1   TCCTCTGGTTGGATGGTCAAGGTATATCCCAGAAGGTATGCAGTGCTCATGTGGAGTTGATTATTATACA-------CCAA-GTCCAGAAG----TCCAGAATGAATCCTTTGTCATCTA
           Atractus_reticulatus_LWS   ACCAATCTTTGGCTGGAGTAGATACTGGCCCCATGGTCTGAAAACTTCTTGTGGTCCAGAT--GTATTCAGTGGCAATGAAGATCCAGGTG----TCC------AGTCTTACATGCTTAC
          Chironius_bicarinatus_LWS   ACCAATCTTTGGCTGGAGTAGATACTGGCCGCATGGTCTGAAAACATCATGTGGTCCAGAT--GTATTCAGTGGCAGTGAAGATCCAGGTG----TCC------AGTCTTACATGGTTGT
                 Dipsas_petersi_LWS   ACCAATGTTTGGCTGGAGTAGATACTGGCCCCATGGTCTGAAAACTTCTTGTGGTCCAGAT--GTATTCAGTGGCAATGAAGATCCAGGTG----TCC------AGTCTTACATGCTTAC
    Echinantera_cephalostriata_LWS   RCCAATCTTTGGCTGGAGTAGATACTGGCCCCATGGTCTGAAAACATCATGTGGTCCAGAT--GTATTCAGTGGCAGTGAAGATCCAGGTG----TCC------AGTCTTACATGGTTGT
           Echinantera_undulata_LWS   GCCAATCTTTGGCTGGAGTAGATACTGGCCCCATGGTCTGAAAACATCATGTGGTCCAGAT--GTGTTCAGTGGCAGTGAAGATCCAGGTG----TCC------AGTCTTACATGGTTGT
      Erythrolamprus_aesculapii_LWS   ACCAGTCTTTGGCTGGAGTAGGTACTGGCCCCATGGCCTGAAAACATCATGTGGTCCAGAT--GTATTCAGTGGCAGTGAAGATCCAGGTG----TCC------AGTCTTACATGGTTGT
        Erythrolamprus_miliaris_LWS   ACCAGTCTTTGGCTGGAGTAGGTACTGGCCCCATGGCCTGAAAACRTCATGTGGTCCAGAT--GTATTCAGTGGCAGTGAAGATCCAGGTG----TCC------AGTCTTACATGGTTGT
    Erythrolamprus_poecilogyrus_LWS   ACCAATCTTTGGCTGGAGTAGGTACTGGCCCCATGGACTGAAAACATCATGTGGTCCAGAT--GTATTCAGTGGCAATGAAGATCCAGGTG----TCC------AGTCTTACATGATTGT
              Helicops_modestus_LWS   GCCAATCTTTGGCTGGAGTAGATATTGGCCCCATGGTCTGAAAACATCATGTGGTCCAGAT--GTATTCAGTGGCAATGAAGATCCAGGTG----TCC------AGTCTTACATGATTGT
               Oxyrhopus_guibei_LWS   GCCAATCTTCGGCTGGAGTAGATATTGGCCCCATGGTCTGAAAACATCATGTGGTCCGGAT--GTATTCAGTGGCAGTGAAGATACAGGGG----TCC------AGTCTTACATGATTGT
       Philodryas_patagoniensis_LWS   GCCAATCTTTGGCTGGAGTAGATACTGGCCCCATGGTCTGAAAACATCATGTGGTCCAGAT--GTATTCAGTGGCAATGAAGATCCAGGTG----TCC------AGTCTTACATGGTTGT
                  Python_regius_LWS   ACCAATCTTTGGTTGGAGTAGATACTGGCCCCATGGTCTCAAAACATCATGTGGTCCAGAT--GTCTTCAGTGGCAATGAAGATCCTGGTG----TCC------AGTCTTACATGATTGT
          Sibynomorphus_mikanii_LWS   ACCAATGTTTGGCTGGAGTAGATACTGGCCCCATGGTCTGAAAACTTCTTGTGGTCCAGAT--GTATTCAGTGGCAATGAAGATCCAGGTG----TCC------AGTCTTACATGCTTAC
        Sibynomorphus_neuwiedii_LWS   ACCAATGTTTGGCTGGAGTAGATACTGGCCCCATGGTCTGAAAACTTCTTGTGGTCCAGAT--GTATTCAGTGGCAATGACGATCCAGGTG----TCC------AGTCTTACATGCTTAC
              Spilotes_pullatus_LWS   ACCAATCTTTGGCTGGAGTAGATACTGGCCCCATGGTCTGAAAACGTCATGTGGTCCAGAT--GTATTCAGTGGCAATGAAGATCCAGGTG----TCC------AGTCTTACATGATTGT
       Taeniophallus_persimilis_LWS   GCCAATCTTTGGCTGGAGTAGATACTGGCCCCATGGTCTGAAAACATCATGTGGTCCAGAT--GTATTCAGTGGCAGTGAAGATCCAGGTG----TCC------AGTCTTACATGGTTGT
      Thamnodynastes_strigatus_LWS   GCCAATCTTTGGCTGGAGTAGATATTGGCCCCATGGTATAAAAACATCATGTGGTCCAGAT--GTATTCAGTGGCAATGAAGATCCAGGTG----TCC------AGTCTTACATGATTGT
              Tomodon_dorsatus_LWS   GCCAATCTTTGGCTGGAGTAGATATTGGCCCCATGGTATAAAAACATCATGTGGTCCAGAT--GTATTCAGTGGCAATGAAGATCCAGGTG----TCC------AGTCTTACATGATTGT
            Xenopeltis_unicolor_LWS   ACCAATCTTTGGCTGGAGTAGATACTGGCCCCATGGTCTCAAAACATCATGCGGTCCAGAT--GTCTTCAGTGGCAATGAAGATCCTGGCG----TCC------AATCTTACATGATTGT
          Atractus_reticulatus_SWS1   GCCCTTCTTCGGCTGGAGCAGGTTCATCCCGGAAGGGCTGCAATGTTCCTGCGGTCCCGACTGGTACACGGTGGGTACCAA-GTACAAGAG-----------CGAATATTACTCGTGGTT
         Chironius_bicarinatus_SWS1   CCCCTTCTTTGGCTGGAGCAGGTTCCTCCCGGAAGGGCTGCAGTGTTCCTGCGGTCCCGACTGGTACACGGTGGGTACCAA-ATACAAGAG-----------CGAGTATTACTCGTGGTT
                Dipsas_petersi_SWS1   CCCCTTCTTCGGCTGGAGCAGGTTCATCCCGGAAGGGCTGCAGTGTTCCTGCGGTCCCGACTGGTACACAGTGGGTACCAA-GTACAAGAG-----------CGAATACTACTCGTGGTT
   Echinantera_cephalostriata_SWS1   CCCCTTCTTCGGCTGGAGCAGGTTCCTCCCAGAAGGGCTGCAGTGTTCCTGCGGTCCCGATTGGTACACGGTGGGTACCAA-ATACAAGAG-----------CGAATATTACTCRTGGTT
          Echinantera_undulata_SWS1   CCCCTTCTTCGGCTGGAGCAGGTTCCTCCCGGAAGGGCTGCAGTGTTCCTGCGGTCCCGACTGGTATACGGTGGGTACCAA-ATACAAGAG-----------CGAATATTACTCGTGGTT
     Erythrolamprus_aesculapii_SWS1   CCCCTTCTTCGGCTGGAGCAGGTTCCTCCCGGAAGGGCTGCAGTGTTCCTGCGGTCCCGACTGGTACACGGTGGGTACCAA-ATACAAGAG-----------CGAATATTACTCGTGGTT
       Erythrolamprus_miliaris_SWS1   CCCCTTCTTCGGCTGGAGCAGGTTCCTCCCGGAAGGGCTGCAGTGTTCCTGCGGTCCCGACTGGTACACGGTGGGTACCAA-ATACAAGAG-----------CGAATATTACTCGTGGTT
   Erythrolamprus_poecilogyrus_SWS1   CCCCTTCTTCGGCTGGAGCAGGTTCCTCCCGGAAGGGCTGCAGTGTTCCTGCGGTCCCGACTGGTACACGGTGGGTACCAA-ATACARGAG-----------CGAATATTACTCATGGTT
             Helicops_modestus_SWS1   CCCCTTCTTCGGCTGGAGCAGGTTCGTCCCGGAAGGGCTGCAATGTTCCTGCGGTCCCGACTGGTACACGGTGGGTACCAA-ATACAAGAG-----------CGAATATTACTCGTGGTT
              Oxyrhopus_guibei_SWS1   CCCCTTCTTCGGCTGGAGCAGGTTCCTCCCGGAAGGGCTGCAGTGTTCCTGCGGTCCCGACTGGTACACGGTGGGTACCAA-ATACAAGAG-----------CGAATATTACTCGTGGTT
      Philodryas_patagoniensis_SWS1   CCCTTTCTTCGGCTGGAGCAGGTTCCTYCCAGAAGGGCTGCAGTGTTCCTGCGGTCCCGACTGGTACACGGTGGGTACCAA-ATACAAGAG-----------CGAATATTACTCGTGGTT
                 Python_regius_SWS1   CCCCTTCTTTGGCTGGAGCAGGTTCATCCCGGAAGGGCTGCAGTGTTCCTGCGGTCCCGACTGGTACACGGTGGGCACCAA-GTACAAGAG-----------CGAGTATTACTCGTGGTT
         Sibynomorphus_mikanii_SWS1   CCCCTTCTTCGGCTGGAGCAGGTTCATCCCGGAAGGGCTGCAGTGTTCCTGTGGTCCCGACTGGTACACGGTGGGTACCAA-GTACAAGAG-----------CGAATACTACTCCTGGTT
       Sibynomorphus_neuwiedii_SWS1   CCCCTTCTTCGGCTGGAGCAGGTTCATCCCGGAAGGGCTGCAGTGTTCCTGCGGTCCCGACTGGTACACGGTGGGTACCAA-GTACAAGAG-----------CGAATACTACTCGTGGTT
             Spilotes_pullatus_SWS1   CCCCTTCTTTGGCTGGAGCAGGTTCCTCCCAGAAGGGCTGCAGTGTTCCTGCGGTCCCGACTGGTACACAGTGGGTACCAA-ATACAAGAG-----------CGAGTATTACTCGTGGTT
      Taeniophallus_persimilis_SWS1   CCCCTTCTTCGGCTGGAGCAGGTTCCTCCCGGAAGGGCTGCAGTGTTCCTGCGGTCCCGACTGGTACACGGTGGGTACCAA-ATACAAGAG-----------CGAATAYTACTCGTGGTT
      Thamnodynastes_strigatus_SWS1   CCCCTTCTTCGGTTGGAGCAGGTTCCTCCCGGAAGGGCTGCAGTGTTCCTGCGGCCCCGACTGGTACACGGTGGGTACCAA-ATACAAGAG-----------TGAATATTACTCGTGGTT
             Tomodon_dorsatus_SWS1   CCCCTTCTTCGGCTGGAGCAGGTTCCTCCCRGAAGGGCTGCAGTGTTCCTGCGGCCCCGACTGGTACACRGTGGGTACCAA-ATACAAGAG-----------CGAGTATTACTCGTGGTT
           Xenopeltis_unicolor_SWS1   GCCCTTCTTTGGCTGGAGCAGGTTCATCCCAGAAGGCCTGCAATGTTCCTGTGGCCCCGACTGGTACACGGTGGGCACCAA-GTACAAGAG-----------CGAGTATTACTCGTGGTT
```

```
                                   731      740       750       760       770       780       790       800       810       820       830       840       850
                                   |        |         |         |         |         |         |         |         |         |         |         |         |
Bos_taurus_Rh1                     CATGTTCGTGGTCCACTTCATCATCCCCCTGATTGTCATATTCTTCTGCTACGGGCAGCTGGTGTTCACCGTCAAGGAGGCGGCTGCCCAGCAGCAGGAGTCGGCCACCACTCAGAAGGC
Atractus_reticulatus_Rh1           TATGTTCCTTGTACACTTTATCATTCCATTGATGGTTATCTTCTTCTGCTATGGTCGTCTTCTCTGCACTGTTAAAGAGGCCGCTGCTCAGCAACAGGAATCAGCCACCACCCAGAAGGC
Chironius_bicarinatus_Rh1          TATGTTCCTTGTACACTTTGTCACTCCATTGACGGTTATCTTCTTCTGCTATGGTCGTCTTGTCTGTACTGTTAAAGAGGCCGCAGCTCAGCAACAGGAGTCAGCCACCACCCAGAAGGC
Dipsas_petersi_Rh1                 TATGTTCATTGTACACTTTCTCATTCCTTTGACGGTTATCTTCTTCTGCTATGGTCGTCTTCTCTGCACTGTTAAAGAGGCCGCTGCTCAGCAACAGGAATCAGCCACTACCCAGAAGGC
Echinantera_cephalostriata_Rh1     TATGTTCCTTGTACACTTTGTCACTCCATTGACGATTATCTTCTTCTGCTATGGTCGTCTTCTCTGCGCTGTTAAAGAGGCTGCTGCTCAGCAACAGGAATCAGCCACCACCCAGAAGGC
Echinantera_undulata_Rh1           TATGTTCCTTGTACACTTTGTCACTCCATTGACGATTATCTTCTTCTGCTATGGTCGTCTTCTCTGCACTGTTAAAGAGGCTGCTGCTCAGCAACAGGAATCAGCCACCACCCAGAAGGC
Erythrolamprus_aesculapii_Rh1      CATGTTCCTTGTGCACTTTGTCACTCCATTGACGGTTATCTTCTTCTGCTATGGTCGTCTTCTCTGCACTGTTAAAGAGGCTGCTGCTCAGCAACAGGAATCAGCCACCACCCAGAAGGC
Erythrolamprus_miliaris_Rh1        CATGTTCCTTGTACACTTTGTCACTCCRTTGACGGTTATCTTCTTCTGCTATGGTCGTCTTCTCTGCACTGTTAAAGAGGCTGCTGCTCAGCAACAGGAATCAGCCACCACCCAGAAGGC
Erythrolamprus_poecilogyrus_Rh1    CATGTTCCTGGTACACTTTGTCACTCCATTGACGGTTATCTTCTTCTGCTATGGTCGTCTTCTCTGCACTGTTAAAGAGGCTGCTGCTCAGCAACAGGAATCAGCCACCACCCAGAAGGC
Helicops_modestus_Rh1              TATGTTCCTTGTACACTTTGTCACTCCATTGACGGTTATATTCTTCTGCTATGGTCGTCTTCTCTGCRCTGTTAAAGAGGCTGCTGCTCAGCAACAGGAATCAGCCACCACCCAGAAGGC
Oxyrhopus_guibei_Rh1               TATGTTCGTTGTACACTTTATGATTCCATTGATTGTTATCTTCTTCTGCTATGGTCGTCTTCTCTGTGCTGTTAAAGAGGCTGCTGCTCAGCAACAGGAATCAGCCACCACCCAGAAGGC
Philodryas_patagoniensis_Rh1       TATGTTCCTTGTACACTTTGTCACCCCATTGACGGTTATCTTCTTCTGCTATGGTCGTCTTCTCTGCACTGTTAAAGAGGCTGCTGCTCAGCAACAGGAATCAGCCACCACCCAGAAGGC
Python_regius_Rh1                  TATGTTTATTGTACATTTTGTCATTCCATTGGTGGTTATCTTTTTCTGCTATGGACGTCTTGTCTGCACTGTTAAAGAGGCTGCTGCTCAGCAACAGGAATCAGCCACCACCCAGAAGGC
Sibynomorphus_mikanii_Rh1          TATGTTCGTTGTACACTTTCTCATTCCATTGACGGTTATCTTCTTCTGCTATGGTCGTCTTCTCTGCACTGTTAAAGAGGCCGCTGCTCAGCAACAGGAATCAGCCACTACCCAGAAGGC
Sibynomorphus_neuwiedi_Rh1         TATGTTCGTTGTACACTTTCTCATTCCATTGACGGTTATCTTCTTCTGCTATGGTCGTCTTCTCTGCACTGTTAAAGAGGCCGCTGCTCAGCAACAGGAATCAGCCACTACCCAGAAGGC
Spilotes_pullatus_Rh1              TATGTTCCTTGTACACTTTGTCACTCCATTGACCGTTATCTTCTTCTGCTACGGTCGTCTTGTCTGCACTGTTAAAGAGGCTGCTGCTCAGCAACAGGAGTCAGCCACCACCCAGAAGGC
Taeniophallus_persimilis_Rh1       TATGTTCCTTGTACACTTTGTCACTCCATTGACGATTATCTTCTTCTGCTATGGTCGTCTTCTCTGCACTGTTAAAGAGGCTGCTGCTCAGCAACAAGAATCAGCCACCACCCAGAAGGC
Thamnodynastes_strigatus_Rh1       TATGTTCCTTGTACACTTTGTCACTCCATTGACGATTATCTTCTTCTGCTATGGTCGTCTTCTCTGCACTGTTAAAGAGGCTGCTGCTCAGCAACAGGAATCAGCCACCACTCAGAAGGC
Tomodon_dorsatus_Rh1               TATGTTCCTTGTACACTTTGTCACTCCATTGACGGTTATCTTCTTCTGCTATGGTCGTCTTCTCTGCACTGTTAAAGAGGCTGCTGCTCAGCAACAGGAATCAGCCACCACTCAGAAGGC
Xenopeltis_unicolor_Rh1            TATGTTCATTGTACACTTTATCATTCCAATGATAGTTATCTCCTTCTGCTACGGACGTCTTGTCTGCACTGTTAAAGAGGCTGCTGCTCAGCAACAGGAATCAGCCACCACCCAGAAGGC
Atractus_reticulatus_LWS           CCTGATGATTACCTGTTGTGTAATTCCCCTGAGCATCATCATTCTCTGTTACTTGCAAGTGTGGATGGCTATCCGTGCGGTTGCAGCCCAGCAGAAAGAGTCAGAATCAACACAGAAGGC
Chironius_bicarinatus_LWS          CCTGATGATTACTTGTTGTGCAATTCCCCTGAGCGTCATCATTATCTGTTACTTGCAAGTGTGGATGGCTATCCGTGCGGTTGCAGCCCAGCAAAAAGAGTCAGAATCAACACAGAAGGC
Dipsas_petersi_LWS                 CCTGATGATTACCTGTTGTATAATTCCCCTGAGCATCATCATTCTCTGTTACTTGCAAGTGTGGATGGCTATCCGTGCGGTTGCAGCCCAGCAGAAAGAGTCAGAATCAACACAGAAGGC
Echinantera_cephalostriata_LWS     CCTGATGATTACCTGTTGTGCAATTCCCCTGAGCGTCATCATTATCTGTTACTTGCAAGTGTGGCTGGCTATCCGTGCGGTTGCAGCCCAGCAGAAAGAGTCAGAATCAACACAGAAGGC
Echinantera_undulata_LWS           CCTGATGATTACCTGTTGTGCAATTCCCCTGAGTGTCATCATTATCTGTTACTTGCAAGTGTGGCTGGCTATCCGTGCGGTTGCAGCCCAGCAGAAAGAGTCAGAATCAACACAGAAGGC
Erythrolamprus_aesculapii_LWS      CCTGATGATTACCTGTTGTGCAATTCCCCTGAGCGTCATCGTTATCTGCTACTTGCAAGTGTGGCTGGCTATCCGTGCGGTTGCAGCCCAGCAGAAAGAGTCAGAATCAACACAGAAGGC
Erythrolamprus_miliaris_LWS        CCTGATGATTACCTGTTGTGCAATTCCCCTGAGCGTCATCGTTATCTGCTACTTGCAAGTGTGGATGGCTATCCGTGCGGTTGCAGCCCAGCAGAAAGAGTCAGAATCAACACAGAAGGC
Erythrolamprus_poecilogyrus_LWS    CCTGATGACTACCTGTTGTATAATTCCCCTGAGCGTCATCATTATCTGCTACTTGCAAGTGTGGATGGCTATCCGTGCGGTTGCAGCCCAGCAGAAAGAGTCAGAATCAACACAGAAGGC
Helicops_modestus_LWS              CCTGATGTCTACTTGTTGTATATTTCCCCTAAGCGTTATCATTATCTGTTACTTGCAAGTGTGGCTGGCTATTCGTGCGGTTGCAGCCCAGCAGAAAGAGTCAGAATCAACACAGAAGGC
Oxyrhopus_guibei_LWS               CCTGATGATTACCTGTTGTATAATTCCACTGAGCATAATTATTATCTGTTACTTGCAAGTGTGGCTGGCTATCCGTGCGGTTGCGGCCCAGCAGAAAGAGTCGGAATCAACCCAGAAGGC
Philodryas_patagoniensis_LWS       CCTGATGATTACCTGTTGTGCAATTCCCCTGAGCGTCATCATTATCTGTTACTTGCAAGTGTGGCTGGCTATCCGTGCGGTTGCAGCCCAGCAGAAAGAGTCAGAATCAACACAGAAGGC
Python_regius_LWS                  CCTGATGGTCACCTGTTGCATAATCCCCCTGAGCATCATTGTCCTCTGCTACATGCAAGTGTGGATGGCCATCCGTGCGGTTGCAGCCCAGCAGAAAGAGTCAGAATCGACACAGAAGGC
Sibynomorphus_mikanii_LWS          CCTGATGATTACCTGTTGTATAATTCCCCTGAGCATCATCATTCTCTGTTACTTGCAAGTGTGGATGGCTATCCGTGCGGTTGCAGCCCAGCAGAAAGAGTCAGAATCAACACAGAAGGC
Sibynomorphus_neuwiedii_LWS        CCTGATGATTACCTGTTGTATAATTCCCCTGAGCATCATCATTCTCTGTTACTTGCAAGTGTGGATGGCTATCCGTGCGGTTGCAGCCCAGCAGAAAGAGTCAGAATCAACACAGAAGGC
Spilotes_pullatus_LWS              CCTGATGATTACTTGTTGTGCAATTCCCCTGAGCGTCATCATTATCTGTTACTTGCAAGTGTGGATGGCTATCCGTGCGGTTGCAGCCCAGCAGAAAGAATCAGAATCAACACAGAAGGC
Taeniophallus_persimilis_LWS       CCTGATGATTACCTGTTGTGCAATTCCCCTGAGCGTCATCATTATCTGTTACTTGCAAGTGTGGCTGGCTATCCGTGCGGTTGCAGCCCAGCAGAAAGAGTCAGAATCAACACAGAAGGC
Thamnodynastes_strigatus_LWS       CCTGATGAGTACCTGTTGTATATTTCCCCTGAGCGTTATCATTCTCTGTTACATACAAGTGTGGCTGGCTATCCGTGCGGTTGCAGCCCAGCAGAAAGAGTCAGAATCAACACAGAAGGC
Tomodon_dorsatus_LWS               CCTGATGAGTACCTGTTGTATATTTCCCCTGAGCGTTATCATTATCTGTTACATACAAGTGTGGCTGGCTATCCGTGCGGTTGCAGCCCAGCAGAAAGAGTCAGAATCAACACAGAAGGC
Xenopeltis_unicolor_LWS            CCTGATGATCACCTGTTGCGTAATTCCCCTAAGCATCATCATCCTCTGCTACTTGCAAGTGTGGATGGCTATCCGTGCGGTTGCAGCCCAGCAGAAAGAGTCAGAATCAACACAGAAGGC
Atractus_reticulatus_SWS1          CCTTTTCATCTTCTGCTTCATCATACCACTGACCCTCATCATCTTCTCCTACGCGCGGCTCCTAGGTGCCCTCCGGGCGGTAGCCGCTCAGCAGCAAGAGTCAGCCACTACCCAGAAGGC
Chironius_bicarinatus_SWS1         CCTCTTCGTCTTCTGCTTCTTCATACCGTTCAGCCTCATCATCTACTCCTACGGGAGGCTCCTGAGTGCCCTCCGGGCGGTAGCCGCTCAGCAGCAAGAGTCGGCCACCACCCAGAAGGC
Dipsas_petersi_SWS1                CCTTTTTATCTTCTGCTTCATCATACCGCTGACCCTCATCATCTTCTCCTACGCGCAACTCCTGGGTGCCCTCCGAGCGGTAGCCGCTCAGCAGCAAGAGTCAGCCACTACCCAGAAGGC
Echinantera_cephalostriata_SWS1    CCTTTTCATCTTCTGCTTCCTCATACCGCTGACCCTCATCATCTTCTCCTACGCGCGGYTACTGGGTGCSCTCCGGGCGGTAGCCGCTCAGCAGCAAGAATCAGCCACCACCCAGAAGGC
Echinantera_undulata_SWS1          CCTTTTCATCTTCTGCTTCCTCATACCGCTGACCCTCATCATCTTCTCCTACGCGCGGCTACTGGGTGCCCTCCGGGCGGTAGCCGCTCAGCAGCAAGAATCAGCCACCACCCAGAAGGC
Erythrolamprus_aesculapii_SWS1     CCTTTTCATCTTCTGCTTCCTCATACCGCTGACCCTCATCATCTTCTCCTATGCGCGGCTACTAGGTGCCCTCCGGGCGGTAGCCGCTCAGCAGCAAGAATCAGCCACCACCCAGAAGGC
Erythrolamprus_miliaris_SWS1       CCTTTTCATCTTCTGTTTCCTCATACCGCTGACCCTCATCATCTTCTCCTACGCGCGGCTACTGGGTGCCCTCCGGGCGGTAGCCGCTCAGCAGCAAGAATCAGCCACCACCCAGAAGGC
Erythrolamprus_poecilogyrus_SWS1   CCTTTTCATCTTCTGCTTCCTCATACCGCTGACCCTCATCATCTTCTCCTACGCACGGCTACTGGGTGCCCTCCGGGCAGTAGCCGCTCAGCAGCAAGAATCAGCCACCACCCAGAAGGC
Helicops_modestus_SWS1             CCTTTTCGTCTTCTGCTTCCTCATACCGCTGRGCCTCATCATCTTCTCCTACGCGCGGCTACTGGGTGCCCTCCGGGCGGTGGCCGCTCAGCAGCAAGAATCAGCCACCACCCAGAAGGC
Oxyrhopus_guibei_SWS1              CCTTTTCATCTTCTGCTTCCTCATACCGCTGACCCTCATCATCTTCTCCTACGCGCAGCTACTGGGTGCCCTCCGGGCGGTAGCCGCTCAGCAGCAAGAATCAGCCACCACCCAGAAGGC
Philodryas_patagoniensis_SWS1      CCTTTTCATCTTCTGCTTCCTCATACCGCTGACCCTCATCATCTTCTCCTACGCGCGGCTACTGGGTGCCCTCCGGGCGGTAGCCGCTCAGCAGCAAGAATCAGCCACCACCCAGAAGGC
Python_regius_SWS1                 CCTCTTCGTCTTCTGCTTCATCGTGCCCCTCAGCCTCATCGTCTTCTCCTACCTGCGGCTCCTGGGTGCCCTCCGGGCGGTAGCCGCCCAGCAGCAGGAGTCAGCCACCACCCAGAAGGC
Sibynomorphus_mikanii_SWS1         CCTTTTCGTCTTCTGCTTCATCATACCGCTGACCCTCATCATCTTCTCCTACGCGCGGCTGCTGGGTGCCCTCCGAGCGGTAGCCGCTCAGCAGCAAGAGTCAGCCACTACCCAGAAGGC
Sibynomorphus_neuwiedii_SWS1       CCTTTTCATCTTCTGCTTCATCATACCGCTGACCCTCATCATCTTCTCCTACGCGCGGCTCCTGGGTGCCCTCCGAGCGGTAGCCGCTCAGCAGCAAGAGTCAGCTACTACCCAGAAGGC
Spilotes_pullatus_SWS1             CCTCTTCATCTTCTGCTTCCTCATACCGCTCACCCTCATCATCTACTCCTACGCGCGGCTCCTGGGTGCCCTCCGGGCGGTAGCCGCTCAGCAGCAAGAGTCGGCCACCACCCAGAAGGC
Taeniophallus_persimilis_SWS1      CCTTTTCATCTTCTGCTTCCTCATACCGCTGACCCTCATCATCTTCTCCTACGCRCGGCTACTGGGTGCSCTCCGGGCGGTAGCCGCTCAGCAGCAAGAATCAGCCACCACCCAGAAGGC
Thamnodynastes_strigatus_SWS1      CCTTTTCATCTTCTGCTTCCTCATACCGCTGACCCTCATCATCTTCTCCTATGCGCAACTACTGGGTGCCCTCCGGGCGGTAGCCGCTCAGCAGCAAGAATCAGCCACCACCCAGAAGGC
Tomodon_dorsatus_SWS1              CCTTTTCATCTTCTGCTTCCTCATACCGCTGACCCTCATCATCTTCTCCTACGCGCAACTACTGGGTGCCCTCCGGGCGGTAGCCGCTCAGCAGCAAGAATCAGCCACCACCCAGAAGGC
Xenopeltis_unicolor_SWS1           CCTCTTCGTCTTCTGCTTCATTGTGCCCCTCAGCCTCATCGTCTTCTCCTACCTGCAGCTGCTGCGTGCCCTCCGGGCGGTAGCTGCTCAGCAGCAGGAGTCAGCCACCACCCAGAAGGC
```

```
                                 851       860       870       880       890       900       910       920       930       940       950       1000      1010
                                 |         |         |         |         |         |         |         |         |         |         |         |         |
Bos_taurus_Rh1                   CGAGAAGGAGGTCACCCGCATGGTGATCATCATGGTCATCGCTTTCCTAATCTGCTGGCTGCCCTACGCTGGGGTGGCGTTCTACATCTTCACCCATCAGGGCTCTGACTTTGGCCCCAT
Atractus_reticulatus_Rh1         TGAGAAAGAAGTCACTCGCATGGTCATCCTTATGGTGATTGCCTTCCTTGTTTGCTGGGTTCCTTATGCCTCTGTGGCTTTCTATATCTTTACTCATCAAGGATCTGACTTTGGCCCTGT
Chironius_bicarinatus_Rh1        CGAGAAAGAAGTCACTCGCATGGTCATCCTTATGGTGATTGCCTTCCTTGTTTGCTGGGTCCCTTACGCCTCTGTGGCTTTCTATATCTTTACTCATCAAGGATCTGACTTTGGCCCTGT
Dipsas_petersi_Rh1               TGAGAAAGAAGTCACTCGCATGGTCATCCTTATGGTGATTGCCTTCCTTGTTTGCTGGGTCCCTTATGCCTCTGTGGCTTTCTATATCTTTACTCATCAAGGATCTGACTTTGGCCCTGT
Echinantera_cephalostriata_Rh1   TGAGAAAGAAGTCACTCGCATGGTCATCCTTATGGTGATTGCCTTCCTTGTTTGCTGGGTCCCTTATGCCTCTGTGGCTTTCTATATCTTTACTCATCAAGGATCTGACTTTGGCCCTGT
Echinantera_undulata_Rh1         TGAGAAAGAAGTCACTCGCATGGTCATCCTTATGGTGATTGCCTTCCTTGTTTGCTGGGTCCCTTATGCCTCTGTGGCTTTCTATATCTTTACTCATCAAGGATCTGACTTTGGCCCTGT
Erythrolamprus_aesculapii_Rh1    TGAGAAAGAAGTCACTCGCATGGTCATCCTTATGGTGATTGCCTTCCTTATTTGCTGGGTCCCTTATGCCTCTGTGGCTTTCTATATCTTCACTCATCAAGGATCTGACTTTGGCCCTGT
Erythrolamprus_miliaris_Rh1      TGAGAAAGAGGTCACTCGCATGGTCATCCTTATGGTGATTGCCTTCCTTATTTGCTGGGTCCCTTATGCCTCTGTGGCTTTCTATATCTTCACTCATCAAGGATCTGACTTTGGCCCTGT
Erythrolamprus_poecilogyrus_Rh1  TGAGAAAGAAGTCACTCGCATGGTCATCCTTATGGTGATTGCCTTCCTTATTTGCTGGGTCCCTTATGCCTCTGTGGCTTTCTATATCTTCACTCATCAAGGATCTGACTTTGGCCCTGT
Helicops_modestus_Rh1            TGAGAAAGAAGTCACTCGCATGGTCATCCTTATGGTGATTGCCTTCCTTGTTTGCTGGGTCCCTTATGCCTCTGTGGCGTTCTATATCTTTACTCATCAAGGATCCGACTTTGGCCCTGT
Oxyrhopus_guibei_Rh1             TGAGAAAGAAGTCACTCGCATGGTCATCCTTATGGTGATTGCCTTCCTTGTTTGCTGGGTCCCTTATGCCTCTGTGGCTTTCTATATCTTTACTCATCAAGGATCTGACTTTGGCCCTGT
Philodryas_patagoniensis_Rh1     TGAGAAAGAAGTCACTCGCATGGTCATCCTTATGGTGATTGCCTTCCTTGTTTGCTGGGTYCCTTATGCCTCTGTGGCTTTCTATATCTTTACTCATCAAGGATCTGACTTTGGCCCTGT
Python_regius_Rh1                TGAGAAAGAAGTCACTCGCATGGTCATCATCATGGTGATTGCCTTCCTGATTTGCTGGGTCCCTTATGCCAGTGTGGCTTTCTATATCTTTACTCATCAAGGATCTGACTTTGGCCCTGT
Sibynomorphus_mikanii_Rh1        TGAGAAAGAAGTCACTCGCATGGTCATCCTTATGGTGATTGCCTTCCTTGTTTGCTGGGTCCCTTATGCCTCTGTGGCTTTCTATATCTTTACTCATCAAGGATCTGACTTTGGCCCTGT
Sibynomorphus_neuwiedi_Rh1       TGAGAAAGAAGTCACTCGCATGGTCATCCTTATGGTGATTGCCTTCCTTGTTTGCTGGGTCCCTTATGCCTCTGTGGCTTTCTATATCTTTACTCATCAAGGATCTGACTTTGGCCCTGT
Spilotes_pullatus_Rh1            TGAGAAAGAAGTCACTCGCATGGTCATCCTTATGGTGGTTGCCTTCCTTGTTTGCTGGGTCCCTTATGCCTCTGTGGCTTTCTATATCTTTACTCATCAAGGATCTGACTTTGGCCCTGT
Taeniophallus_persimilis_Rh1     TGAGAAAGAAGTCACTCGCATGGTCATCCTTATGGTGATTGCCTTCCTTGTTTGCTGGGTCCCTTATGCCTCTGTGGCTTTCTATATCTTTACTCATCAAGGATCTGACTTTGGCCCTGT
Thamnodynastes_strigatus_Rh1     TGAGAAAGAAGTCACTCGCATGGTCATCCTTATGGTGATTGCCTTCCTTGTTTGTTGGGTCCCTTATGCCTCTGTGGCTTTCTATATCTTTACTCATCAAGGATCTGACTTTGGCCCTGT
Tomodon_dorsatus_Rh1             TGAGAAAGAAGTCACTCGCATGGTCATCCTTATGGTGATTGCCTTCCTTATTTGTTGGGTCCCTTATGCCTCTGTGGCTTTCTATATTTTTACTCATCAAGGATCTGACTTTGGCCCTGT
Xenopeltis_unicolor_Rh1          TGAGAAAGAAGTCACTCGCATGGTCGTCATCATGGTGATCGCCTTCCTTATTTGCTGGGTCCCTTATGCCAGTGTGGCTTTCTACATCTTTACTCATCAAGGATCTGACTTTGGCCCTGT
Atractus_reticulatus_LWS         TGAGAAGGAAGTGTCAAGAATGGTAGTGGTCATGATCATTGCCTATATTATCTGCTGGGGACCATATGCATCTTTTGCCTGTTTTGCTGCTGCCAATCCAGGCTACGCCTTTCACCCCCT
Chironius_bicarinatus_LWS        TGAGAAGGAAGTATCAAGGATGGTAGTRGTCATGATCATTGCCTATATTGTCTGCTGGGGACCATATACATTTTTTGCCTGTTTTGCTGTTGCCAATCCAGGCTATGCCTTTCACCCCCT
Dipsas_petersi_LWS               TGAGAAGGAAGTATCAAGGATGGTAGTGGTCATGATCATTGCCTATATTATCTGCTGGGGACCATATGCATCTTTTGCCTGTTTTGCTGCTGCCAATCCAGGCTACGCCTTTCACCCCCT
Echinantera_cephalostriata_LWS   TGAGAAGGAAGTATCAAGGATGGTAGTGGTCATGATCATTGCCTATATTGTCTGCTGGGGACCATATACATTTTTTGCCTGTTTTGCTGTTGCCAATCCAGGCTATGCCTTTCACCCCCT
Echinantera_undulata_LWS         TGAGAAGGAAGTATCAAGGATGGTAGTGGTCATGATCATTGCCTATATTGTCTGCTGGGGACCATATACATTTTTTGCCTGTTTTGCTGTTGCCAATCCAGGCTATGCCTTTCACCCCCT
Erythrolamprus_aesculapii_LWS    TGAGAAGGAAGTATCAAGGATGGTAGTGGTCATGATCATTGCCTATATTATCTGCTGGGGACCATATACATTTTTTGCCTGTTTTGCTGTTGCCAATCCAGGCTATGCCTTTCACCCTCT
Erythrolamprus_miliaris_LWS      TGAGAAGGAAGTATCAAGGATGGTAGTGGTCATGATCATTGCCTATATTGTCTGCTGGGGACCATATACATTTTTTGCCTGTTTTGCTGTTGCCAATCCAGGCTATGCCTTTCACCCTCT
Erythrolamprus_poecilogyrus_LWS  TGAGAAGGAAGTATCAAGGATGGTAGTGGTCATGATCATTGCCTATATTGTCTGCTGGGGACCATATACATTTTTTGCCTGTTTTGCTGTTGCCAATCCAGGCTATGCCTTTCACCCTCT
Helicops_modestus_LWS            TGAGAAGGAAGTATCAAGGATGGTAGTGGTCATGATCATTGCCTATATTGTCTGCTGGGGACCATATACATTTTTTGCCTGTTTTGCTGCTGCCAATCCAGGCTATGCCTTCCACCCCCT
Oxyrhopus_guibei_LWS             TGAGAAGGAAGTATCAAGGATGGTAGTGGTCATGATCATTGCCTACATTGTCTGCTGGGGACCATATACATTTTTTGCCTGTTTTGCCGTTGCCAATCCAGGCTATGCCTTCCACCCACT
Philodryas_patagoniensis_LWS     TGAGAAGGAAGTATCAAGGATGGTAGTGGTCATGATCATTGCCTATATTGTCTGCTGGGGACCATATACATTTTTTGCCTGTTTTGCTGTTGCCAATCCAGGCTATGCCTTTCACCCCCT
Python_regius_LWS                TGAGAAGGAAGTATCAAGGATGGTAGTGGTCATGATCCTTGCCTATATTTTCTGCTGGGGACCATATACAACTTTTGCCTGCTTTGCTGCTGCCAATCCAGGCTATGCCTTTCACCCCAT
Sibynomorphus_mikanii_LWS        TGAGAAGGAAGTATCAAGGATGGTAGTGGTCATGATCATTGCCTATATTATCTGCTGGGGACCATATGCATCTTTTGCCTGTTTTGCTGCTGCCAATCCAGGCTACGCCTTTCACCCCCT
Sibynomorphus_neuwiedii_LWS      TGAGAAGGAAGTATCAAGGATGGTAGTGGTCATGATCATTGCCTATATTATCTGCTGGGGACCATATGCATCTTTTGCCTGTTTTGCTGCTGCCAATCCCGGCTACGCCTTTCACCCCCT
Spilotes_pullatus_LWS            TGAGAAGGAAGTATCAAGGATGGTAGTGGTCATGATCATTGCCTATATTGTCTGCTGGGGACCATATACATTTTTTGCCTGTTTTGCTGCTGCCAATCCAGGCTATGCCTTTCACCCCCT
Taeniophallus_persimilis_LWS     TGAGAAGGAAGTATCAAGGATGGTAGTGGTCATGATCATTGCCTATATTGTCTGCTGGGGACCATATACATTTTTTGCCTGTTTTGCTGTTGCCAATCCAGGCTATGCCTTTCACCCCCT
Thamnodynastes_strigatus_LWS     TGAGAAGGAAGTATCAAGGATGGTAGTGGTCATGATCATTGCTTTTATTGTCTGCTGGGGACCATATACATTTTTTGCCTGTTTTGCTGCTGCCAATCCAGGCTATGCCTTTCACCCCCT
Tomodon_dorsatus_LWS             TGAGAAGGAAGTATCAAGGATGGTAGTGGTCATGATCATTGCTTTTATTGTTTGCTGGGGACCATATACATTTTTTGCCTGTTTTGCTGCTGCCAATCCAGGCTATGCCTTTCACCCCCT
Xenopeltis_unicolor_LWS          TGAGAAGGAAGTATCAAGGATGGTAGTGGTCATGATCCTTGCCTATATTGTCTGCTGGGGACCATATACAACTTTTGCCTGTTTTGCTGCTGCCAATCCAGGCTATGCCTTTCATCCCAT
Atractus_reticulatus_SWS1        CGAGCGAGAGGTCTCCCGCATGGTGGTGGTGATGGTGGCATCCTTCTGCATCTGCTACGTTCCGTACGCCGCCCTGGCCATGTACATGGTGAATAATCCTCATCACGGCTTCGACCTACG
Chironius_bicarinatus_SWS1       CGAGCGAGAGGTCTCCCGCATGGTGGTGGTGATGGTGGGATCCTTCGGCATGTGCTACATTCCGTACGCCGCCCTGGCCATGTACATGGTGAACAACCCTCAGCACAACCTCGACCTGCG
Dipsas_petersi_SWS1              CGAGCGAGAGGTCTCCCGCATGGTGGTGGTGATGGTGGCATCCTTCTGCATCTGCTACGTTCCGTATGCCGCCCTGGCCATGTACATGGTGAATAATCCTCATCACGGCTTCGACCTGCG
Echinantera_cephalostriata_SWS1  CGAGCGAGAGGTCTCCCGCATGGTGGTGGTGATGGTGGGATCCTTCTGCGTCTGCTATGTTCCATACGCCGCCCTGGCCATGTACATGGTGAATAATCCTCAGCACGGCTTCGACCTGCG
Echinantera_undulata_SWS1        CGAGCGAGAGGTCTCCCGCATGGTGGTGGTGATGGTGGGATCCTTCTGCGTCTGCTATGTTCCATACGCCGCCCTGGCCATGTACATGGTGAATAATCCTCAGCACGGCTTCGACCTGCG
Erythrolamprus_aesculapii_SWS1   CGAGAAAGAGGTCTCCCGCATGGTGGTGGTGATGGTGGGGTCCTTCTGCGTCTGCTATGTTCCATACGCTGCCCTGGCCATGTACATGGTGAATAATCCTCAGCACGGCTTCGACCTGCG
Erythrolamprus_miliaris_SWS1     CGAGAGAGAGGTCTCCCGCATGGTGGTGGTGATGGTGGGGTCRTTCTGCATCTGCTATGTTCCCTAYGCCGCCCTGGCCATGTACATGGTGAATAATCCTCAGCACGGCTTCGACCTGCG
Erythrolamprus_poecilogyrus_SWS1 CGAGAGAGAGGTCTCCCGCATGGTGGTGGTGATGGTGGGGTCCTTCTGCATCTGCTATGTTCCATACGCCGCCCTGGCCATGTACATGGTGAATAATCCTCAGCACGGCTTCGACCTGCG
Helicops_modestus_SWS1           CGAGCGAGAGGTCTCCCGCATGGTGGTGGTGATGGTGGGCTCCTTCTGCGTCTGCTATGTTCCATACGCCGCCCTGGCCATGTACATGGTGAATAATCCTCAGCACGGCWTCGACTTGCG
Oxyrhopus_guibei_SWS1            CGAGCGAGAGGTCTCCCGCATGGTGGTGGTGATGGTGGGATCCTTCTGCGTCTGCTATGTTCCATACGCCGCCATGGCCATGTACATGGTGAATAATCCTCAGCACGGCTTCGACCTGCG
Philodryas_patagoniensis_SWS1    CGAGCGAGAGGTCTCCCGCATGGTGGTGGTGATGGTGGGATCCTTCTGCATCTGCTATGTTCCATACGCCGCCCTGGCCATGTACATGGTGAATAATCCTCAGCACGGYTTCGACCTGCG
Python_regius_SWS1               TGAGCGAGAGGTCTCCCGCATGGTGGTGGTGATGGTGGGGTCCTTTTGCCTCTGCTATGTTCCATACGCCACCCTGGCCATGTACATGGTGAATCACCCTAAGCACGGCCTTGACCTCCG
Sibynomorphus_mikanii_SWS1       CGAGCGAGAGGTCTCCCGCATGGTGGTGGTGATGGTGGCATCCTTCTGCATCTGCTACGTTCCGTATGCCGCCCTGGCCATGTACATGGTGAATAATCCTCATCACGGCTTCGATCTGCG
Sibynomorphus_neuwiedii_SWS1     CGAGCGAGAGGTCTCCCGCATGGTGGTGGTGATGGTGGCATCCTTCTGCATCTGCTACGTTCCGTATGCCGCCCTGGCCATGTACATGGTGAATAATCCTCATCACGGCTTYGACCTGCG
Spilotes_pullatus_SWS1           CGAGCGAGAGGTCTCCCGCATGGTGGTGGTGATGGTGGGATCCTTCTGCATCTGCTACATTCCGTACGCCGCCCTAGCCATGTATATGGTGAACAACCCTCAGCACGGCCTCGACCTGCG
Taeniophallus_persimilis_SWS1    CGAGCGAGAGGTCTCCCGCATGGTGGTGGTGATGGTGGGATCCTTCTGCGTCTGCTATGTTCCATACGCTGCCCTGGCCATGTACATGGTGAATAATCCTCAGCACGGCTTCGACCTGCG
Thamnodynastes_strigatus_SWS1    CGAGCGAGAGGTCTCCCGCATGGTGGTGGTGATGGTGGGATCCTTCTGCGTCTGCTATGTTCCGTACGCCGCCCTGGCCATGTACATGGTGAATAATCCTCAGCACGGCTTCGACCTGCG
Tomodon_dorsatus_SWS1            CGAGCGAGAGGTCTCCCGCATGGTGGTGGTGATGGTGGGATCCTTCGGCGTCTGCTATGTTCCATACGCCGCCCTGGCCATGTACATGGTGAATAATCCTCAGCACGGCTTCGACCTGCG
Xenopeltis_unicolor_SWS1         TGAGCGAGAGGTCTCCCGCATGGTGGTGGTGATGGTGGGGTCCTTTTGCCTCTGCTACGTTCCATATGCCGCCCTGGCCATGTACATGGTGAATCATCCTAAGCACGGCCTCGACCTGCG
```

```
                                1011      1020      1030      1040      1050      1050      1060      1070      1080      1090      1100      1110      1120
                                |         |         |         |         |         |         |         |         |         |         |         |         |
Bos_taurus_Rh1                  CTTCATGACCATCCCGGCTTTCTTTGCCAAGACTTCTGCCGTCTACAACCCCGTCATCTACATCATGATGAACAAGCAGTTCCGGAACTGCATGGTCACCACTCTCTGCTGTGGCAAGAA
Atractus_reticulatus_Rh1        CTTTATGACCATCCCGGCCTTCTTTGCCAAGAGCTCTGCAATCTACAACCCAGTGATTTATATCGTAATGAACAAACAGTTCCGCAATTGTATGATCACCACACTTTGCTGTGGAAAGAA
Chironius_bicarinatus_Rh1       CTTTATGACCATCCCATCCTTCTTTGCCAAGAGCTCTGCAATCTACAACCCAGTGATTTATATCGTAATGAACAAACAGTTCCGCAACTGTATGCTCACCACACTTTGCTGTGGAAAGAA
Dipsas_petersi_Rh1              CTTTATGACCATCCCAGCCTTCTTTGCCAAGAGCTCTTCAATCTACAACCCAGTGATTTATATCGTAATGAACAAACAGTTCCGCAATTGTATGATCACCACACTTTGCTGTGGAAAGAA
Echinantera_cephalostriata_Rh1  CTTTATGACCATCCCGTCCTTCTTTGCCAAGAGCTCTGCAATCTACAACCCAGTGATTTATATCGTAATGAACAAACAGTTCCGCAATTGTATGCTCACCACACTTTGCTGTGGAAAGAA
Echinantera_undulata_Rh1        CTTTATGACCATCCCGTCCTTCTTTGCCAAGAGCTCTGCAATCTACAACCCAGTGATTTATATCGTAATGAACAAACAGTTCCGCAATTGTATGCTCACCACACTTTGCTGTGGAAAGAA
Erythrolamprus_aesculapii_Rh1   CTTTATGACCATCCCGTCCTTCTTTGCCAAGAGCTCTGCAATCTACAACCCAGTGATTTATATCGTAATGAACAAACAGTTCCGCAATTGTATGCTCACCACACTTTGTTGTGGAAAGAA
Erythrolamprus_miliaris_Rh1     CTTTATGACCATCCCGTCCTTCTTTGCCAAGAGCTCTGCAATCTACAACCCAGTGATTTATATCGTAATGAACAAACAGTTCCGCAATTGTATGCTCACCACACTTTGTTGTGGAAAGAA
Erythrolamprus_poecilogyrus_Rh1 CTTTATGACCATCCCGTCCTTCTTTGCCAAGAGCTCTGCAATCTACAACCCAGTGATTTATATCGTAATGAACAAACAGTTCCGCAATTGTATGCTCACCACACTTTGTTGTGGAAAGAA
Helicops_modestus_Rh1           CTTTATGACCATCCCGTCCTTCTTTGCCAAGAGCTCTGCAATCTACAACCCAGTGATTTATATCGTAATGAACAAACAGTTCCGCAATTGCATGCTCACCACACTTTGCTGTGGAAAGAA
Oxyrhopus_guibei_Rh1            CTTTATGACCGCCCCAGCCTTCTTTGCCAAGAGCTCTTCAATCTACAACCCAGTGATTTATATCGTAATGAACAAACAGTTCCGCAATTGTATGATCACCACTCTTTGCTGTGGAAAGAA
Philodryas_patagoniensis_Rh1    CTTTATGACCATCCCGTCCTTCTTTGCCAAGAGCTCTGCAATCTACAACCCAGTGATTTATATCGTAATGAACAGACAGTTCCGCAATTGTATGCTCACCACACTTTGCTGTGGAAAGAA
Python_regius_Rh1               CTTTATGACTCTCCCAGCCTTCTTTGCCAAGAGCTCTGCGATCTACAATCCAGTGATTTACATCGTATTGAACAAACAGTTCCGCAACTGTATGATCACCACACTTTGCTGTGGAAAGAA
Sibynomorphus_mikanii_Rh1       CTTTATGACCATCCCAGCCTTCTTTGCCAAGAGCTCTTCAATCTACAACCCAGTGATTTATATCGTAATGAACAAACAGTTCCGCAATTGTATGATTACCACACTTTGCTGTGGAAAGAA
Sibynomorphus_neuwiedi_Rh1      CTTTATGACCATCCCAGCCTTCTTTGCCAAGAGCTCTTCAATCTACAACCCAGTGATTTATATCGTAATGAACAAACAGTTCCGCAATTGTATGATCACCACACTTTGCTGTGGAAAGAA
Spilotes_pullatus_Rh1           CTTTATGACCATCCCATCCTTCTTTGCCAAGAGCTCTGCAATCTACAACCCAGTGATTTATATCGTAATGAACAAACAGTTCCGCGACTGTATGCTCACCACACTTTGCTGTGGAAAGAA
Taeniophallus_persimilis_Rh1    CTTTATGACCATCCCGTCCTTCTTTGCCAAGAGCTCTGCAATCTACAACCCAGTGATTTATATCGTAATGAACAAACAGTTCCGCAATTGTATGCTCACCACACTTTGCTGTGGAAAGAA
Thamnodynastes_strigatus_Rh1    CTTTATGACCATCCCGTCCTTCTTTGCCAAGAGCTCTGCAATCTACAACCCAGTGATTTATATCGTAATGAACAAACAGTTCCGCAATTGTATGCTCACCACACTTTGCTGTGGAAAGAA
Tomodon_dorsatus_Rh1            CTTTATGACCATCCCATCCTTCTTTGCCAAGAGCTCTGCAATCTACAACCCAGTGATTTATATCGTAATGAACAAACAGTTCCGCAATTGTATGCTCACCACACTTTGCTGTGGAAAGAA
Xenopeltis_unicolor_Rh1         CTTTATGACCCTCCCAGCCTTCTTTGCCAAGAGCTCTGCAATCTACAATCCGGTGATTTACATCGTATTGAACAAACAGTTCCGCAACTGTATGATCACCACGCTTTGCTGTGGAAAGAA
Atractus_reticulatus_LWS        GACAGCTTCCCTGCCTGCCTTTTTTGCAAAAAGCGCCACCATTTACAACCCAATTATCTATGTCTTCATGAACAGACAGTTCCGCAATTGCATAATGC--------------------AG
Chironius_bicarinatus_LWS       GACGGCTTCCATGCCTGCCTTCTTTGCAAAAAGCGCCACCATTTACAACCCAATTATCTATGTCTTCATGAACAGACAGTTCCGTAATTGCATAATGC--------------------AG
Dipsas_petersi_LWS              GACAGCTTCCCTGCCTGCCTTTTTTGCAAAAAGCGCCACCATTTACAACCCAATTATCTACGTCTTCATGAACAGACAGTTCCGGAATTGCATAATGC--------------------AG
Echinantera_cephalostriata_LWS  GACAGCTTCCATGCCTGCCTTCTTTGCAAAAAGCGCCACCATTTACAACCCAATTATCTATGTCTTCATGAACAGACA------------------------------------------
Echinantera_undulata_LWS        GACAGCTTCCATGCCTGCCTTCTTTGCAAAAAGCGCCACCATTTACAACCCAATTATCTATGTCTTCATGAACAGACAGTTCCGTAATTGCATAATGC--------------------AG
Erythrolamprus_aesculapii_LWS   GACAGCTTCCATGCCTGCCTTCTTTGCAAAAAGCGCCACCATTTACAACCCAATTATCTATGTCTTCATGAACAGACAGTTCCGTAATTGC-----------------------------
Erythrolamprus_miliaris_LWS     GACAGCTTCCATGCCTGCCTTCTTTGCAAAAAGCGCCACCATTTACAACCCAATTATCTATGTCTTCATGAACAGACAGTTCCGTAATTGCATAATGC--------------------AG
Erythrolamprus_poecilogyrus_LWS GACAGCTTCCATGCCTGCCTTCTTTGCAAAAAGCGCCACCATTTACAACCCAATTATCTATGTCTTCATGAACAGACAGTTCCGTAATTGCATAATGC--------------------AG
Helicops_modestus_LWS           GGCAGCTTCCTTGCCTGCCTTCTTTGCAAAAAGCGCCACCATTTACAACCCAATTATCTATGTCTTCATGAACAGACAGTTCCGTAATTGCATAATGC--------------------AG
Oxyrhopus_guibei_LWS            GACAGCTTCCATGCCTGCCTTCTTTGCAAAAAGCGCCACCATTTACAACCCGATTATCTATGTCTTCATGAACAGACAGTTCCGTAATTGCATAATGC--------------------AG
Philodryas_patagoniensis_LWS    GACAGCTTCCATGCCTGCCTTCTTTGCAAAAAGCGCTACCATTTACAACCCAATTATCTATGTCTTCATGAACAGACAGTTCCGTAATTGCATAATGC--------------------AG
Python_regius_LWS               GACAGCTTCTTTGCCTGCCTTCTTTGCAAAAAGCGCCACCATTTACAACCCAATTATCTATGTCTTCATGAACAGACAGTTCCGTAATTGCATAATGC--------------------AG
Sibynomorphus_mikanii_LWS       GACAGCTTCCCTGCCTGCCTTTTTTGCAAAGAGCGCCACCATTTACAACCCAATTATCTACGTCTTCATGAACAGTCAGTT---------------------------------------
Sibynomorphus_neuwiedii_LWS     GACAGCTTCCCTGCCTGCCTTTTTTGCAAAAAGCGCCACCATTTACAACCCAATTATCTACGTCTTCATGAACAGACAGTTCCGGAATTGCATAATGC--------------------AG
Spilotes_pullatus_LWS           GACGGCTTCCATACCTGCCTTCTTTGCAAAAAGCGCCACCATTTACAACCCAATTATCTATGTCTTCATGAACAGACAGTTCCGTAATTGC-----------------------------
Taeniophallus_persimilis_LWS    GACAGCTTCCATGCCTGCCTTCTTTGCAAAAAGCGCCACCATTTACAACCCAATTATCTATGTCTTCATGAACAGACAGTTCCGTAATTGCATAATGCA---------------------
Thamnodynastes_strigatus_LWS    GACAGCTTCCATGCCTGCCTTCTTTGCAAAAAGCGCCACCATTTACAACCCAATTATCTATGTCTTCATGAACAGACAGTTCCGTAATTGCATCATGC--------------------AG
Tomodon_dorsatus_LWS            GACAGCTTCCATGCCTGCCTTCTTTGCAAAAAGCGCAACCATTTACAACCCAATTATCTATGTCTTCATGAACAGACAGTTCCGTAATTGCATCATGC--------------------AG
Xenopeltis_unicolor_LWS         GACAGCTTCTTTGCCTGCCTTCTTTGCAAAAAGCGCCACCATTTATAACCCAATTATCTATGTCTTCATGAACAGACAGTTCCGTAATTGCATAATGC--------------------AG
Atractus_reticulatus_SWS1       CTTGGTCACCATCCCTGCCTTCTTCTCCAAGAGCTCCTGCGTCTACAACCCCATCATTTACTGCTTCATGAACAAGCAGTTCCGTGCCTGCATCATGGAAACGGTGTGYGGC------AA
Chironius_bicarinatus_SWS1      CTTGGTCACCATCCCTGCCTTCTTCTCCAAGAGCTCCTGCGTCTACAACCCCATCATCTACTGCTTCATGAACAAGCAGTTCCGTGCCTGCATCATGGAAACGGTGTGTGGC------AA
Dipsas_petersi_SWS1             CTTCGTCACCATCCCTGCCTTCTTCTCCAAGAGCTCCTGCGTCTATAACCCCATCATCTACTGCTTCATGAACAAGCAGTTCCGTGCCTGCATCATGGAAACGGTGTGCGGC------AA
Echinantera_cephalostriata_SWS1 CTTGGTCACCATCCCTGCCTTCTTCTCYAAAAGCTCCTGCGTCTACAACCCCATCATCTACTGCTTCATGAACAAGCAGTTCCGGGCCTGCATCATGGAAACGGTGTGCGGC------AA
Echinantera_undulata_SWS1       CTTGGTCACCATCCCTGCCTTCTTCTCCAAAAGCTCCTGCGTCTACAACCCCATCATCTACTGCTTCATGAACAAGCAGTTCCGGGCCTGCATCATGGAAACGGTGTGCGGC------AA
Erythrolamprus_aesculapii_SWS1  CTTGGTCACCATCCCTGCCTTCTTCTCCAAAAGCTCCTGCGTCTACAACCCCATCATCTACTGCTTCATGAACAAGCAGTTCCGTGCCTGCATCATGGAAACGGTGTGCGGC------AA
Erythrolamprus_miliaris_SWS1    CTTGGTCACCATCCCTGCCTTCTTCTCCAAAAGCTCCTGCGTCTACAACCCCATCATCTACTGCTTCATGAACAAGCAGTTCCGTGCCTGCATCATGGAAACGGTGTGCGGC------AA
Erythrolamprus_poecilogyrus_SWS1 CTTGGTCACCATCCCTGCCTTCTTCTCCAAAAGCTCCTGCGTCTACAACCCCATCATCTACTGCTTCATGAACAAGCAGTTCCGTGCCTGCATCATGGAAAC------------------
Helicops_modestus_SWS1          CTTGGTCACCATCCCKGCCTTYTTYTCCAAAAGCTCCTGCGTCTACAACCCCRTCATCTACTGCTTCATGAACAAGCAGTTCCGTGCCTGCATCATGGAAACGGTGTGCGGC------AA
Oxyrhopus_guibei_SWS1           CTTGGTCACCATCCCTGCCTTCTTCTCCAAAAGCTCCTGTGTCTACAACCCCATCATCTACTGTTTCATGAACAAGCAATTCCGTGCCTGCATCATGGAAATGGTGTGCGGC------AA
Philodryas_patagoniensis_SWS1   CTTGGTCACCATCCCTGCCTTCTTCTCCAAAAGCTCCTGCGTCTACAACCCCATCATCTACTGCTTCATGAACAAGCAGTTCCGTGCCTGCATCA-------------------------
Python_regius_SWS1              CTTGGTCACCATCCCTGCCTTCTTCTCCAAGAGTTCCTGCGTCTACAATCCCATCATCTACTGCTTCATGAATAAGCAGTTCCGTGCCTGCATCATGCAAACCGTGTGTGGC------AA
Sibynomorphus_mikanii_SWS1      CTTGGTCACCATCCCTGCCTTCTTCTCCAAGAGCTCCTGCGTCTACAACCCCATCATCTACTGCTTCATGAACAAGCAGTTCCGTGCCTGCATCATGGAAACGGTGTGCGGC------AA
Sibynomorphus_neuwiedii_SWS1    CTTGGTCACCATCCCTGCCTTCTTCTCCAAGAGCTCCTGCGTCTACAACCCCATCATCTACTGCTTCATGAACAAGCAGTTCCGTGCCTGCATCATGGAAACGGTGTGCGGC------AA
Spilotes_pullatus_SWS1          CTTGGTCACCATCCCTGCCTTCTTCTCCAAGAGCTCCTGCGTCTATAACCCCATCATCTACTGCTTCATGAACAAGCAGTTCCGTGCCTGCATCATGGAAACGGTGTGTGGC------AA
Taeniophallus_persimilis_SWS1   CTTGGTCACCATCCCTGCCTTCTTCTCCAAAAGCTCCTGCGTCTACAACCCCATCATCTACTGCTTCATGAACAAGCAGTTCCGTGCCTGCATCATGGAAACGGTGTGCGGC------AA
Thamnodynastes_strigatus_SWS1   CTTGGTCACCATCCCGGCCTTCTTCTCCAAAAGCTCCTGCGTCTACAACCCCATCATCTACTGCTTCATGAACAAGCAGTTCCGTGCCTGCATCATGGAAACGGTGTGCGGC------AA
Tomodon_dorsatus_SWS1           CTTGGTCACCATCCCGGCCTTCTTCTCCAAAAGCTCCTGCGTCTACAACCCCATCATCTACTGCTTCATGAACAAGCAGTTCCGTGCCTGCATCATGGAAACGGTGTGCGGC------AA
Xenopeltis_unicolor_SWS1        TTTGGTCACCATCCCCGCCTTCTTCTCCAAGAGCTCCTGCGTCTACAACCCCATCATCTACTGCTTCATGAATAAGCAGTTCCGTTCCTGCATCATGGAAACCGTGTGCGGC------AA
```

```
                                 1121      1130      1140      1150      1160      1170      1180      1190      1200      1210      1220      1230      1240
                                 |         |         |         |         |         |         |         |         |         |         |         |         |
Bos_taurus_Rh1                   CCCGCTGGGT--------GACGACGAGGCCTCCA---CCACCGTCTCCAAGACAGAGACCAGCCAAGTGGCGCCTGCCTAA---------------------------------------
Atractus_reticulatus_Rh1         TCCTTTGGCA--------GA----------------------------------------------------------------------------------------------------
Chironius_bicarinatus_Rh1        TCCTTTGGTG--------GAGGATGACACTTCCG------TTGGCACCAAGACAGAGACATCTACAGTCTCCACAAGT------------------------------------------
Dipsas_petersi_Rh1               TCCTTTGGCA--------GAGGATGACACTTCTG------CTGGCACCAAGACAGAGACATCTACAGTCTCCACAAGTAAGGTTTCCCC-------------------------------
Echinantera_cephalostriata_Rh1   TCCTTTGGCA--------GAGGATGACACTTCTG------CTGGCACCAAGACAGAGACATCTACAGTCTCCACAAGTAAGGTTTTC---------------------------------
Echinantera_undulata_Rh1         TCCTTTGGCA--------GAGGATGACACTTCTG------CTGGCACCAAGACAGAGACATCTACAGTCTCCACAAGTAAGGTTTCCCC-------------------------------
Erythrolamprus_aesculapii_Rh1    TCCTTTGGCA--------GAGGACGACACTTCTG------CTGGCACCAAGACAGAGACATCTACAGTCTCCACAAGTAAGGTTT-----------------------------------
Erythrolamprus_miliaris_Rh1      TCCTTTGGCA--------GAGGACGACACTTCTG------CTGGCACCAAGACAGAGACATCTACAGTCTCCACAAGTAAGGTTT-----------------------------------
Erythrolamprus_poecilogyrus_Rh1  TCCTTTGG----------------------------------------------------------------------------------------------------------------
Helicops_modestus_Rh1            TCCTTTGGCA--------GAGGATGACACTTCTG------CTGGAACCAAGACAGAGACATCTACAGTCTCCACAAGTAAGGTTTTC---------------------------------
Oxyrhopus_guibei_Rh1             TCCTTTGGGA--------GAAGAAGACACTTCTG------CTGGCACCAAGACAGAGACATCTACAGTCTCCACAAGTAAGGTTTCCCC-------------------------------
Philodryas_patagoniensis_Rh1     TCCTTTGGCA--------GAGGATGACACTTCTG------CTGGCACCAAGACAGAGACTTCTACAGTCTCCACAAGTAAGGTTTCCC--------------------------------
Python_regius_Rh1                TCCTTTGGCA--------GAGGATGACACTTCTG------CTGGCACCAAGACAGAGACATCTACAGTCTCC------------------------------------------------
Sibynomorphus_mikanii_Rh1        TCCTTTGGCA--------GAGGAGGACACTTCTG------CTGGCACCAAGACAGAGACATCTACAGTCTCCACAAG-------------------------------------------
Sibynomorphus_neuwiedi_Rh1       TCCTTTGGCA--------GAGGAGGACACTTCTG------CTGGCACCAAGACAGAGACATCTACAGTCTCCACAAGTA-----------------------------------------
Spilotes_pullatus_Rh1            TCCTTTGGCA--------GAGGATGACACTTCTG------CTGGCACCAAGACAGAGACATCTACAGTCTCCACA---------------------------------------------
Taeniophallus_persimilis_Rh1     TCCTTTGGCA--------GAGGATGACACTTCTG------CTGGCACCAAGACAGAGACATCTACAGTCTCCACAAGTA-----------------------------------------
Thamnodynastes_strigatus_Rh1     TCCTTTGGCA--------GAGGATGACACTTCTG------CTGGCACCAAGACAGAGACATCTACAGTCTCCACAAGTA-----------------------------------------
Tomodon_dorsatus_Rh1             TCCTTTGGCA--------GAGGATGACACTTCTG------CTGGCACCAAGACAGA----------------------------------------------------------------
Xenopeltis_unicolor_Rh1          CCCTTTGGCA--------GAGGATGACACTTCTG------CTGGCACCAAGACAGAGACATCTACAGTCTCCACAAGTCAGGTTTCCCCTGCCTAG------------------------
Atractus_reticulatus_LWS         CTCTTTGGCC--------AAGAAAGT----------------------------------------------------------------------------------------------
Chironius_bicarinatus_LWS        CTCTT-------------------------------------------------------------------------------------------------------------------
Dipsas_petersi_LWS               CTCTTTGGCA--------AGAAAGTGGA--------------------------------------------------------------------------------------------
Echinantera_cephalostriata_LWS   ------------------------------------------------------------------------------------------------------------------------
Echinantera_undulata_LWS         CTCTTGGGCA--------A-----------------------------------------------------------------------------------------------------
Erythrolamprus_aesculapii_LWS    ------------------------------------------------------------------------------------------------------------------------
Erythrolamprus_miliaris_LWS      CTCTGTGGGA--------A-----------------------------------------------------------------------------------------------------
Erythrolamprus_poecilogyrus_LWS  CTCTTTGG----------------------------------------------------------------------------------------------------------------
Helicops_modestus_LWS            CTCTTTG-----------------------------------------------------------------------------------------------------------------
Oxyrhopus_guibei_LWS             CTCTTTGGCA--------AGAAAGTGGA--------------------------------------------------------------------------------------------
Philodryas_patagoniensis_LWS     CTCTTTGGCA--------A-----------------------------------------------------------------------------------------------------
Python_regius_LWS                CTCTTTGGCAAGAAAGTGGATGATGGTTCTGAAGTTTCCTCTACTTCCCGCACTGAAGTTTCATCTGTCTCT------------------------------------------------
Sibynomorphus_mikanii_LWS        ------------------------------------------------------------------------------------------------------------------------
Sibynomorphus_neuwiedii_LWS      CTCTTTG-----------------------------------------------------------------------------------------------------------------
Spilotes_pullatus_LWS            ------------------------------------------------------------------------------------------------------------------------
Taeniophallus_persimilis_LWS     ------------------------------------------------------------------------------------------------------------------------
Thamnodynastes_strigatus_LWS     CTCTTTG-----------------------------------------------------------------------------------------------------------------
Tomodon_dorsatus_LWS             CTCTTTGCAA--------AAAAAGTGG---------------------------------------------------------------------------------------------
Xenopeltis_unicolor_LWS          CTCTTTGGCAAGAAAGTGGATGATGGTTCTGAAGTTTCCTCTACTTCCCGCACTGAAGTCTCATCTGTCTCTAACTCTTCTGTATCGCCAGCATAA------------------------
Atractus_reticulatus_SWS1        ACCTATAACG--------GACGATTCTGATGTGA------GCTCTCAGAAGACGGARGTTTCCTCAGCGTCCTCCTGTCAAGTCAGCCA-------------------------------
Chironius_bicarinatus_SWS1       ACCTATGACG--------GACGACTCTGACGCGG------TCTCTCAGAAGACGGAGGTTTCCTCCGCGTCCTCCTGTAAAGTCAGCCCA------------------------------
Dipsas_petersi_SWS1              ACCTATGACG--------GACGAGTCTGACCTGA------GCTCTCAGAAGACGGAGGTTTCCTCAGCGTCCTCCTGTCAAGTCAGCC--------------------------------
Echinantera_cephalostriata_SWS1  ACCTATGACG--------GACGAGTCTGATGTGA------CTC-----------------------------------------------------------------------------
Echinantera_undulata_SWS1        ACCTATGACG--------GACGACTCTGATGTGA------CTTTCTCAAAAAAA------------------------------------------------------------------
Erythrolamprus_aesculapii_SWS1   ACCCATGACG--------GACGAGTCCGATGCGA------CT------------------------------------------------------------------------------
Erythrolamprus_miliaris_SWS1     ACCCATGACA--------GACGACTCCGACGTGA------C-------------------------------------------------------------------------------
Erythrolamprus_poecilogyrus_SWS1 ------------------------------------------------------------------------------------------------------------------------
Helicops_modestus_SWS1           ACCTATGACG--------GACGACTCTGATGTGA------GTTCTCAGAGGACGGAGGTTTCCTCGGCGTCCTCCTGTCAAGTCAGCCA-------------------------------
Oxyrhopus_guibei_SWS1            AC----------------------------------------------------------------------------------------------------------------------
Philodryas_patagoniensis_SWS1    ------------------------------------------------------------------------------------------------------------------------
Python_regius_SWS1               GCCTCTGACG--------GAGGAGTCTGATGCGGGCAGCTCCGCTCAGAAAACGGAGGTTTCGTCGGTGTCCTCCAGCCAAGTCAGCCCGAGCTCCGGAGCCGGC---------------
Sibynomorphus_mikanii_SWS1       ACCTATGACG--------GACGAGTCTGACCTGA------G-------------------------------------------------------------------------------
Sibynomorphus_neuwiedii_SWS1     ACCTATGATG--------GACGAGTCTGACGTGA------GCTCTCAGAAGACGGAGGTTTCCTCAGTGTC-------------------------------------------------
Spilotes_pullatus_SWS1           ACCTATGACG--------GACGACTCTGATGTGA------CTTTC---------------------------------------------------------------------------
Taeniophallus_persimilis_SWS1    ACCTATGACG--------GACGAATCTGATGTGA------CT------------------------------------------------------------------------------
Thamnodynastes_strigatus_SWS1    ACCTATGACG--------GACGACTCTGATGTGC--------------------------------------------------------------------------------------
Tomodon_dorsatus_SWS1            ACCTATGACG--------GACGACTCTGATGTGA------CTTCTCAGAAGACGGAGGTTTCCTCAGTGTCCTCCTGTCAAGTCAGCCCA------------------------------
Xenopeltis_unicolor_SWS1         ACCTCTGACG--------GACGAGTCCGACGCGGGCAGCTCCGTTCAGAAAACGGAAGTTTCGTCGGTGTCCTCCTGTCAAGTCAGCCCGAGCTCAGGAGCCGGCACCACGGGTTCTCCA
```

Additional File 2: Figure S3. 2. Amino acid alignment of the visual pigments RH1, LWS and SWS1 expressed in snakes retinas and the bovine rhodopsin RH1.

```
                                    1        10        20        30        40        50        60        70        80        90        100       110       120
Bos_taurus_Rh1                      MNGTEGPNFYVPFSNKTGVVRSPFEAPQYYLAEPWQFSMLAAYMFLLIMLGFPINFLTLYVTVQHKKLRTPLNYILLNLAVADLFMVFGGFTTTLYTSLHGYFVFGPTGCNLEGFFATLG
Atractus_reticulatus_Rh1            -----------PMSNKTGIVRSPYEYPQYYLADPWKYSALAAYMFLLILLGFPINFLTLYVTIQHKKLRTPLNYILLNLAIANLFMVLAGFTTTMYTSMNGYFVFGIVGCNIEGFFATLG
Chironius_bicarinatus_Rh1           -------------------VRSPFEYPQYYLADPWKYSALAAYMFLLILLGFPINFLTLYVTIQHKKLRTPLNYILLNLAVANLFMVLVGFTTTMYTSMNGYFIFGTVGCNVEGFFATLG
Dipsas_petersi_Rh1                  ---------------KTGVVRSPYEYPQYYLADPWKYSALAAYMFLLILLGFPINFLTLFVTIQHKKLRTALNYILLNLAVADLFMVLAGFTTTMYTSMNGYFVFGVVGCNIEGFFATLG
Echinantera_cephalostriata_Rh1      --------------NKTGIVRSPYEYPQYYLADPWKYSALAAYMFLLILLGFPINFLTLYVTIQHKKLRTPLNYILLNLAVANLFMVLVGFTTTMYTSMNGYFIFGTIGCNVEGFFATLG
Echinantera_undulata_Rh1            ----------------------PFEYPQYYLADPWKYSALAAYMFLLILLGFPINFLTLYVTIQHKKLRTPLNYILLNLAVANLFMVLVGFTTTMYTSMNGYFIFGTIGCNVEGFFATLG
Erythrolamprus_aesculapii_Rh1       ----------VPMSNKTGIVRSPYEYPQYYLADPWKYSALAAYMFLLILLGFPINFLTLYVTIQHKKLRTPLNYILLNLAVANLFMVLVGFTTTMYTSMNGYFIFGTIGCNVEGFFATLG
Erythrolamprus_miliaris_Rh1         ----------VPMSNKTGIVRSPYEYPQYYLADPWKYSALAAYMFLLILLGFPINFLTLYVTIQHKKLRTPLNYILLNLAVANLFMVLVGFTTTMYTSMNGYFIFGTIGCNVEGFFATLG
Erythrolamprus_poecilogyrus_Rh1     -----------------------------------------------------IHFLTLYVTIQHKKLRTPLNYILLNLAVANLFMVLVGFTTTMYTSMNGYFIFGTIGCNVEGFFATLG
Helicops_modestus_Rh1               --------------NKTGVVRSPYEYPQYYLADPWKYSALAAYMFLLILLGFPINFLTLYVTIQHKKLRTPLNYILLNLAVANLFMVLVGFTTTMYTSMNGYFIFGTIGCNVEGFFATLG
Oxyrhopus_guibei_Rh1                -------------------VRSPFEYPQYYLADPWKYSALAAYMFLLILLGFPINFLTLYVTIQHKKLRTALNYILLNLAVADLFMVLAGFTTTMYTSMNGYFVFGTVGCNIEGFFATLG
Philodryas_patagoniensis_Rh1        --------------------------------------------FLLILLGFPINFLTLFVTIQHKKLRTPLNYILLNLAVANLFMVLVGFTTTMYTSMNGYFVFGTIGCNVEGFFATLG
Python_regius_Rh1                   -------NFYVPMSNKTGIVRSPFEYPQYYLAEPWKYSALGAYMFLLILLGFPINFLTLYVTIQHKKLRTPLNYILLNLAIANLFMVLVGFTTTMYTSMNGYFVFGTVGCNVEGFFATLG
Sibynomorphus_mikanii_Rh1           ---------------------------------PWKYSALAAYMFLLILLGFPINFLTLFVTIQHKKLRTALNYILLNLAVADLFMVLAGFTTTMYTSMNGYFVFGVVGCNIEGFFATLG
Sibynomorphus_neuwiedi_Rh1          ---------------------------------PWKYSALAAYMFLLILLGFPINFLTLFVTIQHKKLRTALNYILLNLAVADLFMVLAGFTTTMYTSMNGYFVFGVVGCNIEGFFATLG
Spilotes_pullatus_Rh1               --------------------------LSYYLADPWKYSALAAYMFLLILLGFPINFLTLYVTIQHKKLRTPLNYILLNLAVANLFMVLVGFTTTMYTSMNGYFIFGTVGCNVEGFFATLG
Taeniophallus_persimilis_Rh1        --------------NKTGIVRSPYEYPQYYLADPWKYSALAAYMFLLILLGFPINFLTLYVTIQHKKLRTPLNYILLNLAVANLFMVLVGFTTTMYTSMNGYFIFGTIGCNVEGFFATLG
Thamnodynastes_strigatus_Rh1        --------------------------PQYYLADPWKYSALAAYMFLLILLGFPINFLTLYVTIQHKKLRTPLNYILLNLAVANLFMVLVGFTTTMYTSMNGYFIFGTIGCNVEGFFATLG
Tomodon_dorsatus_Rh1                -------------------VRSPFEYPQYYLADPWKYSALAAYMFLLILLGFPINFLTLYVTIQHKKLRTPLNYILLNLAVANLFMVLVGFTTTMYTSMNGYFIFGTIGCNVEGFFATLG
Xenopeltis_unicolor_Rh1             MNGTEGLNFYVPMSNKTGIVRSPFEYPQYYLADPWKYSALAAYMFLLILLGFPINFLTLYVTIQHKKLRTPLNYILLNLAVANLFMVLVGFTTTMYTSMNGYFVFGTVGCNVEGFFATLG
Atractus_reticulatus_LWS            --DEDTTRESIFVYTNSNNTRGPFEGPNYHIAPRWVYNLTSLWMVFVVIASVFTNGLVLVATAKFKKLRHPLNWILVNLAIADLGETVIASTISVINQFFGYFVLGHPLCVVEGYTVSVC
Chironius_bicarinatus_LWS           --DEDTTQDSLFAYTNSNNTRDPFEGPNYQIAPRWVYNITSLWMVFVVVASVFTNGLVLVATAKFKKLRHPLNWILVNLAIADLGETVIASTISVINQFFGYFILGHPMCVLEGYTVSAC
Dipsas_petersi_LWS                  --DEDTTRESVFVYTNSNNTRGPFEGPNYHIAPRWVYNLTSLWMVFVVIASVFTNGLVLVATAKFKKLRHPLNWILVNLAIADLGETVIASTISVINQFFGYFVLGHPLCVVEGYTVSVC
Echinantera_cephalostriata_LWS      --DEDTTKDSIFSYTNSNNTRDPFEGPNYHIAPRWVYNITSLWMVFVVVASVFTNGLVLVATAKFKKLRHPLNWILVNLALADLGETVIASTISVINQFFGYFILGHPMCVLEGYTVSAC
Echinantera_undulata_LWS            --DEDTTKDSIFSYTNSNNTRDPFEGPNYHIAPRWVYNITSLWMVFVVVASVFTNGLVLVATAKFKKLRHPLNWILVNLALADLGETVIASTISVINQFFGYFILGHPMCVLEGYTVSAC
Erythrolamprus_aesculapii_LWS       -------------YTNSNNTRDPFEGPNYHIAPRWVYNITSLWMVFVVVASVFTNGLVLVATAKFKKLRHPLNWILVNLAIADLGETVIASTISVINQFFGYFVLGHPLCVLEGYTVSAC
Erythrolamprus_miliaris_LWS         --DEDTTRDSIFAYTNSNNTRDPFEGPNYHIAPRWVYNITSLWMVFVVVASVFTNGLVLVATAKFKKLRHPLNWILVNLAIADLGETVIASTISVINQFFGYFVLGHPLCVLEGYTVSAC
Erythrolamprus_poecilogyrus_LWS     --DEDTTRDSVFAYTNSNNTRDPFEGPNYHIAPRWVYNITSLWMVFVVVASVFTNGLVLVATAKFKKLRHPLNWILVNLAIADLGETVIASTISVINQFFGYFVLGHPLCVLEGYTVSAC
Helicops_modestus_LWS               --DEDTTRDSVFAYTNSNNTRDPFEGPNYHIAPRWVYNLTSLWMVFVVIASVFTNGLVLVATAKFKKLRHPLNWILVNLAIADLGETVIASTISVINQFFGYFILGHPLCVLEGYTVSAC
Oxyrhopus_guibei_LWS                --DEDTTRDSVFVYTNSNNTRDPFEGPNYHIAPRWVYNLTSLWMVFVVIASVFTNGLVLVATAKFKKLRHPLNWILVNLAIADLGETVIASTISVINQFFGYFVLGHPLCVVEGYTVSVC
Philodryas_patagoniensis_LWS        DDDEDTTRDSIFAYTNSNNTRDPFEGPNYHIAPRWVYNITSLWMVFVVVASVFTNGLVLVATAKFKKLRHPLNWILVNLAIADLGETVIASTISVINQFFGYFVLGHPLCVLEGYTVSVC
Python_regius_LWS                   --DDDTTRESVFIYTNSNNTRDPFEGPNYHIAPRWVYNLTSLWMVFVVIASVFTNGLVLVATAKFKKLRHPLNWILVNLAIADLGETVIASTISVINQFFGYFILGHPLCVVEGYTVSVC
Sibynomorphus_mikanii_LWS           --DEDTTRESIFVYTNSNNTRGPFEGPNYHIAPRWVYNLTSLWMVFVVIASVFTNGLVLVATAKFKKLRHPLNWILVNLAIADLGETVIASTISVINQFFGYFVLGHPLCVVEGYTVSVC
Sibynomorphus_neuwiedii_LWS         --DEDTTRESVFVYTNSNNTRGPFEGPNYHIAPRWVYNLTSLWMVFVVIASVFTNGLVLVATAKFKKLRHPLNWILVNLAIADLGETVIASTISVINQFFGYFVLGHPLCVVEGYTVSVC
Spilotes_pullatus_LWS               --DEDTTKDSLFAYTNSNNTRDPFEGPNYHIAPRWVYNITSLWMVFVVIASVFTNGLVLVATAKFKKLRHPLNWILVNLAIADLGETVIASTISVVNQFFGYFILGHPMCVLEGYTVSAC
Taeniophallus_persimilis_LWS        --DEDTTKDSLFSYTNSNNTRDPFEGPNYHIAPRWVYNITSLWMVFVVVASVFTNGLVLVATAKFKKLRHPLNWILVNLALADLGETVIASTISVINQFFGYFILGHPMCVLEGYTVSAC
Thamnodynastes_strigatus_LWS        --DEDTTRDSLFTYTNSNNTRDPFEGPNYHIAPRWVYNVTSLWMIFVVIASVFTNGLVLVATAKFKKLRHPLNWILVNLAIADLGETVIASTISVINQFFGYFYLGHPLCVLEGYTVSVC
Tomodon_dorsatus_LWS                --DEDTTRDSIFTYTNSNNTRDPFEGPNYHIAPRWVYXVTSLWMIFVVIASVFTNGLVLVATAKFKKLRHPLNWILVNLAIADLGETVIASTISVINQFFGYFYLGHPLCVLEGYTVSAC
Xenopeltis_unicolor_LWS             --DDDTTRESVFVYTNSNNTRDPFEGPNYHIAPRWVYNLTSLWMVFVVIASVFTNGLVLVATAKFKKLRHPLNWILVNLAIADLGETVIASTISVINQFFGYFILGHPLCVVEGYTVSVC
Atractus_reticulatus_SWS1           ------------------------------------FHFQTIFMGLVFFAGTPLNAIILFVTIKYKKLRQPLNYILVNISLAGLIFCIFAVFTVFLSSTQGYFFFGRQVCRLEAFLGTVA
Chironius_bicarinatus_SWS1          -----------------------------------------------FFAGTPLNGIILFVTIKYKKLRQPLNYILVNISFAGFMVCIFAILVVFLSSMHGYFFLGRQFCKMEAFLGTVA
Dipsas_petersi_SWS1                 ------------------------DGPQYHIAPMWAFHFQAIFMGLVFFAGTPLNAIILFVTIKYKKLRQPLNYILVNISLAGLIFCIFAVFTVFLSSTQGYFFFGRQVCRLEAFLGTVA
Echinantera_cephalostriata_SWS1     -------------------------------------QFQTIFMGLVFFAGTPLXAIILFVTIKYKKLRQPLNYILVNISFAGFIFCTFAVFVVFLSSSQGYFFFGRQVCRLEAFLGTVA
Echinantera_undulata_SWS1           -----------------------------------VFHFQTIFMGLVFFAGTPLNAIILFVTIKYKKLRQPLNYILVNISFAGFIFCTFAVFVVFLSSSQGYFFFGRQVCRLEAFLGTVA
Erythrolamprus_aesculapii_SWS1      ------------------------DGPQYHIAPKWAFHFQTIFMGLVFFAGTPLNAIILFVTIKYKKLRQPLNYILVNISFAGFIFCIFAVFTVFLSSSQGYFFFGRQVCRLEAFLGTVA
Erythrolamprus_miliaris_SWS1        ------------------------DGPQYHIAPMWAFHFQTIFMGLVFFAGTPLNAIILFVTVKYKKLRQPLNYILVNISFAGFIFCIFAVFTVFLSSSQGYFFFGRQVCRLEAFLGTVA
Erythrolamprus_poecilogyrus_SWS1    ------------------------------------FHFQTIFMGLVFFAGTPLNAIILFVTVKYKKLRQPLNYILVNISFAGFIFCIFAVFTVFLSSSQGYFFFGRQVCRLEAFLGTVA
Helicops_modestus_SWS1              -------------------------GPQYHIAPMWAFHFQTIFMGLVLFAGTPLNAXILFVTIKYKKLRQPLNYILVNISFAGFI?CVFA?F?VFLSSTQGYFFLGRQFC??EAFLGTVA
Oxyrhopus_guibei_SWS1               ------------------------------------FHFQTIFMGLVFFAGTPLNCIILFVTIKYKKLRQPLNYILVNISFAGFIFCTFAVSVVFLSSSQGYFFFGRQVCKLEAFLGTVA
Philodryas_patagoniensis_SWS1       ---------------------------------------------------------------KYKKLRQPLNYILVNISFAGFIFCIFAVFTVFLSSSQGYFFFGRQVCRLEAFLGTVA
Python_regius_SWS1                  ------------YLFENISSVGPWDGPQYHIAPMWAFHFQTLFMGLVFFAGTPLNATILIVTIKYKKLRQPLNYILVNISFAGLLFCVFAVFTVFLASSQGYFFFGRHVCALEAFLGSVA
Sibynomorphus_mikanii_SWS1          ------------------------DGPQYHIAPMWAFHFQAIFMGLVFFAGTPLNAIILFVTIKYKKLRQPLNYILVNISLAGLIFCIFAVFTVFLSSTQGYFFFGRQVCRLEAFLGTVA
Sibynomorphus_neuwiedii_SWS1        ------------------------DGPQYHIAPMWAFHFQAIFMGLVFFAGTPLNAIILFVTIKYKKLRQPLNYILVNISLAGLIFCIFAVFTVFLSSTQGYFFFGRQVCRLEAFLGTVA
Spilotes_pullatus_SWS1              ------------------------------------FHFQTIFMGLVFFAGTPLNAIILFVTIKYKKLRQPLNYILVNISFAGFIFCVFAVFVVFLSSMQGYFFLGRQFCKMDAFLGTVA
Taeniophallus_persimilis_SWS1       --------------------------------------FQTIFMGLVFFAGTPLNAIILFVTIKYKKLRQPLNYILVNISFAGFIFCTFAVFVVFLSSSQGYFFFGRQVCRLEAFLGTVA
Thamnodynastes_strigatus_SWS1       ------------------------------------FHFQTIFMGLVFFAGTPLNAIILFVTIKYKKLRQPLNYILVNISFAGLIFCVFAVFTVFLSSTQGYFFFGRQVCRLEAFLGTVA
Tomodon_dorsatus_SWS1               --------------------VRPWDGPQYHIAPMWAFHFQTIFMGLVFFAGTPLNAIILFVTIKYKKLRQPLNYILVNISFAGLIFCVFAVFTVFLSSTQGYFFFGRQVCRLEAFLGTVA
Xenopeltis_unicolor_SWS1            -----MSGEEDFYLFENISSVGPWDGPQYHIAPMWAFRFQTAFMGLVFFAGTPLNATILIVTIKYKKLRQPLNYILVNISFAGLLFCVFAIFTVFLASSQGYFFFGRHVCALEAFLGSVA
```

```
                                      121       130         140         150         160         170       180         190         200        210          220        230          240
                                       |         |           |           |           |           |         |           |           |          |            |          |            |
                  Bos_taurus_Rh1    GEIALWSLVVLAIERYVVVCKPMSNFRFGENHAIMGVAFTWVMALACAAPPLVGWSRYIPEGMQCSCGIDYYTPHEETNNESFVIYMFVVHFIIPLIVIFFCYGQLVFTVKEAAAQQQES
        Atractus_reticulatus_Rh1    GEIALWSLVVLAVERYVVVCKPMSNFRFTETHAIMGVAFTWIMALACAAPPLVGWSRYIPEGMQSSCGIDYYTPTPEVYNESYVIYMFLVHFIIPLMVIFFCYGRLLCTVKEAAAQQQES
        Chironius_bicarinatus_Rh1   GEIALWSLVVLAVERYVVVCKPMSNFRFTETHAIMGLSLTWIMALACAAPPLIGWSRYIPEGMQSSCGVDYYTPTPEVHNESFVIYMFLVHFVTPLTVIFFCYGRLVCTVKEAAAQQQES
              Dipsas_petersi_Rh1    GEIALWSLVVLAIERYVVVCKPMSNFRFTETHAIMGVAFTWIMALACAAPPLVGWSRYIPEGMQSSCGIDYYTPSPQVYNESFVIYMFIVHFLIPLTVIFFCYGRLLCTVKEAAAQQQES
Echinantera_cephalostriata_Rh1      GEIALWSLVVLAVERYVVVCKPMSNFRFTETHAIMGVSLTWIMALACAAPPLVGWSRYIPEGMQSSCGVDYYTPTPEVYNESFVIYMFLVHFVTPLTIIFFCYGRLLCAVKEAAAQQQES
        Echinantera_undulata_Rh1    GEIALWSLVVLAVERYVVVCKPMSNFRFTETHAIMGVSLTWIMALACAAPPLVGWSRYIPEGMQSSCGVDYYTPTPEVYNKSFVIYMFLVHFVTPLTIIFFCYGRLLCTVKEAAAQQQES
   Erythrolamprus_aesculapii_Rh1    GEIALWSLVVLAVERYVVVCKPMSNFRFTETHAIAGVSLTWIMALACAVPPLIGWSRYIPEGMQSSCGVDYYTPTPEVYNESFVIYMFLVHFVTPLTVIFFCYGRLLCTVKEAAAQQQES
     Erythrolamprus_miliaris_Rh1    GEIALWSLVVLAVERYVVVCKPMSNFRFTETHAIAGVSLTWIMALACAVPPLIGWSRYIPEGMQSSCGVDYYTPTPEVYNESFVIYMFLVHFVTPLTVIFFCYGRLLCTVKEAAAQQQES
 Erythrolamprus_poecilogyrus_Rh1    GEIALWSLVVLAVERYVVVCKPMSNFRFTETHAIAGVSLTWIMALACAAPPLIGWSRYIPEGMQSSCGVDYYTPTPEVYNESFVIYMFLVHFVTPLTVIFFCYGRLLCTVKEAAAQQQES
            Helicops_modestus_Rh1   GEIALWSLVVLAVERYVVVCKPMSNFRFTETHAIMGVSLTWIMALACAAPPLVGWSRYIPEGMQSSCGVDYYTPTPEVYNESFVIYMFLVHFVTPLTVIFFCYGRLLC?VKEAAAQQQES
             Oxyrhopus_guibei_Rh1   GEIALWSLVVLAIERYVVVCKPMSNFRFTETHAIVGVSFTWIMAMACAAPPLVGWSRYIPEGMQTSCGIDYYTPSPEVYNESFVIYMFVVHFMIPLIVIFFCYGRLLCAVKEAAAQQQES
      Philodryas_patagoniensis_Rh1  GEIALWSLVVLAVERYVVVCKPMSNFRFTETHAIMGVSLTWIMALACAAPPLIGWSRYIPEGMQSSCGVDYYTPTPEVYNESFVIYMFLVHFVTPLTVIFFCYGRLLCTVKEAAAQQQES
                 Python_regius_Rh1  GEIALWSLVVLAIERYVVVCKPMSNFRFTETHAIMGLCFTWIMALACAGPPLVGWSRYIPEGMQCSCGVDYYTPTPEVHNESFVIYMFIVHFVIPLVVIFFCYGRLVCTVKEAAAQQQES
        Sibynomorphus_mikanii_Rh1   GEIALWSLVVLAIERYVVVCKPMSNFRFTETHAIMGVAFTWIMALACAAPPLVGWSRYIPEGMQSSCGIDYYTPSPQVYNESFVIYMFVVHFLIPLTVIFFCYGRLLCTVKEAAAQQQES
       Sibynomorphus_neuwiedi_Rh1   GEIALWSLVVLAIERYVVVCKPMSNFRFTETHAIMGVAFTW?MALACAAPPLVGWSRYIPEGMQSSCGIDYYTPSPQVYNESFVIYMFVVHFLIPLTVIFFCYGRLLCTVKEAAAQQQES
            Spilotes_pullatus_Rh1   GEIALWSLVVLAVERYVVVCKPMSNFRFTETHAIMGVSLTWIMALACAAPPLIGWSRYIPEGMQSSCGVDYYTPTPEVHNESFVIYMFLVHFVTPLTVIFFCYGRLVCTVKEAAAQQQES
     Taeniophallus_persimilis_Rh1   GEIALWSLVVLAVERYVVVCKPMSNFRFTETHAIMGVSLTWIMALACAAPPLVGWSRYIPEGMQSSCGVDYYTPTPEVYNKSFVIYMFLVHFVTPLTIIFFCYGRLLCTVKEAAAQQQES
     Thamnodynastes_strigatus_Rh1   GEIGLWSLVVLAVERYVVVCKPMSNFRFTEIHAIMGVSLTWIMALACAAPPLIGWSRYIPEGMQSSCGVDYYTPTPEVYNESFVIYMFLVHFVTPLTIIFFCYGRLLCTVKEAAAQQQES
             Tomodon_dorsatus_Rh1   GEIGLWSLVVLAVERYVVVCKPMSNFRFTETHAIMGVSLTWIMALACAAPPLIGWSRYIPEGMQSSCGVDYYTPTPEVYNESFVIYMFLVHFVTPLTVIFFCYGRLLCTVKEAAAQQQES
          Xenopeltis_unicolor_Rh1   GEMALWSLVVLAIERYVVVCKPMSNFRFTETHAIMGLAFTWIMALSCAGPPLVGWSRYIPEGMQCSCGVDYYTPSPEVQNESFVIYMFIVHFIIPMIVISFCYGRLVCTVKEAAAQQQES
        Atractus_reticulatus_LWS    GITALWSLAIISWERWFVVCKPFGNIKFDAKMAVGGILFSWIWASSWTAPPIFGWSRYWPHGLKTSCGPDVFSGNEDPGVQSYMLTLMITCCVIPLSIIILCYLQVWMAIRAVAAQQKES
        Chironius_bicarinatus_LWS   GITALWSLAIISWERWVVVCKPFGNVKFDAKMALGGIFFSWIWSCAWTAPPIFGWSRYWPHGLKTSCGPDVFSGSEDPGVQSYMVVLMITCCAIPLSVIIICYLQVWMAIRAVAAQQKES
              Dipsas_petersi_LWS    GITGLWSLAIISWERWFVVCKPFGNIKFDAKMAVGGILFSWIWASSWTAPPMFGWSRYWPHGLKTSCGPDVFSGNEDPGVQSYMLTLMITCCIIPLSIIILCYLQVWMAIRAVAAQQKES
Echinantera_cephalostriata_LWS      GITALWSLAIISWERWVVVCKPFGNVKFDAKMAMGGILFSWIWSCAWTAPPIFGWSRYWPHGLKTSCGPDVFSGSEDPGVQSYMVVLMITCCAIPLSVIIICYLQVWLAIRAVAAQQKES
        Echinantera_undulata_LWS    GITALWSLAIISWERWVVVCKPFGNVKFDAKMAMGGILFSWIWSCAWTAPPIFGWSRYWPHGLKTSCGPDVFSGSEDPGVQSYMVVLMITCCAIPLSVIIICYLQVWLAIRAVAAQQKES
   Erythrolamprus_aesculapii_LWS    GITALWSLAIISWERWVVVCKPFGNVKFDAKMALGGIFFSWIWSCAWTAPPVFGWSRYWPHGLKTSCGPDVFSGSEDPGVQSYMVVLMITCCAIPLSVIVICYLQVWLAIRAVAAQQKES
     Erythrolamprus_miliaris_LWS    GITALWSLAIISWERWVVVCKPFGNVKFDAKMALGGIFFSWIWSCAWTAPPVFGWSRYWPHGLKTSCGPDVFSGSEDPGVQSYMVVLMITCCAIPLSVIVICYLQVWMAIRAVAAQQKES
 Erythrolamprus_poecilogyrus_LWS    GITALWSLAIISWERWVVVCKPFGNVKFDAKMAVGGILFSWIWACSWTAPPIFGWSRYWPHGLKTSCGPDVFSGNEDPGVQSYMIVLMTTCCIIPLSVIIICYLQVWMAIRAVAAQQKES
            Helicops_modestus_LWS   GITALWSLAIISWERWVVVCKPFGNVKFDAKMAIGGILFAWIWSCAWTAPPIFGWSRYWPHGLKTSCGPDVFSGNEDPGVQSYMIVLMSTCCIFPLSVIIICYLQVWLAIRAVAAQQKES
             Oxyrhopus_guibei_LWS   GITGLWSLAIISWERWVVVCKPFGNIKFDAKMALGGILFSWIWASTWTAPPIFGWSRYWPHGLKTSCGPDVFSGSEDTGVQSYMIVLMITCCIIPLSIIIICYLQVWLAIRAVAAQQKES
      Philodryas_patagoniensis_LWS  GITALWSLAIISWERWVVVCKPFGNVKFDAKMALGGIFFSWIWSAIWTAPPIFGWSRYWPHGLKTSCGPDVFSGNEDPGVQSYMVVLMITCCAIPLSVIIICYLQVWLAIRAVAAQQKES
                 Python_regius_LWS  GITGLWSLAIISWERWVVVCKPFGNLKFDAKLALAGILFSWVWSATWTAPPIFGWSRYWPHGLKTSCGPDVFSGNEDPGVQSYMIVLMVTCCIIPLSIIVLCYMQVWMAIRAVAAQQKES
        Sibynomorphus_mikanii_LWS   GITALWSLAIISWERWFVVCKPFGNIKFDAKMAVGGILFSWIWASSWTAPPMFGWSRYWPHGLKTSCGPDVFSGNEDPGVQSYMLTLMITCCIIPLSIIILCYLQVWMAIRAVAAQQKES
      Sibynomorphus_neuwiedii_LWS   GITGLWSLAIISWERWFVVCKPFGNIKFDAKMAVGGILFSWIWASSWTAPPMFGWSRYWPHGLKTSCGPDVFSGNDDPGVQSYMLTLMITCCIIPLSIIILCYLQVWMAIRAVAAQQKES
            Spilotes_pullatus_LWS   GITALWSLAIISWERWVVVCKPFGNVKFDAKMALGGIFFSWIWSCVWTAPPIFGWSRYWPHGLKTSCGPDVFSGNEDPGVQSYMIVLMITCCAIPLSVIIICYLQVWMAIRAVAAQQKES
     Taeniophallus_persimilis_LWS   GITALWSLAIISWERWVVVCKPFGNVKFDAKMAMGGILFSWIWSCAWTAPPIFGWSRYWPHGLKTSCGPDVFSGSEDPGVQSYMVVLMITCCAIPLSVIIICYLQVWLAIRAVAAQQKES
     Thamnodynastes_strigatus_LWS   GITALWSLAIISWERWVVVCKPFGNVKFDAKMAIGGILFAWIWAGVWTAPPIFGWSRYWPHGIKTSCGPDVFSGNEDPGVQSYMIVLMSTCCIFPLSVIILCYIQVWLAIRAVAAQQKES
             Tomodon_dorsatus_LWS   GITALWSLAIISWERWVVVCKPFGNVKFDAKMAVGGILFAWIWACTWTAPPIFGWSRYWPHGIKTSCGPDVFSGNEDPGVQSYMIVLMSTCCIFPLSVIIICYIQVWLAIRAVAAQQKES
          Xenopeltis_unicolor_LWS   GITALWSLAIISWERWVVVCKPFGNLKFDAKMALAGILFSWVWSATWTAPPIFGWSRYWPHGLKTSCGPDVFSGNEDPGVQSYMIVLMITCCVIPLSIIILCYLQVWMAIRAVAAQQKES
        Atractus_reticulatus_SWS1   GMVTGWSLAFLAFERYIVICKPLGNFRFHAKHAVVVVATTWVIGVGVAIPPFFGWSRFIPEGLQCSCGPDWYTVGTKYKSEYYSWFLFIFCFIIPLTLIIFSYARLLGALRAVAAQQQES
       Chironius_bicarinatus_SWS1   GMVTGWSLAFLAFERYIVICKPLGNFRFNSKHALVVVATTWIIGIAVSVPPFFGWSRFLPEGLQCSCGPDWYTVGTKYKSEYYSWFLFVFCFFIPFSLIIYSYGRLLSALRAVAAQQQES
             Dipsas_petersi_SWS1    GMVTGWSLAFLAFERYIVICKPLGNFRFNAKHAVVVVATTWVIGVGVAIPPFFGWSRFIPEGLQCSCGPDWYTVGTKYKSEYYSWFLFIFCFIIPLTLIIFSYAQLLGALRAVAAQQQES
Echinantera_cephalostriata_SWS1     GMVTGWSLAFLAFERYIVICKPMGNFRFNAKHALVVVATTWVIGVGVAVPPFFGWSRFLPEGLQCSCGPDWYTVGTKYKSEYYSWFLFIFCFLIPLTLIIFSYARLLGALRAVAAQQQES
       Echinantera_undulata_SWS1    GMVTGWSLAFLAFERYIVICKPMGNFRFNAKHALVVVATTWVIGVGVAVPPFFGWSRFLPEGLQCSCGPDWYTVGTKYKSEYYSWFLFIFCFLIPLTLIIFSYARLLGALRAVAAQQQES
  Erythrolamprus_aesculapii_SWS1    GMVTGWSLAFLAFERYIVICKPLGNFRFNAKHALVVVATTWVIGVGVAIPPFFGWSRFLPEGLQCSCGPDWYTVGTKYKSEYYSWFLFIFCFLIPLTLIIFSYARLLGALRAVAAQQQES
    Erythrolamprus_miliaris_SWS1    GMVTGWSLAFLAFERYIVICKPLGNFRFNAKHALVVVATTWVIGVGVAIPPFFGWSRFLPEGLQCSCGPDWYTVGTKYKSEYYSWFLFIFCFLIPLTLIIFSYARLLGALRAVAAQQQES
Erythrolamprus_poecilogyrus_SWS1    GMVTGWSLAFLAFERYIVICKPLGNFRFNAKHALVVVATTWVIGVGVAIPPFFGWSRFLPEGLQCSCGPDWYTVGTKY?SEYYSWFLFIFCFLIPLTLIIFSYARLLGALRAVAAQQQES
           Helicops_modestus_SWS1   GMVTGWSLAFLAFERYIVICKP?GSFRFNAKHAMVVVA?TWLIGVGVALPPFFGWSRFVPEGLQCSCGPDWYTVGTKYKSEYYSWFLFVFCFLIPL?LIIFSYARLLGALRAVAAQQQES
            Oxyrhopus_guibei_SWS1   GMVTGWSLAFLAFERYIVICKPLGNFRFNAKHAVVVVATTWVIGVGVAVPPFFGWSRFLPEGLQCSCGPDWYTVGTKYKSEYYSWFLFIFCFLIPLTLIIFSYAQLLGALRAVAAQQQES
     Philodryas_patagoniensis_SWS1  GMVTGWSLAFLAFERYIVICKXMGNFRFXGKHALVVVATTWVIGVGVALPPFFGWSRFXPEGLQCSCGPDWYTVGTKYKSEYYSWFLFIFCFLIPLTLIIFSYARLLGALRAVAAQQQES
               Python_regius_SWS1   GLVTGWSLAFLAFERYIVICKPLGNFRFNSKHALLVVATWVIGVGVSVPPFFGWSRFIPEGLQCSCGPDWYTVGTKYKSEYYSWFLFVFCFIVPLSLIVFSYLRLLGALRAVAAQQQES
       Sibynomorphus_mikanii_SWS1   GMVTGWSLAFLAFERYIVICKPLGNFRFNAKHAVVVVATTWVIGVGVAIPPFFGWSRFIPEGLQCSCGPDWYTVGTKYKSEYYSWFLFVFCFIIPLTLIIFSYARLLGALRAVAAQQQES
    S ibynomorphus_neuwiedii_SWS1   GMVTGWSLAFLAFERYIVICKPLGNFRFNAKHAVVVVATTWVIGVGVAIPPFFGWSRFIPEGLQCSCGPDWYTVGTKYKSEYYSWFLFIFCFIIPLTLIIFSYARLLGALRAVAAQQQES
           Spilotes_pullatus_SWS1   GLVTGWSLAFLAFERYIVICKPLGNFRFNSKHALVVVATTWIIGIGVSVPPFFGWSRFLPEGLQCSCGPDWYTVGTKYKSEYYSWFLFIFCFLIPLTLIIYSYARLLGALRAVAAQQQES
    Taeniophallus_persimilis_SWS1   GMVTGWSLAFLAFERYIVICKPMGNFRFNAKHALVVVATTWVIGVGVAVPPFFGWSRFLPEGLQCSCGPDWYTVGTKYKSEYYSWFLFIFCFLIPLTLIIFSYARLLGALRAVAAQQQES
    Thamnodynastes_strigatus_SWS1   GMVTGWSLAFLAFERYIVICKPMGNFRFNAKHAVVVVATTWVIGVGVAIPPFFGWSRFLPEGLQCSCGPDWYTVGTKYKSEYYSWFLFIFCFLIPLTLIIFSYAQLLGALRAVAAQQQES
            Tomodon_dorsatus_SWS1   GMVTGWSLAFLAFERYIVICKPMGNFRFNAKHAVVVVATTWIIGVGVAIPPFFGWSRFLPEGLQCSCGPDWYTVGTKYKSEYYSWFLFIFCFLIPLTLIIFSYAQLLGALRAVAAQQQES
         Xenopeltis_unicolor_SWS1   GLVTGWSLAFLAFERYVVICKPLGNFRFNAKHALLVVAATWVIGVGVSIPPFFGWSRFIPEGLQCSCGPDWYTVGTKYKSEYYSWFLFVFCFIVPLSLIVFSYLQLLRALRAVAAQQQES
```

```
                                    241       250       260       270       280       290       300       310       320       330       340       350       360
                                    |         |         |         |         |         |         |         |         |         |         |         |         |
Bos_taurus_Rh1                      ATTQKAEKEVTRMVIIMVIAFLICWLPYAGVAFYIFTHQGSDFGPIFMTIPAFFAKTSAVYNPVIYIMMNKQFRNCMVTTLCCGKNPLGDDEASTTVSKTETSQVAPA*-----------
Atractus_reticulatus_Rh1            ATTQKAEKEVTRMVILMVIAFLVCWVPYASVAFYIFTHQGSDFGPVFMTIPAFFAKSSAIYNPVIYIVMNKQFRNCMITTLCCGKNPLA-----------------------------
Chironius_bicarinatus_Rh1           ATTQKAEKEVTRMVILMVIAFLVCWVPYASVAFYIFTHQGSDFGPVFMTIPSFFAKSSAIYNPVIYIVMNKQFRNCMLTTLCCGKNPLVEDDTSV-GTKTETSTVSTS------------
Dipsas_petersi_Rh1                  ATTQKAEKEVTRMVILMVIAFLVCWVPYASVAFYIFTHQGSDFGPVFMTIPAFFAKSSSIYNPVIYIVMNKQFRNCMITTLCCGKNPLAEDDTSA-GTKTETSTVSTSKVS---------
Echinantera_cephalostriata_Rh1      ATTQKAEKEVTRMVILMVIAFLVCWVPYASVAFYIFTHQGSDFGPVFMTIPSFFAKSSAIYNPVIYIVMNKQFRNCMLTTLCCGKNPLAEDDTSA-GTKTETSTVSTSKVF---------
Echinantera_undulata_Rh1            ATTQKAEKEVTRMVILMVIAFLVCWVPYASVAFYIFTHQGSDFGPVFMTIPSFFAKSSAIYNPVIYIVMNKQFRNCMLTTLCCGKNPLAEDDTSA-GTKTETSTVSTSKVS---------
Erythrolamprus_aesculapii_Rh1       ATTQKAEKEVTRMVILMVIAFLICWVPYASVAFYIFTHQGSDFGPVFMTIPSFFAKSSAIYNPVIYIVMNKQFRNCMLTTLCCGKNPLAEDDTSA-GTKTETSTVSTSKV----------
Erythrolamprus_miliaris_Rh1         ATTQKAEKEVTRMVILMVIAFLICWVPYASVAFYIFTHQGSDFGPVFMTIPSFFAKSSAIYNPVIYIVMNKQFRNCMLTTLCCGKNPLAEDDTSA-GTKTETSTVSTSKV----------
Erythrolamprus_poecilogyrus_Rh1     ATTQKAEKEVTRMVILMVIAFLVCWVPYASVAFYIFTHQGSDFGPVFMTIPSFFAKSSAIYNPVIYIVMNKQFRNCMLTTLCCGKNPL------------------------------
Helicops_modestus_Rh1               ATTQKAEKEVTRMVILMVIAFLVCWVPYASVAFYIFTHQGSDFGPVFMTIPSFFAKSSAIYNPVIYIVMNKQFRNCMLTTLCCGKNPLAEDDTSA-GTKTETSTVSTSKVF---------
Oxyrhopus_guibei_Rh1                ATTQKAEKEVTRMVILMVIAFLVCWVPYASVAFYIFTHQGSDFGPVFMTAPAFFAKSSSIYNPVIYIVMNKQFRNCMITTLCCGKNPLGEEDTSA-GTKTETSTVSTSKVS---------
Philodryas_patagoniensis_Rh1        ATTQKAEKEVTRMVILMVIAFLVCWVPYASVAFYIFTHQGSDFGPVFMTIPSFFAKSSAIYNPVIYIVMNRQFRNCMLTTLCCGKNPLAEDDTSA-GTKTETSTVSTSKVS---------
Python_regius_Rh1                   ATTQKAEKEVTRMVIIMVIAFLICWVPYASVAFYIFTHQGSDFGPVFMTLPAFFAKSSAIYNPVIYIVLNKQFRNCMITTLCCGKNPLAEDDTSA-GTKTETSTVS-------------
Sibynomorphus_mikanii_Rh1           ATTQKAEKEVTRMVILMVIAFLVCWVPYASVAFYIFTHQGSDFGPVFMTIPAFFAKSSAIYNPVIYIVMNKQFRNCMITTLCCGKNPLAEDDTSA-GTKTETSTVST------------
Sibynomorphus_neuwiedi_Rh1          ATTQKAEKEVTRMVILMVIAFLVCWVPYASVAFYIFTHQGSDFGPVFMTIPAFFAKSSSIYNPVIYIVMNKQFRNCMITTLCCGKNPLAEEDTSA-GTKTETSTVSTS-----------
Spilotes_pullatus_Rh1               ATTQKAEKEVTRMVILMVVAFLVCWVPYASVAFYIFTHQGSDFGPVFMTIPSFFAKSSAIYNPVIYIVMNKQFRDCMLTTLCCGKNPLAEDDTSA-GTKTETSTVST------------
Taeniophallus_persimilis_Rh1        ATTQKAEKEVTRMVILMVIAFLVCWVPYASVAFYIFTHQGSDFGPVFMTIPSFFAKSSAIYNPVIYIVMNKQFRNCMLTTLCCGKNPLAEDDTSA-GTKTETSTVSTS-----------
Thamnodynastes_strigatus_Rh1        ATTQKAEKEVTRMVILMVIAFLVCWVPYASVAFYIFTHQGSDFGPVFMTIPSFFAKSSAIYNPVIYIVMNKQFRNCMLTTLCCGKNPLAEDDTSA-GTKTETSTVSTS-----------
Tomodon_dorsatus_Rh1                ATTQKAEKEVTRMVILMVIAFLVCWVPYASVAFYIFTHQGSDFGPVFMTIPSFFAKSSAIYNPVIYIVMNKQFRNCMLTTLCCGKNPLAEDDTSA-GTKT-------------------
Xenopeltis_unicolor_Rh1             ATTQKAEKEVTRMVVIMVIAFLICWVPYASVAFYIFTHQGSDFGPVFMTLPAFFAKSSAIYNPVIYIVLNKQFRNCMITTLCCGKNPLAEDDTSA-GTKTETSTVSTSQVSPA*------
Atractus_reticulatus_LWS            ESTQKAEKEVSRMVVVMIIAYIICWGPYASFACFAAANPGYAFHPLTASLPAFFAKSATIYNPIIYVFMNRQFRNCIMQLFGQES-----------------------------------
Chironius_bicarinatus_LWS           ESTQKAEKEVSRMVVVMIIAYIVCWGPYTFFACFAVANPGYAFHPLTASMPAFFAKSATIYNPIIYVFMNRQFRNCIMQLX---------------------------------------
Dipsas_petersi_LWS                  ESTQKAEKEVSRMVVVMIIAYIICWGPYASFACFAAANPGYAFHPLTASLPAFFAKSATIYNPIIYVFMNRQFRNCIMQLFGKKVX----------------------------------
Echinantera_cephalostriata_LWS      ESTQKAEKEVSRMVVVMIIAYIVCWGPYTFFACFAVANPGYAFHPLTASMPAFFAKSATIYNPIIYVFMNR-----------------------------------------------
Echinantera_undulata_LWS            ESTQKAEKEVSRMVVVMIIAYIVCWGPYTFFACFAVANPGYAFHPLTASMPAFFAKSATIYNPIIYVFMNRQFRNCIM------------------------------------------
Erythrolamprus_aesculapii_LWS       ESTQKAEKEVSRMVVVMIIAYIICWGPYTFFACFAVANPGYAFHPLTASMPAFFAKSATIYNPIIYVFMNRQFRNC--------------------------------------------
Erythrolamprus_miliaris_LWS         ESTQKAEKEVSRMVVVMIIAYIVCWGPYTFFACFAVANPGYAFHPLTASMPAFFAKSATIYNPIIYVFMNRQFRNCIMQLCG--------------------------------------
Erythrolamprus_poecilogyrus_LWS     ESTQKAEKEVSRMVVVMIIAYIVCWGPYTFFACFAVANPGYAFHPLTASMPAFFAKSATIYNPIIYVFMNRQFRNCIMQLF---------------------------------------
Helicops_modestus_LWS               ESTQKAEKEVSRMVVVMIIAYIVCWGPYTFFACFAAANPGYAFHPLAASLPAFFAKSATIYNPIIYVFMNRQFRNCIMQLF---------------------------------------
Oxyrhopus_guibei_LWS                ESTQKAEKEVSRMVVVMIIAYIVCWGPYTFFACFAVANPGYAFHPLTASMPAFFAKSATIYNPIIYVFMNRQFRNCIMQLFGKKV-----------------------------------
Philodryas_patagoniensis_LWS        ESTQKAEKEVSRMVVVMIIAYIVCWGPYTFFACFAVANPGYAFHPLTASMPAFFAKSATIYNPIIYVFMNRQFRNCIMQLFG--------------------------------------
Python_regius_LWS                   ESTQKAEKEVSRMVVVMILAYIFCWGPYTTFACFAAANPGYAFHPMTASLPAFFAKSATIYNPIIYVFMNRQFRNCIMQLFGKKVDDGSEVSSTSRTEVSSVS-----------------
Sibynomorphus_mikanii_LWS           ESTQKAEKEVSRMVVVMIIAYIICWGPYASFACFAAANPGYAFHPLTASLPAFFAKSATIYNPIIYVFMNSQ----------------------------------------------
Sibynomorphus_neuwiedii_LWS         ESTQKAEKEVSRMVVVMIIAYIICWGPYASFACFAAANPGYAFHPLTASLPAFFAKSATIYNPIIYVFMNRQFRNCIMQLF---------------------------------------
Spilotes_pullatus_LWS               ESTQKAEKEVSRMVVVMIIAYIVCWGPYTFFACFAAANPGYAFHPLTASIPAFFAKSATIYNPIIYVFMNRQFRNC--------------------------------------------
Taeniophallus_persimilis_LWS        ESTQKAEKEVSRMVVVMIIAYIVCWGPYTFFACFAVANPGYAFHPLTASMPAFFAKSATIYNPIIYVFMNRQFRNCIM------------------------------------------
Thamnodynastes_strigatus_LWS        ESTQKAEKEVSRMVVVMIIAFIVCWGPYTFFACFAAANPGYAFHPLTASMPAFFAKSATIYNPIIYVFMNRQFRNCIMQLF---------------------------------------
Tomodon_dorsatus_LWS                ESTQKAEKEVSRMVVVMIIAYIVCWGPYTFFACFAVANPGYAFHPLTASMPAFFAKSATIYNPIIYVFMNRQFRNCIMQLFAKKV-----------------------------------
Xenopeltis_unicolor_LWS             ESTQKAEKEVSRMVVVMILAYIVCWGPYTTFACFAAANPGYAFHPMTASLPAFFAKSATIYNPIIYVFMNRQFRNCIMQLFGKKVDDGSEVSSTSRTEVSSVSNSSVSPA*---------
Atractus_reticulatus_SWS1           ATTQKAEREVSRMVVVMVASFCICYVPYAALAMYMVNNPHHGFDLRLVTIPAFFSKSSCVYNPIIYCFMNKQFRACIMETVX-GKPITDDSDVSX-XQKTXVSSASSCQVS---------
Chironius_bicarinatus_SWS1          ATTQKAEREVSRMVVVMVGSFGMCYIPYAALAMYMVNNPQHNLDLRLVTIPAFFSKSSCVYNPIIYCFMNKQFRACIMETVC-GKPMTDDSDAVX-XQKTEVSSASSCKVSP--------
Dipsas_petersi_SWS1                 ATTQKAEREVSRMVVVMVASFCICYVPYAALAMYMVNNPHHGFDLRFVTIPAFFSKSSCVYNPIIYCFMNKQFRACIMETVC-GKPMTDESDLSX-XQKTEVSSASSCQVSX--------
Echinantera_cephalostriata_SWS1     ATTQKAEREVSRMVVVMVGSFCVCYVPYAALAMYMVNNPQHGFDLRLVTIPAFFSKSSCVYNPIIYCFMNKQFRACIMETVC-GKPMTDESDVT-------------------------
Echinantera_undulata_SWS1           ATTQKAEREVSRMVVVMVGSFCVCYVPYAALAMYMVNNPQHGFDLRLVTIPAFFSKSSCVYNPIIYCFMNKQFRACIMETVC-GKPMTDDSDVTX-XSK--------------------
Erythrolamprus_aesculapii_SWS1      ATTQKAEKEVSRMVVVMVGSFCVCYVPYAALAMYMVNNPQHGFDLRLVTIPAFFSKSSCVYNPIIYCFMNKQFRACIMETVC-GKPMTDESDAT-------------------------
Erythrolamprus_miliaris_SWS1        ATTQKAEREVSRMVVVMVGSFCICYVPYAALAMYMVNNPQHGFDLRLVTIPAFFSKSSCVYNPIIYCFMNKQFRACIMETVC-GKPMTDDSDVX-------------------------
Erythrolamprus_poecilogyrus_SWS1    ATTQKAEREVSRMVVVMVGSFCICYVPYAALAMYMVNNPQHGFDLRLVTIPAFFSKSSCVYNPIIYCFMNKQFRACIME-----------------------------------------
Helicops_modestus_SWS1              ATTQKAEREVSRMVVVMVGSFCVCYVPYAALAMYMVNNPQHG?DLRLVTIPAFFSKSSCVYNP?IYCFMNKQFRACIMETVC-GKPMTDDSDVSX-XQRTEVSSASSCQVS---------
Oxyrhopus_guibei_SWS1               ATTQKAEREVSRMVVVMVGSFCVCYVPYAAMAMYMVNNPQHGFDLRLVTIPAFFSKSSCVYNPIIYCFMNKQFRACIMEMVC-GK------------------------------------
Philodryas_patagoniensis_SWS1       ATTQKAEREVSRMVVVMVGSFCICYVPYAALAMYMVNNPQHGFDLRLVTIPAFFSKSSCVYNPIIYCFMNKQFRACI-------------------------------------------
Python_regius_SWS1                  ATTQKAEREVSRMVVVMVGSFCLCYVPYATLAMYMVNHPKHGLDLRLVTIPAFFSKSSCVYNPIIYCFMNKQFRACIMQTVC-GKPLTEESDAGSSAQKTEVSSVSSSQVSPSSGAG---
Sibynomorphus_mikanii_SWS1          ATTQKAEREVSRMVVVMVASFCICYVPYAALAMYMVNNPHHGFDLRLVTIPAFFSKSSCVYNPIIYCFMNKQFRACIMETVC-GKPMTDESDLX-------------------------
Sibynomorphus_neuwiedii_SWS1        ATTQKAEREVSRMVVVMVASFCICYVPYAALAMYMVNNPHHGFDLRLVTIPAFFSKSSCVYNPIIYCFMNKQFRACIMETVC-GKPMMDESDVSX-XQKTEVSSV---------------
Spilotes_pullatus_SWS1              ATTQKAEREVSRMVVVMVGSFCICYIPYAALAMYMVNNPQHGLDLRLVTIPAFFSKSSCVYNPIIYCFMNKQFRACIMETVC-GKPMTDDSDVT-------------------------
Taeniophallus_persimilis_SWS1       ATTQKAEREVSRMVVVMVGSFCVCYVPYAALAMYMVNNPQHGFDLRLVTIPAFFSKSSCVYNPIIYCFMNKQFRACIMETVC-GKPMTDESDVT-------------------------
Thamnodynastes_strigatus_SWS1       ATTQKAEREVSRMVVVMVGSFCVCYVPYAALAMYMVNNPQHGFDLRLVTIPAFFSKSSCVYNPIIYCFMNKQFRACIMETVC-GKPMTDDSDV--------------------------
Tomodon_dorsatus_SWS1               ATTQKAEREVSRMVVVMVGSFGVCYVPYAALAMYMVNNPQHGFDLRLVTIPAFFSKSSCVYNPIIYCFMNKQFRACIMETVC-GKPMTDDSDVTX-XQKTEVSSVSSCQVSP--------
Xenopeltis_unicolor_SWS1            ATTQKAEREVSRMVVVMVGSFCLCYVPYAALAMYMVNHPKHGLDLRLVTIPAFFSKSSCVYNPIIYCFMNKQFRSCIMETVC-GKPLTDESDAGSSVQKTEVSSVSSCQVSPSSGAGTTG
```
